# Identification and Expression Analysis of microRNAs at the Grain Filling Stage in Rice(*Oryza sativa* L.)via Deep Sequencing

**DOI:** 10.1371/journal.pone.0057863

**Published:** 2013-03-01

**Authors:** Rong Yi, Zhixuan Zhu, Jihong Hu, Qian Qian, Jincheng Dai, Yi Ding

**Affiliations:** State Key Laboratory of Hybrid Rice, Department of Genetics, College of Life Sciences, Wuhan University, Wuhan, People’s Republic of China; University of Delhi South Campus, India

## Abstract

MicroRNAs (miRNAs) have been shown to play crucial roles in the regulation of plant development. In this study, high-throughput RNA-sequencing technology was used to identify novel miRNAs, and to reveal miRNAs expression patterns at different developmental stages during rice (*Oryza sativa* L.) grain filling. A total of 434 known miRNAs (380, 402, 390 and 392 at 5, 7, 12 and 17 days after fertilization, respectively.) were obtained from rice grain. The expression profiles of these identified miRNAs were analyzed and the results showed that 161 known miRNAs were differentially expressed during grain development, a high proportion of which were up-regulated from 5 to 7 days after fertilization. In addition, sixty novel miRNAs were identified, and five of these were further validated experimentally. Additional analysis showed that the predicted targets of the differentially expressed miRNAs may participate in signal transduction, carbohydrate and nitrogen metabolism, the response to stimuli and epigenetic regulation. In this study, differences were revealed in the composition and expression profiles of miRNAs among individual developmental stages during the rice grain filling process, and miRNA editing events were also observed, analyzed and validated during this process. The results provide novel insight into the dynamic profiles of miRNAs in developing rice grain and contribute to the understanding of the regulatory roles of miRNAs in grain filling.

## Introduction

Rice (*Oryza sativa* L.) grain filling is a highly coordinated developmental process. During this period, large amounts of storage compounds are synthesized and transported into the rice endosperm, which are major determinants of the economic value of rice grain and provide nutrients and calories for humans and many other animals. Extensive studies on the mechanisms underlying this process have been carried out in the past two decades. It has been documented that transcription control is a primary mechanism for determining endosperm development [1]. Several enzymes interact with certain key transcription factors to regulate the transcription of nutrient partitioning genes during grain filling, at each developmental stage. Both the participating enzymes and reserve compounds are expressed in appropriate amounts and are tightly regulated both spatially and temporally [2], [3]. Phytohormones are also considered to play important roles in plant development. It has been reported that appropriate concentrations of ethylene, IAA and abscisic acid (ABA) can increase the rate of reserve compound synthesis, leading to higher grain yields [4], [5]. Proteomic and cDNA microarray analyses revealed that the products of grain filling-related genes are associated with several important processes, including biosynthesis, metabolism, transportation, the response to stimuli and signal transduction [3], [6]. These findings, together with observations of the morphological changes that occur rice grain during the filling process [6], [7], suggest that the accumulation of reserves involves multiple metabolic and regulatory pathways, and the expression of the genes in different pathways is coordinately regulated in a timely manner between different developmental stages during grain filling. In spite of this, the genes and underlying molecular mechanisms controlling rice grain filling remain elusive.

Endogenous small regulatory RNAs are a large family of negative regulators that mediate eukaryotic gene expression at the transcriptional and posttranscriptional levels [8]. Among these small molecular RNAs, microRNAs (miRNAs) (21∼22 nt) function as regulators of developmentally timed events, while short-interfering RNAs (siRNAs) (21–28 nt) are responsible for RNA interference (RNAi) and transcriptional silencing, such as through genome rearrangement, histone and DNA methylation and chromatin modification [8], [9]. In plants, most predicted miRNA targets are known or putative transcriptional factors with functions in development, pointing to a role for miRNAs at the core of gene regulation networks [10]. miRNAs function to regulate diverse developmental process, including meristem and lateral organ development, root initiation, flowering and sex determination, timing and phase transitions [10], [11]. More than 500 miRNAs have been identified in rice and deposited in miRBase (v18.0) to date. Some miRNAs play important roles in the adaptive response to abiotic stress in tissues (drought, cadmium or H_2_O_2_-responsive) [12]–[14], and some have been preferentially identified in different rice tissue at diverse developmental stages [15]–[17]. Several studies have been carried out on small RNAs in rice grain, demonstrating dynamic compositions and expression profiles of miRNAs at different developmental stages, suggesting a regulatory role of miRNAs during grain development [17]–[21].

To further study the complicated miRNA regulatory network during this process, we used high-throughput technology to survey the differences in the composition and expression profiles of miRNAs among different developmental stages during grain filling. According to previous research, rice grains require approximately 30 days to fully mature. Reserve compounds begin to accumulate in the endosperm at 5 days after fertilization (DAF), which represents the onset of grain filling. The length of the grain reaches a plateau at 7 DAF, and the size of the grain reaches a maximum at 12 DAF, when the mid-maturation stage begins. The fresh weight of grain continues to increase until 17 DAF, when developing grains enter into the desiccation stage [6], [7]. Therefore, we sequenced small RNAs from rice grains collected at 5DAF, 7DAF, 12DAF and 17DAF to determine the differential expression pattern of known miRNAs and identify novel miRNAs. The potential targets of differentially expressed miRNAs were analyzed and found to be involved in multiple metabolic and cellular processes during grain development. miRNA editing events were also observed, analyzed and validated. The results deepen our understanding of the important regulatory function of miRNAs in grain filling.

## Materials and Methods

### Plant Materials

Rice (*Oryza sativa* L. cv Nipponbare) was grown in soil in an experimental field at the Wuhan University Institute of Genetics (Wuhan, China) (latitude 30°34″N; longitude 114°17″E). Rice grains at different developmental stages, including: 5 DAF, 7 DAF, 12 DAF and 17 DAF, were collected, frozen in liquid nitrogen and stored at −80°C for further use.

### Small RNA Library Construction, Sequencing and Data Analysis

Total RNA was isolated from rice grains using the pBIOZOL reagent (Bioer Technology, China) according to the manufacturer’s instructions. After separation via 15% polyacrylamide gel electrophoresis (PAGE), the 18–30 nucleotide fraction was purified, and a pair of Solexa adapters was ligated to the 5′ and 3′ termini. The resultant products were reverse-transcribed and amplified through 15 PCR cycles to produce sequencing libraries. Solexa sequencing was performed by the Beijing Genomics Institute (BGI) according to the manufacturer’s protocols.

After Solexa sequencing, the adaptor sequences and low quality tags were trimmed. Sequences ranging from 18 to 30 nt that could be aligned to the rice genome (MSU 6.1) (ftp://ftp.plantbiology.msu.edu/pub/data/Eukaryotic_Projects/o_sativa/annotation_dbs/pseudomolecules/version_6.1/) were collected for further analyses. Sequences that mapped to rice rRNA, scRNA, snoRNA, snRNA or tRNA sequences in the National Center for Biotechnology Information (NCBI) (http://www.ncbi.nlm.nih.gov/) and Rfam RNA family databases [22] were removed. RepeatMasker software (http://www.repeatmasker.org/) was used to filter out sequences originating from repeat regions. Sequences overlapping with exons and introns in the mRNAs were identified as degraded fragments of mRNAs and excluded from subsequent analyses. Sequences that could be perfectly mapped onto miRNA precursors and mature miRNAs in the miRBase miRNA database (v17.0) (http://mirbase.org/) were identified as known miRNAs.

The characteristic hairpin structure of miRNA precursors can be used to predict novel miRNAs. MIREAP (http://sourceforge.net/projects/mireap/) miRNA prediction software, developed by BGI, was used to predict novel miRNA candidates by exploring the secondary structure, Dicer cleavage sites and the minimum free energy of the unannotated small RNA tags that could be mapped to the genome. The program was run with following parameters: (1) minimal miRNA sequence length, 18 nt; (2) maximal miRNA sequence length, 25 nt; (3) maximal free energy allowed for an miRNA precursor, −18 kcal/mol; (4) maximal space between an miRNA and miRNA*, 300; (5) minimal base pairs of miRNA and miRNA*, 16; (6) maximal bulge of miRNA and miRNA*, 4; (7) maximal asymmetry of miRNA/miRNA* duplexes, 4; (8) flank sequence length of miRNA precursors, 20. Finally, the criteria proposed by Wei et al. [23] were used to identify novel miRNAs. First, to decrease background noise, small RNAs with a read number of less than 5 were filtered out. Second, small RNAs mapped to multiple loci in the rice genome were excluded. Third, small RNAs transcribed from both strands of the genome, which would generate siRNA-like small RNAs were eliminated. Because the precursors of all known rice miRNAs do not contain repetitive sequences, the sequences of candidate precursors were analyzed using RepeatMasker to eliminate homologs of repetitive sequences. (http://www.repeatmasker.org). The sequences of all miRNAs were used to query *Oryza sativa* MSU Rice Genome Annotation release 6.1 (MSU 6.1) to predict potential target genes using psRNA Target with the default parameters (http://plantgrn.noble.org/psRNATarget/).

### miRNA Editing Analysis and Cloning of Novel miRNAs

Positions 2∼8 of a mature miRNA, which are referred to as the seed region, are highly conserved. Changes in the nucleotides in this region could cause alteration of a miRNA’s target. In the present analysis, miRNAs potentially presenting base editing were detected by aligning unannotated sRNAs with mature miRNAs in miRBase, allowing one mismatch at a certain position.

To identify miRNA editing and clone novel miRNAs from rice grain during the grain filling stage, total RNA was isolated from rice grain using the TRIzol reagent and then treated with DNase I (NEB, USA). DNA-free RNA (5 µg) was reverse-transcribed using miRNA-specific stem-loop primers (50 nM) in a 20-ul reaction volume with the Fermentas RevertAid First Strand cDNA Synthesis Kit (Fermentas, USA). DNA was also extracted from the rice grain. The RT-PCR conditions for miRNA amplification and the PCR conditions for pre-miRNA sequence amplification were as follows: 95°C for 4 min, followed by 40 cycles of 94°C for 1 min, 55°C for 50 s and 72°C for 10 s, with a final extension at 72°C for 10 min. The obtained PCR products were detected via gel electrophoresis. For miRNA editing analysis, a minimum of nine clones were sequenced for each miRNA PCR product, and three clones for pre-miRNA. All of the primers used are listed in Table S1.

### Differential Expression and Quantitative RT-PCR Analysis of miRNAs

To investigate the expression patterns of miRNAs among the four different developmental stages, the read count of each identified miRNA was normalized to transcripts per million (TPM) using the following formula: normalized expression = actual miRNA count/total count of clean reads×1000000. After normalization, the expression was set to 0.01 for miRNAs that were not expressed in one of two samples. If the miRNAs expression was <1 in both samples, differential expression analysis was not performed. The differential expression analysis was carried out adopting a threshold of a fold change>2.0 (the log2 scale value) between two adjacent developmental stages. miRNAs showing a significantly differential expression profile (*P*<0.01) were clustered into different groups using GeneCluster 3.0.

Total RNA was extracted from grain at each developmental stage. RNase-free DNase I (NEB, USA) was used to remove DNA contamination for 45 min at 37°C. Approximately 5 µg of total RNA was reverse-transcribed using miRNA-specific stem-loop primers (50 nM) in a 20-ul reaction volume with the Fermentas RevertAid First Strand cDNA Synthesis Kit (Fermentas, USA).The reactions were incubated for 30 min at 16°C, followed by 60 cycles of pulsed RT of at 30°C for 30 s, 42°C for 30 s and 50°C for 1 s to increase the sensitivity of miRNA detection [24]. The reactions were terminated by heating at 70°C for 5 min. Stem-loop primers were designed according to Chen *et al.* [25]. cDNA templates for miRNA targets were synthesized using oligo-dT primers with the Fermentas RevertAid First Strand cDNA Synthesis Kit (Fermentas, USA) according to the manufacturer’s instructions. U6 snRNA and β-actin were chosen as internal controls for the miRNAs and the miRNA targets, respectively.

The expression patterns of the miRNAs and their potential target genes were analyzed using the ABI Step One Plus Real-Time PCR System (Applied Biosystems, USA) with the SYBR® *Premix Ex Taq*™ kit (Takara, Japan). The miRNA cDNAs were diluted 100 times and 2 µl diluted product was mixed with 10 µl of 2 × SYBR reaction mix and a 0.2 µM concentration of each of the miRNA-specific forward and universal reverse primers in a 20-ul PCR amplification mixture. cDNAs for target genes were diluted 20 times, and 1 µl of diluted product was used as a template in a 10-ul PCR amplification mixture. Triplicate quantitative assays were performed with the following cycling parameters: 30 s at 95°C, followed by 40 cycles of 10 s at 95°C, 30 s at 56°C and 15 s at 72°C, and the results were represented as the mean ±SD of three replicates. Melting curve analysis was carried out for each PCR product to avoid nonspecific amplification. The comparative Ct method was used to calculate the fold changes in the miRNAs and their targets in different samples. The primers used in all quantitative RT-PCR experiments are listed in Table S1.

### miRNA Target Functional Analysis

Gene Ontology (GO), the *de facto* standard for gene functionality description, is widely accepted for use in most large-scale gene annotation projects. Here, potential targets of novel miRNAs and known miRNAs showing significantly differential expression patterns between two samples (5 DAF-7 DAF, 7 DAF-12 DAF and 12 DAF-17 DAF) were subjected to GO functional enrichment analysis using AgriGO with *Oryza sativa* MSU6.1 as the suggested background reference [26]. Graphical results for overrepresented GO terms were generated via singular enrichment analysis to fully understand the function of these targets.

### RNA Ligase-mediated 5′RACE

Total RNA (2 µg) from rice grain was ligated directly to the 5′RACE adapter using the 5′-Full RACE Kit (Takara, Japan) according to the manufacturer’s instructions. The reverse transcription product was amplified with outer gene-specific primers through 20 cycles of PCR. A total of 35 cycles of second-round PCR were further performed using the above PCR product as a template with inner gene-specific primers. The final PCR products were gel-purified, cloned (pMD18-T vector, Takara) and sequenced. A minimum of six clones were sequenced for each PCR product. The outer and inner gene-specific primers are listed in Table S1.

## Results

### Overview of Deep Sequencing Datasets

**Figure 1:**
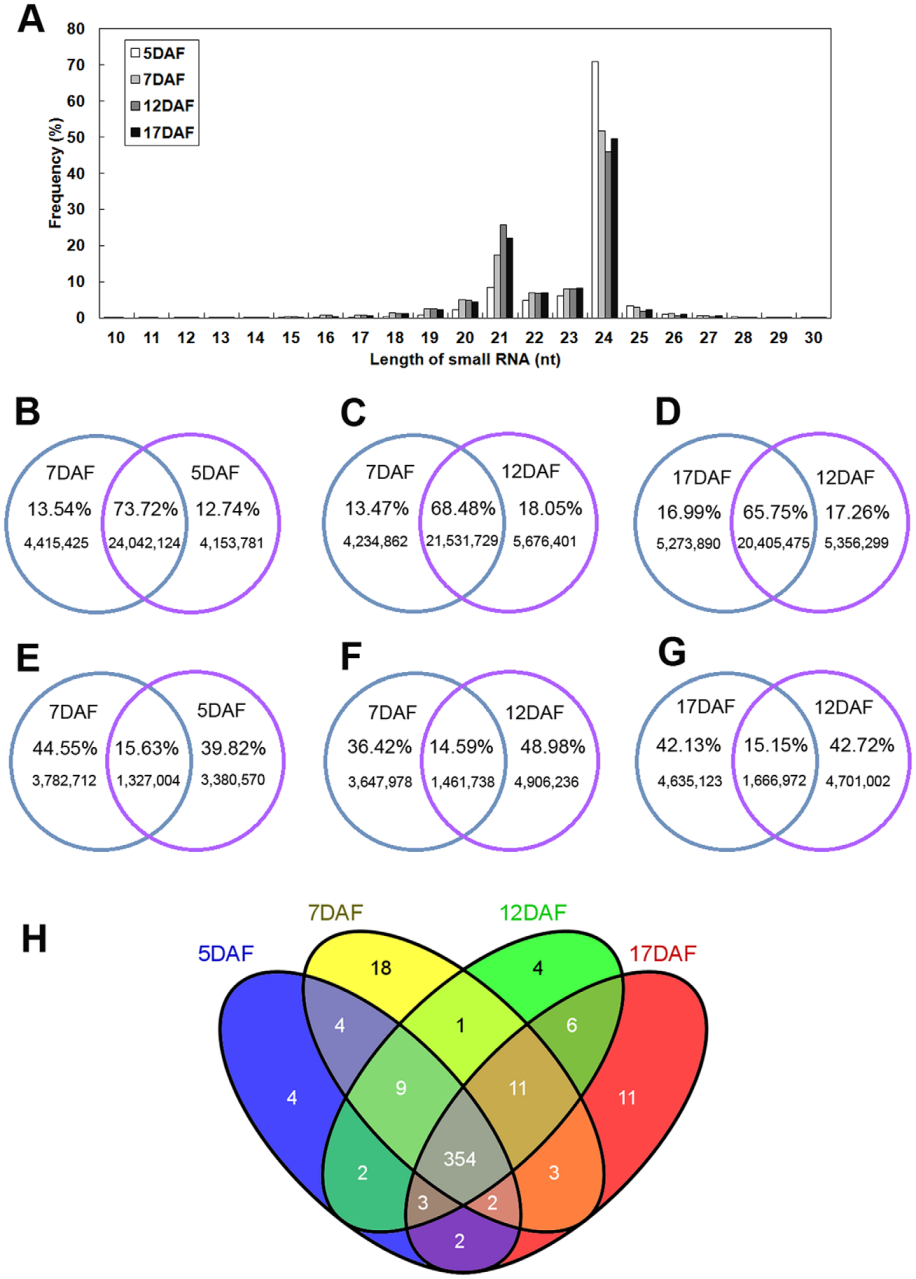
Small RNAs in each of the four stages of rice grain development. (A) Length distribution of small RNAs at different grain development stages. (B-G) Summary of common and specific total (B, C and D) and unique (E, F and G) small RNA sequences between different libraries. (H) Known miRNAs among different libraries.

Total RNA was isolated from developing rice grains collected at 5 DAF, 7 DAF, 12 DAF and 17 DAF to construct four libraries and then subjected to Solexa (now Illumina Inc.) high-throughput RNA-sequencing to determine the expression profiles of miRNAs among the different libraries. The total numbers of clean reads, ranging from 18 to 30 nucleotides in length, were yielded from each of four libraries after precluding the low quality reads, 3′ adaptor and 5′ contaminant sequences were as follows: 17029030 (5 DAF), 15582300 (7 DAF), 15860692 (12 DAF) and 15174972 (17 DAF). These reads corresponded to 4707574, 5109716, 6367974 and 6302095 unique sRNA sequences in the 5 DAF, 7 DAF, 12 DAF and 17 DAF libraries, respectively (Table 1). Of the millions of high-quality sRNAs obtained, 92.71% (5 DAF), 89.25% (7 DAF), 91.37% (12 DAF) and 91.27% (17 DAF) were 20–24 nt in length with 24 and 21 nt representing the major size classes, consistent with the size of products trimmed by Dicer-like(DCL) [27] (Fig. 1A).

**Table 1:** Distribution of small RNAs among different categories.

Category	5DAF		7DAF		12DAF		17DAF	
	Unique sRNAs	Total sRNAs	Unique sRNAs	Total sRNAs	Unique sRNAs	Total sRNAs	Unique sRNAs	Total sRNAs
Total(clean)	4707574(100%)	17029030(100%)	5109716(100%)	15582300(100%)	6367974(100%)	15860692(100%)	6302095 (100%)	15174972(100%)
match_genome	3670743(77.98%)	15561105(91.38%)	4264360(83.46%)	14321820(91.91%)	5470464(85.91%)	14454514(91.13%)	5417780(85.97%)	13707910(90.33%)
miRNA	6129(0.13%)	651290(3.82%)	7255(0.14%)	1374266(8.82%)	8245(0.13%)	2450748(15.45%)	7120(0.11%)	1724461(11.36%)
siRNA	204284(4.34%)	2071100(12.16%)	140816(2.76%)	915973(5.88%)	117516(1.85%)	564923(3.56%)	101584(1.61%)	445278(2.93%)
rRNA	68797(1.46%)	834858(4.90%)	101454(1.99%)	2001144(12.84%)	70404(1.11%)	885193 (5.58%)	92539(1.47%)	1310619(8.64%)
snRNA	2578(0.05%)	8392(0.05%)	2636(0.05%)	12164(0.08%)	1588(0.02%)	3765(0.02%)	1649(0.03%)	4979(0.03%)
snoRNA	4348(0.09%)	11898(0.07%)	4117(0.08%)	9137(0.06%)	3142(0.05%)	5373(0.03%)	2531(0.04%)	4070(0.03%)
tRNA	12375(0.26%)	244390(1.44%)	14750(0.29%)	474879(3.05%)	14654(0.23%)	401166(2.53%)	14482(0.23%)	423497(2.79%)
Repeat	1344549(28.56%)	3465734(20.35%)	1641597(32.13%)	3423279(21.97%)	2269893(35.65%)	4447541(28.04%)	2271866(36.05%)	4543119(29.94%)
Exon_antisence	125600(2.67%)	645276(3.79%)	161670(3.16%)	729322(4.68%)	210993(3.31%)	924274(5.83%)	205936(3.27%)	798123(5.26%)
Exon_sence	197626(4.20%)	753018(4.42%)	257760(5.04%)	657653(4.22%)	309246(4.86%)	629469(3.97%)	315164(5.00%)	603255(3.98%)
Intron_antisence	134049(2.85%)	582606(3.42%)	142854(2.80%)	384622(2.47%)	181547(2.85%)	319280(2.01%)	182120(2.89%)	301734(1.99%)
Intron_sence	139087(2.95%)	580316(3.41%)	156079(3.05%)	435601(2.80%)	200409(3.15%)	406011(2.56%)	197842(3.14%)	382147(2.52%)
No_annotation^#^	2468152(52.43%)	7180152(42.16%)	2478728(48.51%)	5164260(33.14%)	2980337(46.80%)	4822949(30.41%)	2909262(46.16%)	4633690(30.54%)

Using SOAP software [28], 91.38%, 91.91%, 91.13% and 90.33% of the total sRNA sequences, corresponding to representing 77.98%, 83.46%, 85.91% and 85.97% of the unique sRNAs from 5 DAF, 7 DAF, 12 DAF and 17 DAF libraries, were mapped onto the rice genome (MSU6.1), respectively (Table 1). Almost every category of RNA, including miRNA, siRNA, rRNA, snoRNA, snRNA, tRNA, repeat-associated sRNA, and degraded fragments of mRNA introns or exons, was detected in this study. Known rice miRNAs accounted for 3.82%, 8.82%, 15.45% and 11.36% of the sequence reads in the 5 DAF, 7 DAF, 12 DAF and 17 DAF libraries, respectively, indicating that mature miRNAs were relatively enriched in the 12 DAF library. Overall, regarding the common and specific reads of sRNAs between two adjacent libraries, greater than 60% of the total sRNAs were common to two different libraries, which represented only a relatively small fraction (14%–15%) of the unique sequence reads, suggesting that there was a less abundant but much more diverse pool of small RNAs that could be assumed to represent stage-specific small RNAs (Fig. 1B, C, D, E, F, G). These data highlight the differences and complexities in the miRNA reservoir between the different developmental stages.

Due to the relatively stable base pairs at its 5′ end, miRNA* is usually thought to degrade rapidly when the cognate miRNA is selectively incorporated into effector complexes (known as miRNP) for target recognition [29], but a recent study in plants has shown that both miRNA and miRNA* can be selected and can silence different targets regardless of their thermodynamic stability [30]. Here, a few cases where the miRNA* was sequenced more frequently than the miRNA were observed when the sequencing frequency and distribution of small RNAs originating from precursor sequences were examined, suggesting that the miRNA* might be the genuine product of the pre-miRNA or that both the miRNA and miRNA* are functional in regulating gene expression (Figure S1A).

Similar to other deep sequencing studies [14], [17], many variants within a ±2nt range from annotated miRNA sequences were produced from the two arms of the miRNA precursors. In some cases, the sequencing frequencies for these miRNA variants were higher than for corresponding known miRNAs deposited in miRBase, and only the miRNA* of the certain variants was detected (Figure S1B). In some cases, the most abundant miRNA was neither the annotated miRNA nor the miRNA* or its variants (Figure S1C). These phenomena can be explained in part by the sequencing errors; that is to say, the detected sRNAs are only random degradation products from unprocessed precursors. It is also possible that these sRNAs may be the authentic miRNAs, which should be substituted for the annotated miRNAs deposited in miRBase. In addition, it was also observed that some single miRNA precursors produced two, or even more different miRNAs and that the same miRNA was generated from two distinct precursors (Figure S1D, E). These data indicate the existence of complex post-transcriptional processing in these miRNA genes.

To date, 581 rice miRNAs, representing over 100 families, have been cloned or predicted through small RNA library sequencing and included in the miRBase database (v18.0), out of which 20 miRNA families are conserved in both *Arabidopsis* and rice [17]. In present study, 434 known miRNAs (380, 402, 390 and 392 in the 5 DAF, 7 DAF, 12 DAF and 17 DAF libraries, respectively) were detected during the rice grain filling process, and all 20 of the conserved families were included in the obtained dataset (Table S2). Among these miRNAs, 354 were found to be shared by all four RNA libraries, which accounted for 81.57% of the 434 known miRNAs, while 397 miRNAs were expressed in at least two of our four small seed RNA libraries, and 37 miRNAs were detected only once among the four samples (Fig. 1H). These results indicate that a rapidly developing rice grain employs a large proportion of the known miRNAs and, to a certain extent, that the sequencing depth achieved here was sufficient to reflect the expression profiles of miRNAs during grain development.

Among the 20 conserved miRNA families, osa-miR156 and osa-168 were the most abundant miRNA families observed during grain development, accounting for 80.45% of expressed miRNA reads. osa-miR166 and osa-miR167 also showed high expression in the four libraries, suggesting that they are not only conserved among species but also among rice grain developmental stages. In contrast, the read numbers obtained for many non-conserved miRNAs were much lower than those registered for conserved miRNAs in the datasets, especially for the rice-specific miRNAs, such as osa-miR2906, and osa-miR531 and osa-miR810, which presented less than 50 TPM in the four libraries. There were a few exceptions; four rice-specific miRNAs, osa-miR1861, osa-miR1862, osa-miR812, and osa-miR820, displayed relatively high expression during grain filling, suggesting that they may be important regulators of rice grain development.

### Prediction of Novel miRNAs

**Figure 2:**
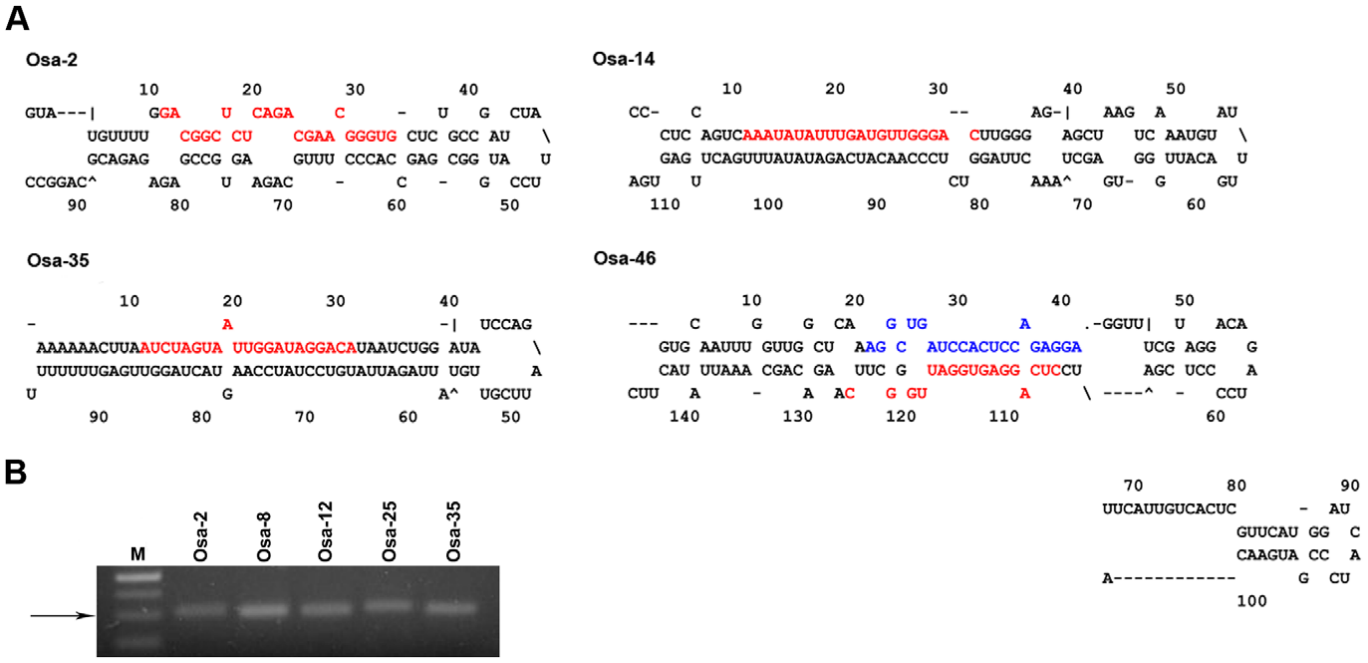
Predicted novel miRNAs identified in this study. (A) Predicted stem-loop structures of novel miRNA precursors. The precursor structures of four newly identified rice miRNAs (Osa-2, Osa-14, Osa-35 and Osa-46) were predicted via the MFOLD pipeline. Mature miRNA and miRNA* sequences are highlighted in red and blue, respectively. The numbers along the structure indicate nucleotide sites from the 5′ end of the pre-miRNAs sequence. (B) Stem-loop RT-PCR analysis if the identified novel miRNAs. Five novel miRNAs were confirmed via stem-loop RT-PCR. The sizes of the obtained PCR products were approximately ∼60 bp. M indicates a 20 bp DNA Ladder Marker (Takara, Japan). The arrow indicates 60 bp.

One of the most important advantages of high-throughput sequencing technology is that it can produce a large volume of data up to the gigabase level during small RNA sequencing, which is helpful for detecting novel miRNAs with extremely low expression levels. In this study, a total of 60 predicted novel miRNAs were obtained (Table S3), fifty-six of which have not been deposited in miRBase (v18.0) and have never been detected in *Oryza sativa* or *Arabidopsis thaliana,* while 4 of these miRNAs were reported during preparation of this draft [20], [23], [31]. The structures of the precursors of all of the novel miRNAs were predicted using MFOLD (http://mfold.rna.albany.edu/) and checked manually. Four of these structures are presented (Fig. 2A). The novel miRNAs were temporarily named following the Osa-number format, e.g., Osa-1, before being submitted to obtain an official designation.

Of the 60 predicted novel miRNAs, 11 miRNAs were expressed in all four libraries, while 30 were detected in 5 DAF, 44 in the 7 DAF, 47 in the 12 DAF and 36 in the 17 DAF libraries (Table S3). Comparing the number of known miRNAs expressed in four libraries, the 5 DAF library exhibited the lowest abundance of both novel miRNAs and known miRNAs (30 and 380, respectively), which implied that more miRNAs remain to be revealed in the 5 DAF stage.

As previously reported, the most important rule of novel miRNA annotation is to detect miRNA* sequences corresponding to mature miRNAs; alternatively, in miRNA*-deficient cases, miRNAs should be detected from multiple, independent libraries [32]. In the present study, each of the novel miRNAs could be sequenced in two or more libraries, while only 12 miRNA* sequences were detected. This finding could be due to the rapid degradation rate of miRNA*. Interestingly, Osa-44 showed similar abundance in terms of both miRNA and miRNA*(read number of 22 vs. 20 in 7 DAF library), suggesting that both the miRNA and miRNA* might be functional in regulating gene expression.

To validate the predicted novel miRNAs, the expression of five novel miRNAs whose sequencing counts exceeded 100 in at least one library were selected to be further confirmed using stem-loop RT-PCR. As a result, all five selected novel miRNAs were found to be expressed in rice grains, suggesting that the computational filters used here were sufficiently strict for predicting novel miRNAs (Fig. 2B).

### miRNA Expression Profiles at Different Developmental Stages

**Figure 3:**
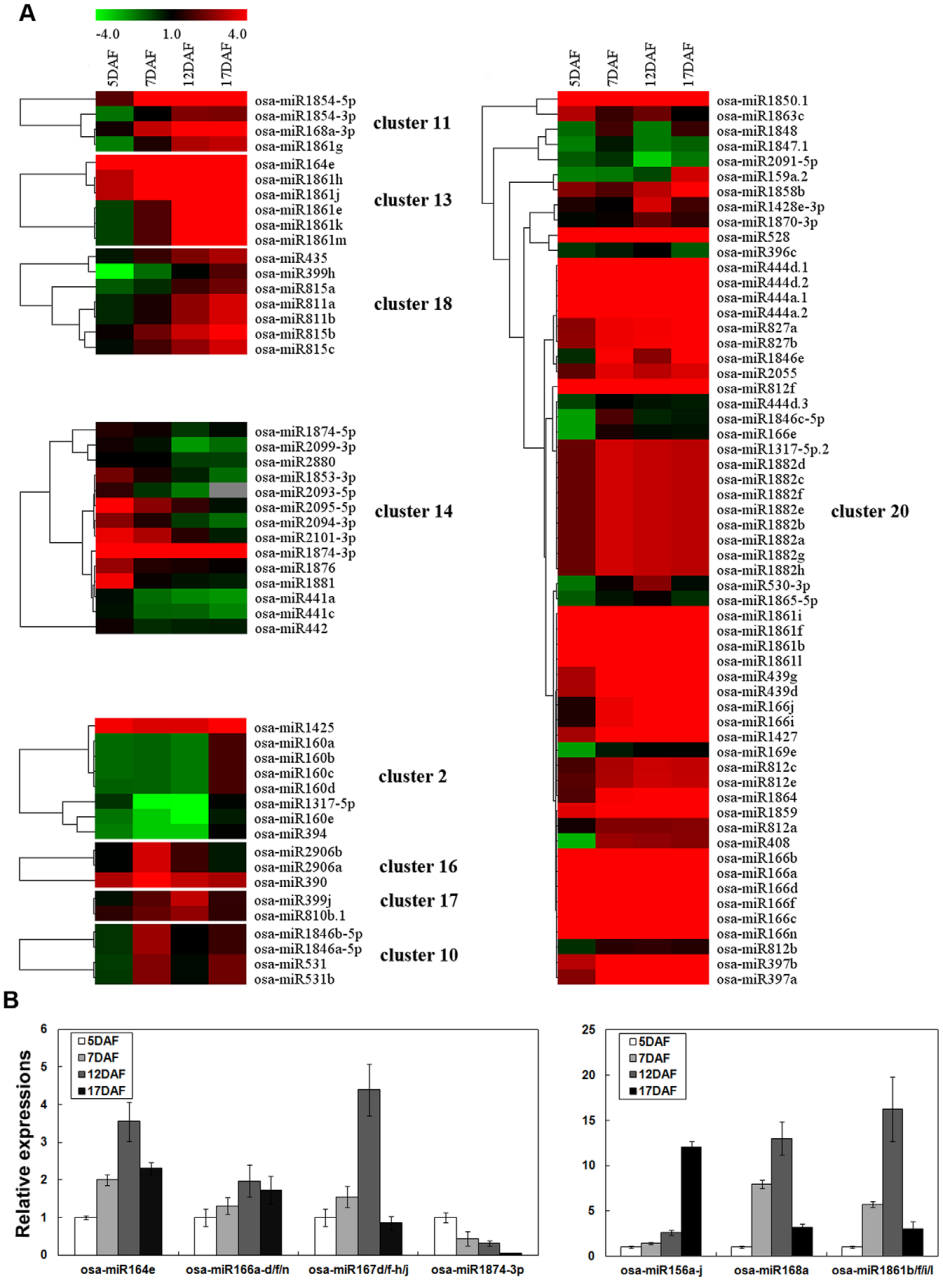
Differential expression analysis of known miRNAs. (A) Heatmap for clustering analysis of the differentially expressed known miRNAs. The bar represents the scale of the expression levels of the miRNAs (log 2). (B) Validation via quantitative real-time RT-PCR of differentially expressed miRNAs obtained from deep sequencing. U6 snRNA was used as a reference, and the expression levels of each of the miRNAs were then compared with the expression at 5 DAF, which was set to 1.0. Error bars indicate the standard deviation (±SD) of three replicates.

Based on the normalized read count for each identified miRNA, differential expression analysis was performed, and 161 known rice miRNAs were found to show statistically significant (*P*<0.01) changes in their relative abundance in at least one of the three transitions (5 DAF to 7 DAF/7 DAF to 12 DAF/12 DAF to 17 DAF) during rice grain filling (Table S4), the majority of which are non-conserved miRNAs. A total of 161 known miRNAs were subjected to cluster analysis according to their expression profiles at different seed filling stages (Figure S2). The results showed that a large numbers of miRNAs were up-regulated during the 5 DAF to 7 DAF periods (cluster 20 in Fig. 3A). Some miRNAs were up-regulated gradually during grain filling (clusters 11, 13 and 18 in Fig. 3A), while some, in cluster 14, appeared to show a similar trend of down-regulation over the same period (Fig. 3A). Some of the miRNAs were preferentially expressed in at least one grain filling stage. For example, the miRNAs in clusters 2, 16 and 17 showed patterns that were enriched at 17 DAF, 7 DAF and 12 DAF, respectively, while the miRNAs in cluster 10 accumulated and peaked at 7 DAF, decreased greatly at 12 DAF, and were up-regulated thereafter (Fig. 3A).

High-throughput sequencing technology makes it possible to detect novel miRNAs with a low abundance. In this study, most of the predicted novel miRNAs were expressed at an extremely low level (<100 reads) compared to the total volume of sequencing data obtained (approximately 15 million clean reads per library, Table 1). The observed abundances ranged from 5 reads to 422 reads, and most of novel miRNAs could not be detected at 5 DAF. Compared with the majority of the known miRNAs expressed constitutively among the four grain filling libraries, most of the novel miRNAs showed differential expression between the four stages analyzed, even with low abundances, suggesting a role for the novel miRNAs that is different from that of the known miRNAs (Table S3).

Because of the difficulty of cloning miRNAs with a lower abundance, seven known miRNAs (osa-miR156a-j, osa-miR164e, osa-miR166a-d/f/n, osa-miR167d/f-h/j, osa-miR168a, osa-miR1861b/f/i/l and osa-miR1874-3p) whose sequencing counts exceeded 100 in at least one library were selected to corroborate the expression profiles obtained from Solexa sequencing using stem-loop, real-time RT-PCR quantification. The expression profiles generated for most of the selected miRNAs were the same as those determined by Solexa sequencing, indicating that the sequencing data produced in this study were reliable and could be subjected to further analysis (Fig. 3B and Table S5A).

### Target Prediction,Correlation with miRNA Expression and Functional Analysis

The major challenge in elucidating the functions of miRNAs is to identify their regulatory targets. Here, we conducted searches of degradome data in starBase (sRNA target Base) [33] to identify the targets of the known and novel miRNAs. Based on the degradome data, we found 2613 targets for 275 known miRNAs. The targets of the remaining 159 known miRNAs were predicted using psRNA Target with the default parameters, and 688 targets for 142 miRNAs were obtained (Table S6). Two hundred and sixty-six targets (22 targets for 14 miRNAs from the degradome data and 244 targets for 41 miRNAs using psRNA Target) for 55 novel miRNAs were obtained via the same method (Table S8).

Similar to other studies, many of the predicted target genes for the known miRNAs encode transcription factors belonging to various families. Furthermore, targets involving methylation, transportation, disease resistance and other biological metabolic pathways were also represented (Table S6). For the predicted targets of the 161 differentially expressed miRNAs, enrichment analysis were performed using AgriGO; of the1089 predicted genes, 432 were categorized into 78 significant GO terms (P<0.01) (Table S7, S9 and Figure S3). Among these terms, binding (GO:0005488), was dominant within the main category of molecular function, corresponding to 49.5% of 432 genes, and transcription factor activity (GO:0003700) was also found to be statistically significant in the same category (Table S9 and Figure S3B). Additionally, a high percentage of the target genes were involved in cellular processes (GO: 0044260, GO:0044237 and GO:0009987) and metabolic processes (GO:0043170, GO:0008152 and GO:0044238). GO terms related to various biological processes, including regulation of metabolic process (GO: 0019222), gene expression (GO: 0010468), biological process (GO: 0050789), RNA metabolic process (GO: 0051252) and transcription (GO: 0045449), RNA biosynthetic process (GO: 0032774), and the response to stimulus (GO: 0009719, GO: 0010033 and GO: 0009725), were also found to be significantly enriched among the known miRNAs (Table S9 and Figure S3A).

The targets of the novel miRNAs exhibited a much broader spectrum of potential functions. In addition to a few transcription factors, targets encoding various enzymes and transposable elements as well as functionally unknown transcripts were observed (Table S8). Surprisingly, no significant GO terms were found to be associated with the targets of the novel miRNAs. The lack of significant GO terms might be due to 1) the biological function diversity of the novel miRNA targets; or 2) the fact that the targets of these novel miRNAs have not been annotated in the rice genome.

**Figure 4:**
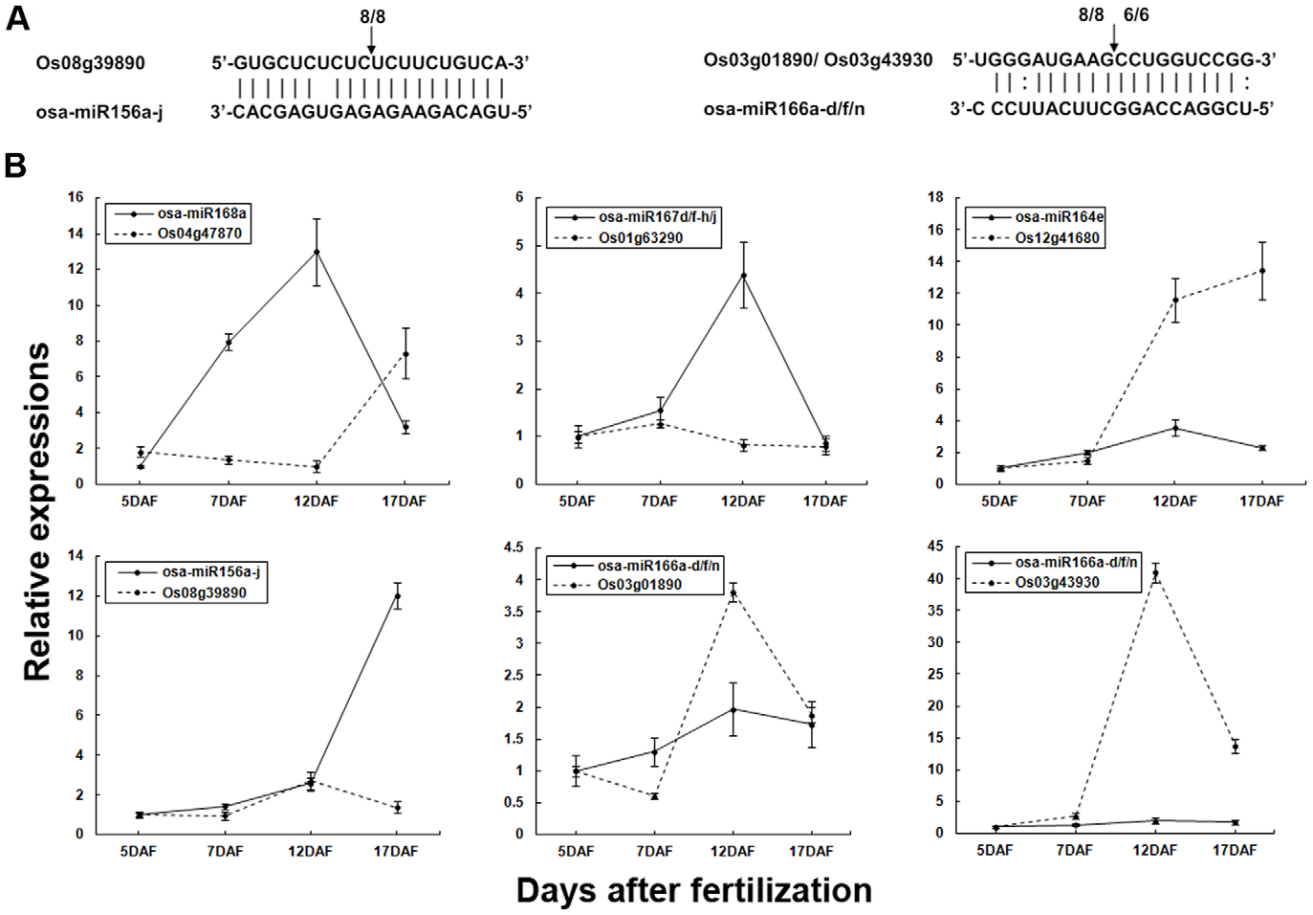
Validation and expression of selected miRNA target genes. (A) 5′-RLM-RACE analysis of the cleavage of target mRNAs by corresponding miRNAs. The arrows indicate the cleavage sites, and the numbers represent the frequency of the sequenced clones. (B) Expression profiling analysis of several target genes and their corresponding miRNAs in rice grain on different days after fertilization. Actin was used as a reference, and the expression levels of each of the target mRNAs were then compared with their expression at 5 DAF or 12 DAF, which was set to 1.0. Error bars indicate the standard deviation (±SD) of three replicates. *Os04g47870* (PINHEAD) and *Os12g41680* (no apical meristem protein) were confirmed to be targets of Osa-miR168 and Osa-miR164, respectively, in previous work [59]. *Os01g63290* (transporter) was predicted to be target of osa-miR167d/f-h/j using psRNA Target (data not shown).

Among the predicted targets of the miRNAs, cleavage events associated with three transcripts of the targets were chosen to be validated by 5′-RACE. Similar to other studies, the mRNAs of the SBP-box gene family member OsSPL14 (*Os08g39890*) and START domain-containing protein genes (*Os03g01890* and *Os03g43930*) were cleaved within the complementary regions of osa-miR156a-j and osa-miR166a-d/f/n, respectively, and were precisely terminated at the 10^th^ position relative to the 5′ end of the complementary regions of osa-miR156a-j and osa-miR166a-d/f/n (Fig. 4A).

The expression patterns of six miRNA target genes were examined via QPCR to investigate whether they were correlated with the levels of the corresponding miRNAs (Table S5B and Fig. 4B). Quite typically, the expression profiles of osa-miR168a and osa-miR167d/f-h/j were negatively correlated with those of their targets, *Os04g47870* (PINHEAD) and *Os01g63290* (transporter), respectively, which is in accordance with the cleavage function of miRNAs. In other cases, the accumulations of miRNAs presented an imperfect positive correlation with their targets. The transcripts of *Os12g41680* (no apical meristem protein) were up-regulated during grain development and were positively correlated with the levels of osa-miR164e in general, consistent with previous findings [19]. A similar correlation was also observed between osa-miR156a-j and its target *Os08g39890* (OsSPL14). The positive correlation between miRNAs and their targets may be due to the feedback mechanisms associated with miRNA regulation [34], or there may be other mechanism of gene expression regulation in addition to regulation of miRNAs in the grain filling stage.

*Os03g01890* and *Os03g43930*(START domain-containing protein genes)are both confirmed to be the targets of osa-miR166a-d/f/n. Here, the expression patterns of these two targets were different during grain development; the transcripts of *Os03g01890* were inversely correlated with the accumulation of osa-miR166a-d/f/n only from 5 DAF to 7 DAF, while the mRNAs of *Os03g43930* were positively correlated with the levels of miR166a-d/f/n during grain filling. This phenomenon indicated that the level of regulation applied to a given miRNAs related to diverse target transcripts could be different.

### Detection of Single-nucleotide Substitutions in miRNA Sequences

**Figure 5:**
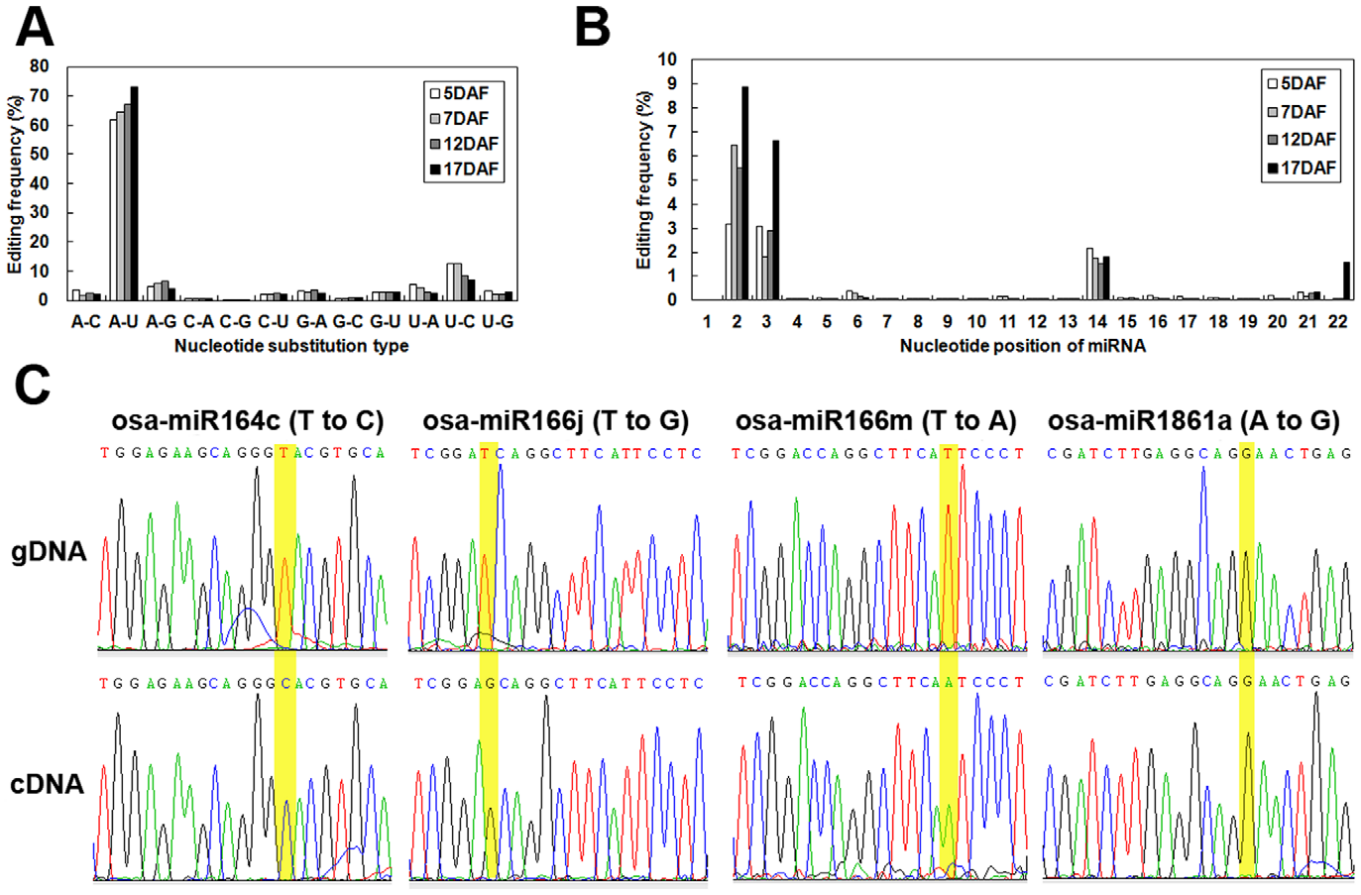
miRNA editing analysis. (A) Summary of the nucleotide substitution types observed in each library. (B) Summary of nucleotide substitution positions among miRNAs. (C) Validation of the editing sites inferred from deep sequencing via Sanger sequencing. Sequencing chromatogram traces from four miRNA sequences are shown. The edited positions are highlighted with yellow shading. The top trace is genomic DNA (gDNA), and the bottom trace is cDNA.

Through analyzing the sequencing data from the four small RNA libraries produced in this study, a large number of miRNA editing events were found (Table S10). The sequencing results revealed that miRNAs and their variants exhibit many types of site-specific nucleotide substitutions and the average rate of each type of nucleotide substitutions found in each library was similar (Fig. 5A). In contrast to the four most common nucleotide substitutions observed in miRNAs from *Arabidopsis*, which are C to U (9.7%), A to G (16.4%), G to A (24.2%) and U to C (12.6%) [35], in the present study, the most dominant nucleotide substitution type was A to U (over 60% in each library) because the mutation rate of A to U at position 14 of the miR156k variant was much higher compared to other forms of nucleotide substitutions (Table S10 and Fig. 5A). To understand whether the detected miRNA editing occurs in vivo, or was caused by DNA SNPs and mismatches that took place during data analysis and other technical artifacts, pre-miRNAs sequence cloned from rice genomic DNA were compared to mature miRNAs obtained from rice cDNA. Two types of nucleotide substitutions, high-level editing (90%–100%) and median-level editing (60%–70%), were chosen to be tested in the 5 DAF sample. pre-miRNAs and mature miRNAs for osa-miR164c, osa-miR166j, osa-miR166m and a 3′-end deletion variant of osa-miR1861a (the most dominant small RNA forms in our study) were selected to confirm the miRNA editing events observed in this study. The miRNA RT-PCR sequencing results showed that all four of these edited miRNAs or variants exhibit the same nucleotide substitutions at the same sites as in the deep sequencing data. The DNA sequencing results showed there were no SNPs detected between the reference and miRNA genes, except for the osa-miR1861a variant (Fig. 5C). These results indicate that the miRNA editing results obtained from Solexa sequencing are reliable, and the detected miRNA editing is genuine and occurs during the grain filling process.

## Discussion

In the past few years, many studies have been devoted to examining the miRNA regulation mechanisms underlying different plant development processes, including grain development in rice. For example, Zhu et al. (2008) used deep sequencing, computational and molecular methods to identify, profile, and describe 56 known miRNA families and 39 novel non-conserved miRNA families in a 1–10 DAF rice grain development library [17]. Xue et al. (2009) identified 26 novel, 12 candidate and 241 known miRNAs via massively parallel signature sequencing (MPSS) of short RNAs from 3–12 DAF rice grain [19]. More recently, both deep sequencing and microarray analyses were used to analyze an miRNA library generated from 5–20 DAF rice grain, and 102 known miRNAs and 11 novel miRNAs were identified [21]. However, all of these miRNAs were obtained from a mixture of grains collected at different stages. In this study, using a deep sequencing approach, small RNAs in developing rice grain from four individual stages: 5 DAF, 7 DAF, 12 DAF and 17 DAF, which cover the major morphological changes that occur during the grain filling process, were analyzed at the genome-wide level. More than sixty million high quality RNAs from four rice samples were obtained, which is a much greater abundance than found in previous studies. Additionally, miRNA editing events were observed, analyzed and, for the first time, validated in rice seeds during grain filling process. The present results revealed a dynamic regulatory mechanism mediated by miRNAs during rice grain development.

### Small RNA Populations Exhibit Dynamic Changes during the Grain Filling Stage

Among the four libraries generated here, sRNA sequences with a length of 24 nt were found significantly more frequently compared to other lengths, and second highest abundance was found for sequences of 21nt (Fig. 1A). In plants, 21 nt miRNAs and trans-acting siRNAs mediate endogenous gene silencing at the post-transcriptional level by guiding mRNA degradation or translational inhibition [36], [37], while 24 nt siRNAs direct DNA and histone modification, leading to transcriptional gene silencing [9], [36], [38], and have also been found mediate DNA cytosine methylation [39]. Recent studies have shown that silencing events in *Arabidopsis* pollen are associated with the loss of most 24nt siRNAs and a dramatic gain of a novel class of 21nt siRNAs. [40]. In this study, the size distributions of small RNAs were found to vary between the libraries. A decrease of 24 nt siRNAs relative to 21nt miRNAs was observed to occur in a stage-specific manner, progressing from 5 DAF, to 7 DAF, to 17 DAF, and to 12 DAF, indicating that miRNAs may regulate target gene expression via different mechanisms at different stages. This finding suggests that transcriptional regulation is the major mechanism in the early stage of grain filling, while post-transcriptional regulation is more crucial in the late stage of grain filling in rice (Fig. 1A).

### Identification of Known miRNAs and Novel miRNAs

In this study, 434 known miRNAs from 149 miRNA families were identified during the grain filling process, which represented a greater abundance compared with previous research [17], [19], [21]. Most of these miRNAs were found to be expressed in all four libraries. Only 37 miRNAs (4, 18, 4 and 11 in the 5 DAF, 7 DAF, 12 DAF and 17 DAF libraries, respectively) were expressed in a stage-specific manner, indicating that the composition of miRNAs during grain filling was relatively stable (Fig. 1H). Using Z-score transformation [23], with a ratio >2.0 and Z-score cutoffs >2.0, we identified 81 known miRNAs that showed a preferentially differential expression pattern among the four libraries (14, 27, 13 and 27 miRNAs in the 5 DAF, 7 DAF, 12 DAF and 17 DAF libraries, respectively), which was a greater number compared to the identified stage-specific miRNAs (Table S2E). These observations suggest that it is possible that differences in expression profiles, rather than in the composition of miRNAs, represent the major regulatory mechanisms acting during the grain filling process.

Because of the rapid advances in deep sequencing technology, an increasing number of novel miRNAs are being continuously discovered from various developmental stages of rice. In this study, 60 novel miRNAs were identified in developing rice grain from four individual stages (Table S3). Most of the novel miRNAs discovered in previous studies was found to be present in our dataset. Among the 39 miRNAs reported by Zhu et al. [17] in a 1–10 DAF rice grain library and an additional 26 miRNAs identified in a 3–12 DAF rice grain library [19], only three (osa-miR2103, osa-miR2105 and osa-miR1846-5p) could not be detected in our libraries, and osa-miR1861b/f/i/l, osa-miR1862, osa-miR1867 and osa-miR1874-3p were found to show relatively abundant expression levels. Surprisingly, none of the 11 novel miRNAs reported recently by Lan et al. [21] could be detected in our dataset, which could be due to the different rice subspecies or sampling times we employed.

### Dynamic Expression Patterns of miRNAs during Rice Grains Development

Rice grain filling is a complicated and dynamic process. During this period, dynamic miRNA expression patterns are also observed. By comparing the differential expression pattern of miRNAs between 1–5 DAF and 6–10 DAF grain samples, Zhu et al. [17] found that the expression levels of most miRNAs were either approximately the same between the two libraries or were higher in 6–10 DAF grains than in 1–5 DAF grains. Our result revealed that most of the known miRNAs were expressed constitutively during grain filling, and only 161 known miRNAs were significantly differentially expressed among the four examined libraries, over a half (94) of which were up-regulated significantly from 5 DAF to 7 DAF (Table 2), which was consistent with Zhu’s results. Osa-miR408, which showed 18-fold higher expression in 6–10 DAF grains, was also found to be expressed in the present study, exhibiting 40-fold greater expression in 7 DAF grain than 5 DAF grain, which might more accurately reflect the actual miRNA expression pattern and indicate that the regulation of miRNAs became more active shortly after the initiation of grain filling. Previous studies have shown that developing seeds enter the seed filling stage and that the accumulation of major storage reserves begins at 5 DAF [6], [7], and the expression of genes encoding storage proteins, starch synthesis enzymes and transcriptional factors is enhanced strongly as seed development proceeds from 4 DAF to 6 DAF [41]. The up-regulation of most miRNAs from 5 DAF to 7 DAF is coincident with the early development of the rice endosperm, which may be associated with the onset of the accumulation of storage reserves. More miRNAs were observed to be up-regulated than down-regulated from 7 DAF to 12 DAF (Table 2), too. This pattern changes from 12 DAF-17 DAF, when the fresh weight of the seeds peaks, and they enter the desiccation phase, during which seed metabolism switches to senescence and dormancy, consistent with the observation that more miRNAs were down-regulated than up-regulated from 12 DAF to 17 DAF in this study.

**Table 2:** Expression data of differentially expressed known miRNAs during grain filling.

Transition of developmental stages	Numbers of kn-miRNA with significant differential expression profile	Numbers of up-regulatedkn-miRNA	Numbers of down-regulatedkn-miRNA
5DAF-7DAF	107	94	13
7DAF-12DAF	67	48	19
12DAF-17DAF	47	14	33

Among the 161 differentially expressed miRNAs, some miRNAs were up- or down-regulated gradually during grain filling, which may be associated with the continuous accumulation of storage compounds in rice grain. Some miRNAs showed stage-specific expression patterns during grain filling. For example, the miRNAs in cluster 2 showed an expression pattern that was enriched at 17 DAF, which could be associated with seed maturation and dehydration processes. The miRNAs of cluster 16 and 17 showed 7 DAF- and 12 DAF-enriched expression patterns, respectively, which may indicate that these are sets of stage-specific miRNAs, and their expression profiles might be closely correlated with molecular events that occur specifically at each developmental stage (Fig. 3A).

According to previous a report, the conserved miRNAs are mostly down-regulated, whereas rice-specific miRNAs are mostly up-regulated during grain filling [21]. In this study, non-conserved miRNAs (102 out of 161) represent the majority of differentially expressed miRNAs, including both up- and down-regulated miRNAs (Table S4). This could be due to our use of subspecies japonica, whereas the previous work employed the indica cultivar, or because of the difference in sampling times. These non-conserved miRNAs play a role in establishing and maintaining phenotypic diversity between different groups of organisms from the perspective of evolution and might be responsible for the regulation of species-specific pathways and functions [42]. The differential expression of most non-conserved miRNAs observed in this study indicates different and more important roles for them in regulating gene expression during grain filling.

In this study, all of the novel miRNAs detected showed low abundance, and most of them could not be detected in the 5 DAF library (Table S3), which is consistent with the low expression level of known miRNAs in this library. It is possible that the 5 DAF stage represents an early developmental status for grain filling, and relatively less miRNA is required for regulation at this time. Unlike the constitutive expression pattern of the majority of known miRNAs, nearly all of the novel miRNAs were differentially expressed among the four libraries, indicating that the same regulatory mechanism may be shared between the novel miRNAs and non-conserved known miRNAs.

### Roles of miRNAs in Rice Grain Development

Rice grain filling is a complex process, including a series of highly coordinated cellular events associated with unique transcriptomic profiles. A recent study showed that transcription factors play a key role in the complicated network of transcriptional regulation involved in rice seed development [3]. Many of the predicted target genes of the differentially expressed miRNAs observed in the present study encode transcription factors, suggesting that miRNAs function as master regulators during grain development by regulating the expression of transcription factors (Table S7).

osa-miR159f and Osa-5 are predicted to target an MYB family transcription factor. This transcription factor is a positive gibberellin (GA) signaling component that induces α-amylase expression in rice aleurone cells [43], and is responsible for regulating rice seed maturation and the expression of genes encoding the important storage protein glutelin [44]. MADS box transcripts regulated by osa-miR444 are considered to play an important role in rice ovule and seed development, while growth regulating factor protein (GRF), the target of osa-miR396, functions in ovule initiation and controls seed oil production in *Arabidopsis* [3], [45]. Osa-156l and Osa-29, whose target mRNAs encode Squamosa promoter binding protein-like (SPL) transcription factors, are required for early anther development in *Arabidopsis* [46] and promote cell division and a grain filling rate resulting in higher grain productivity in rice [47], [48].

Phytohormones are important for plant development. Some of the targets of miRNAs are likely to be involved in hormone signal transduction. START domain-containing protein genes regulated by osa-miR166 have been predicted to mediate the transport and signaling of lipids/sterols in plant [49]. Rice fruit development is induced by increased auxin levels in the ovary following pollination [4]. In this study, particularly low expression of osa-miR160 was found to lead to the accumulation of auxin response factors (ARFs), which act as transcriptional activators and repressors that bind to auxin response element to regulate the expression of other genes [50].

The present study also provides some evidence that miRNAs are involved in carbohydrate and nitrogen metabolism. osa-miR1861, whose target mRNAs encode a starch-binding domain-containing protein (*Os01g63810*), are involved in starch degradation [51]. Osa-53 has been found to target a gene encoding sucrose synthase (*Os02g58480*), a key enzyme for converting sucrose into uridine diphosphoglucose (UDPG) and fructose, which is the first step in starch synthesis [52]. The target of Osa-56 encodes a vegetative storage protein (*Os03g42650*), which functions to provide temporary storage of amino acids that can buffer the availability of nitrogen and other nutrients in soybeans [53].It is also worth mentioning that the non-conserved miRNA, osa-miR820, which showed high constitutive expression levels throughout grain filling, has been confirmed to target mRNAs encoding a DNA cytosine methyltransferase (Zmet3-like) (*Os03g02010*) [16] (Table S6), a key enzyme involved in DNA methylation, indicating that epigenetic regulation may be an important mechanism underlying seed development. One target of osa-miR1861h/j has also been predicted to be a methyltransferase (*Os01g03090*) (Table S7). In this study, up-regulation of osa-miR1861h/j during grain filling was observed, together with a higher abundance of 24 nt-long miRNAs in the 5 DAF library, suggesting that miRNA-regulated DNA methylation occurred more frequently in the early stage of grain filling. Further studies are required to identify the mechanism of underlying epigenetic regulation mediated by miRNAs in this process.

### miRNA Base Modifications

A few previous studies have demonstrated that during the miRNA editing events in humans and mice, the dominant editing type is adenosine to inosine (A to I) editing, which can alter base pairing specificity to influence miRNA processing [54]–[56], as well as change the secondary structure of pre-miRNAs or the target selection of mature miRNAs when it occurs within the miRNA seed sequence [57]. Compared to the research progress that has been regarding miRNA editing in mammalian species, studies of miRNA editing in plants are rare. However, recent advances in deep sequencing technology have made it possible to obtain large datasets and to reveal miRNA editing in plants. For example, studies in *Oryza sativa* and *Arabidopsis thaliana* have revealed that cytosine to uracil (C to U) editing in miRNAs is catalyzed by cytosine deaminaes (CDARs), which is similar to events that occur in the RNA of plant mitochondria and other plant organelles [35].

The present sequencing results revealed a large number of miRNA editing events in developing grain, three of which were experimentally validated to occur in vivo (Fig. 5C and Table S10). It is worth mentioning that for one specific type of miRNA editing, the average editing rate at a particular position was similar among the four libraries, and did not differ significantly (one-way ANOVA, p>0.05) (Table S10). For miRNAs showing significantly differential expression, such as osa-miR168a, osa-444f, osa-166m and osa-166j, the miRNA editing rate was similar among the four samples (one-way ANOVA, p>0.05), indicating a fixed editing frequency in each library, regardless of the differences in miRNA expression.

It has been shown that 5′ terminal nucleotides are critical for directing miRNA sorting to Argonaute (AGO) complexes in *Arabidopsis*, and changes in these nucleotides predictably redirect miRNAs into different AGO complexes, altering their biological activity [58]. In the present study, editing events were found to occur at numerous positions, but mainly at positions 2, 3 and 14, and not at the initial position at the 5′-end (Fig. 5B), indicating a strict mechanism to ensure the conservation and stability of 5′ terminal nucleotides of miRNAs. This finding provides further evidence demonstrating the importance of the 5′ terminal nucleotides of miRNAs for their biological functions in plants.

### Conclusion

This study revealed the dynamic characteristics of miRNAs between individual developmental stages during the rice grain filling process, which may be helpful for studying the differential expression of miRNAs during grain development more precisely. Additionally, the sequencing results obtained here enriched the rice miRNA repertoire. GO analysis of miRNAs showing differential expression patterns revealed that miRNAs and their targets may be involved in diverse developmental processes. miRNA editing events were also observed and analyzed. In conclusion, this study revealed a complex regulatory network of miRNAs during rice grain development.

## Supporting Information

Figure S1 **Distribution of sRNAs along miRNA precursors.** A. The corresponding miRNA*(in blue) is more abundant than the annotated miRNAs (in red). B. Examples where the most abundant small RNA is not the annotated miRNA (in red), but one of its variants (in pink) (the sequence in green is the corresponding miRNA* of the miRNA variant). C. Examples where the most abundant small RNA (highlighted in yellow) is not the annotated miRNA, miRNA* or one of their variants (the annotated miRNA and its miRNA* are shown in red and blue, and the variant and its miRNA* are shown in pink and green). D. Examples where a single miRNA precursor produces distinct miRNAs (each miRNA is shown in a different color). **E**. Examples where different miRNA precursors generate the same miRNAs (in red).
(PDF)

Figure S2 **The complete set of clusters of differentially expressed known miRNAs based on K-means support.** The four points from left to right on the x-axis represent the 5 DAF, 7 DAF, 12 DAF and 17 DAF stages, respectively; the y-axis corresponds to the log2 value of the TPM.
(PDF)

Figure S3 **Graphical results of GO analysis of the targets of the differentially expressed known miRNAs.** Targets with GO terms corresponding to (A) biological processes, (B) molecular functions and (C) cellular components. The GO terms and their serial numbers are represented as boxes. For each significant term, the box includes the GO term, adjusted *p-*value (in parentheses), item number mapping the GO term in the query list and background, and the total numbers of items in the query list and background. The color scale indicates the *p-*value cutoff levels for each GO term, where a higher statistical significance corresponds to a darker and redder the color.
(PDF)

Table S1 **Primers used in this study.**
(PDF)

Table S2 **Known miRNAs identified in each library (A-D) and all libraries (E).**
(XLSX)

Table S3 **All predicted novel miRNAs.**
(XLSX)

Table S4 **Known miRNAs showing differential expression pattern among the four libraries.**
(XLSX)

Table S5 **Expression profiles of miRNAs and their targets obtained via real-time quantitative RT-PCR.**
(XLSX)

Table S6 **Predicted targets of known miRNAs.**
(XLSX)

Table S7 **Predicted targets of differentially expressed known miRNAs.**
(XLSX)

Table S8 **Predicted targets of novel miRNAs.**
(XLSX)

Table S9 **Significant GO terms for differentially expressed known miRNA targets.**
(XLSX)

Table S10 **miRNA editing events detected in each library.**
(XLSX)
