# Supplementary material for: Identification and Expression Analysis of microRNAs at the Grain Filling Stage in Rice(Oryza sativa L.)via Deep Sequencing
Source: PLoS One. 2013 Mar 1;8(3):e57863. doi: 10.1371/journal.pone.0057863 (PMC3585941; doi:10.1371/journal.pone.0057863)
Supplement: Figure S1 — Distribution of sRNAs along miRNA precursors. A. The corresponding miRNA*(in blue) is more abundant than the annotated miRNAs (in red). B. Examples where the most abundant small RNA is not the annotated miRNA (in red), but one of its variants (in pink) (the sequence in green is the corresponding miRNA* of the miRNA variant). C. Examples where the most abundant small RNA (highlighted in yellow) is not the annotated miRNA, miRNA* or one of their variants (the annotated miRNA and its miRNA* are shown in red and blue, and the variant and its miRNA* are shown in pink and green). D. Examples where a single miRNA precursor produces distinct miRNAs (each miRNA is shown in a different color). E. Examples where different miRNA precursors generate the same miRNAs (in red). (PDF) [file pone.0057863.s001.pdf]

Figure S1. Distribution of sRNAs along miRNA precursors.

A. The corresponding miRNA*(in blue) is more abundant than the annotated miRNAs (in red)

>osa-MIR1425

```
CTGTTGACTGCATTAGGATTCAATCCTTGCTGCTAAATGTATTGCTTATATTCAGCAATATAATGTTCAGCAGCAAGAACTGGATCTTAATATAGTCGATAG
((((((((((.(((((((((((...((((((((((.((((((((((.......)))))))))...)).)))))))))...))))))))))).))))))))))
............TTAGGATTCAATCCTTGCTGC..................................................................... 14
............TTAGGATTCAATCCTTGCTGCT.................................................................... 1
.............TAGGATTCAATCCTTGCTGCT.................................................................... osa-miR1425 124
.............TAGGATTCAATCCTTGCTGCTAA.................................................................. 20
.............TAGGATTCAATCCTTGCTGCTA................................................................... 11
.............TAGGATTCAATCCTTGCTGC..................................................................... 8
.............TAGGATTCAATCCTTGCTGCTAAA................................................................. 6
.............TAGGATTCAATCCTTGCT....................................................................... 5
.............TAGGATTCAATCCTTGCTG...................................................................... 4
..............AGGATTCAATCCTTGCTGCT.................................................................... 1
..............AGGATTCAATCCTTGCTGCTAAAT................................................................ 1
.................................................................TTCAGCAGCAAGAACTGGATC................ 7
..................................................................TCAGCAGCAAGAACTGGATCT............... 40
..................................................................TCAGCAGCAAGAACTGGATCTTA............. 1
..................................................................TCAGCAGCAAGAACTGGATC................ 1
...................................................................CAGCAGCAAGAACTGGATCTTAAT........... 17
...................................................................CAGCAGCAAGAACTGGATCTTAA............ 2
...................................................................CAGCAGCAAGAACTGGATCT............... 1
....................................................................AGCAGCAAGAACTGGATCTTAATA.......... 4
....................................................................AGCAGCAAGAACTGGATCTTAAT........... 1
.....................................................................GCAGCAAGAACTGGATCTTAA............ 15
.....................................................................GCAGCAAGAACTGGATCTTAAT........... 6
......................................................................CAGCAAGAACTGGATCTTAAT........... osa-miR1425* 1464
......................................................................CAGCAAGAACTGGATCTTAA............ 9
......................................................................CAGCAAGAACTGGATCTTAATA.......... 3
.......................................................................AGCAAGAACTGGATCTTAATA.......... 166
.......................................................................AGCAAGAACTGGATCTTAAT........... 18
.......................................................................AGCAAGAACTGGATCTTAA............ 2
```

>osa-MIR160a

```
GTGTGCCTGGCTCCCTGTATGCCACACATGTAGACCAACCCATGGTGTCTGGTTGCCTACTGGGTGGCGTGCAAGGAGCCAAGCATGC
((((((.(((((((.((((((((((.((.((((..(((((..........))))).)))))).)))))))))).))))))).))))))
...TGCCTGGCTCCCTGTATGCCA................................................................ osa-miR160a 5
...TGCCTGGCTCCCTGTATGC.................................................................. 1
..................................................................GCGTGCAAGGAGCCAAGCATG. osa-miR160a* 122
..................................................................GCGTGCAAGGAGCCAAGCA... 1
```

>osa-MIR398b

```
GGAGTTCCTACAGGGGCGAGCTGGGAACACACGGTGATGAGGCGGTCTGGTCTTTCGTGTGTTCTCAGGTCGCCCCTGCCGGGACTCT
((((((((..(((((((((.((((((((((((((.(((.((.....)).)))..)))))))))))))).)))))))))..))))))))
............GGGGCGAGCTGGGAACACACGG...................................................... 14
............GGGGCGAGCTGGGAACACACG....................................................... 11
............GGGGCGAGCTGGGAACACACGGT..................................................... 3
............GGGGCGAGCTGGGAACACACGGTGA................................................... 1
.............GGGCGAGCTGGGAACACACGG...................................................... osa-miR398b* 181
.............GGGCGAGCTGGGAACACACGGTGA................................................... 2
.............GGGCGAGCTGGGAACACACGGT..................................................... 1
..............GGCGAGCTGGGAACACACGGT..................................................... 65
..............GGCGAGCTGGGAACACACGG...................................................... 10
..............GGCGAGCTGGGAACACACGGTGA................................................... 4
..............GGCGAGCTGGGAACACACGGTG.................................................... 1
...............GCGAGCTGGGAACACACGGTG.................................................... 20
...............GCGAGCTGGGAACACACGGT..................................................... 3
...............GCGAGCTGGGAACACACGGTGAT.................................................. 2
...............GCGAGCTGGGAACACACGG...................................................... 1
................CGAGCTGGGAACACACGGTGA................................................... 3
................CGAGCTGGGAACACACGGTG.................................................... 2
................CGAGCTGGGAACACACGGTGAT.................................................. 2
................CGAGCTGGGAACACACGGT..................................................... 1
................CGAGCTGGGAACACACGGTGATG................................................. 1
......................................................TCGTGTGTTCTCAGGTCGCCC............. 1
.........................................................TGTGTTCTCAGGTCGCCCCTG.......... osa-miR398b 1
```

>osa-MIR408

```
GGGAGTTCTGTGATTGGAGAGGAGAGGAGACAGGGATGAGGCAGAGCATGGGATGGGGCTATCAACAGATGTAGATTATTCCTTGCACAAGAGATGATGATGAGCTGTGAATGAGTTCTGAGAGATGGCTGGTGTTGTTGTTGCTCCCTCCCCTGCACTGCCTCTTCCCTGGCTCCCCTGCACACCTCTCTCTCTCTCTCTCTCTCTGTGT
((((....(.((..((((((((((((...((((((.(((((((.(((.((((.(((((...((((((......(((((..(((....)))..))))).((.((((((...(.(....).)...)))))).)).))))))..))))).)))).))).))))))).)))))((......))...)))))).)))))).)).).))))).....
......................AGAGGAGACAGGGATGAGGCA........................................................................................................................................................................ 25
........................AGGAGACAGGGATGAGGCAGAGCA................................................................................................................................................................... 1
.........................GGAGACAGGGATGAGGCAGAGCA................................................................................................................................................................... 1
..........................GAGACAGGGATGAGGCAGAGCATG................................................................................................................................................................. 4
..........................GAGACAGGGATGAGGCAGAGC.................................................................................................................................................................... 2
..........................GAGACAGGGATGAGGCAGAGCA................................................................................................................................................................... 1
...........................AGACAGGGATGAGGCAGAGCATGG................................................................................................................................................................ 45
...........................AGACAGGGATGAGGCAGAGCA................................................................................................................................................................... 41
...........................AGACAGGGATGAGGCAGAGCATG................................................................................................................................................................. 9
...........................AGACAGGGATGAGGCAGAGC.................................................................................................................................................................... 3
...........................AGACAGGGATGAGGCAGA...................................................................................................................................................................... 1
...........................AGACAGGGATGAGGCAGAGCAT.................................................................................................................................................................. 1
```

```
............................GACAGGGATGAGGCAGAGCAT................................................................................................................................................................... 193
............................GACAGGGATGAGGCAGAGCATG.................................................................................................................................................................. 20
............................GACAGGGATGAGGCAGAGCATGGGA............................................................................................................................................................... 2
............................GACAGGGATGAGGCAGAGCATGG................................................................................................................................................................. 1
............................GACAGGGATGAGGCAGAGCA.................................................................................................................................................................... 1
............................GACAGGGATGAGGCAGAGCATGGG................................................................................................................................................................ 1
.............................ACAGGGATGAGGCAGAGCATG.................................................................................................................................................................. 409
.............................ACAGGGATGAGGCAGAGCAT................................................................................................................................................................... 19
.............................ACAGGGATGAGGCAGAGCATGG................................................................................................................................................................. 14
..............................CAGGGATGAGGCAGAGCATGG................................................................................................................................................................. osa-miR408* 827
..............................CAGGGATGAGGCAGAGCATG.................................................................................................................................................................. 221
..............................CAGGGATGAGGCAGAGCATGGG................................................................................................................................................................ 24
..............................CAGGGATGAGGCAGAGCAT................................................................................................................................................................... 12
..............................CAGGGATGAGGCAGAGCA.................................................................................................................................................................... 2
..............................CAGGGATGAGGCAGAGCATGGGA............................................................................................................................................................... 1
...............................AGGGATGAGGCAGAGCATGGG................................................................................................................................................................ 220
...............................AGGGATGAGGCAGAGCATG.................................................................................................................................................................. 8
...............................AGGGATGAGGCAGAGCATGG................................................................................................................................................................. 6
...............................AGGGATGAGGCAGAGCATGGGA............................................................................................................................................................... 3
................................GGGATGAGGCAGAGCATGGGA............................................................................................................................................................... 16
................................GGGATGAGGCAGAGCATGG................................................................................................................................................................. 1
.................................GGATGAGGCAGAGCATGGGAT.............................................................................................................................................................. 7
.......................................GGCAGAGCATGGGATGGGGCT........................................................................................................................................................ 7
.......................................GGCAGAGCATGGGATGGGGCTATC..................................................................................................................................................... 2
.......................................GGCAGAGCATGGGATGGG........................................................................................................................................................... 1
............................................................................................................................................TTGCTCCCTCCCCTGCACTGC................................................... 1
...............................................................................................................................................CTCCCTCCCCTGCACTGCCTC................................................ 1
........................................................................................................................................................CTGCACTGCCTCTTCCCTGGC....................................... osa-miR408 95
........................................................................................................................................................CTGCACTGCCTCTTCCCTG......................................... 1
.........................................................................................................................................................TGCACTGCCTCTTCCCTGGC....................................... 4
.........................................................................................................................................................TGCACTGCCTCTTCCCTGGCT...................................... 3
.........................................................................................................................................................TGCACTGCCTCTTCCCTGGCTCCC................................... 1
..........................................................................................................................................................GCACTGCCTCTTCCCTGGC....................................... 1
........................................................................................................................................................................................CTCTCTCTCTCTCTCTCTC......... 1
.........................................................................................................................................................................................TCTCTCTCTCTCTCTCTCTCT...... 1
..........................................................................................................................................................................................CTCTCTCTCTCTCTCTCTC....... 1
............................................................................................................................................................................................CTCTCTCTCTCTCTCTCTC..... 1
```

B. Examples where the most abundant small RNA is not the annotated miRNA (in red), but one of its variants (in pink) (the sequence in green is the corresponding miRNA* of the miRNA variant)

>osa-MIR156l

```
GCTAGGGAGCCGACAGAAGAGAGTGAGCATATATAGTTCTTTCCTTGCATATGTGGTCATATGTGTGTTGACTGAAGAGATACATATATATAGAGAGAGAGAGTTCATGTGCTTGAAGCTATATGTGCTCACTTCTCTTTCTGTCAGCAAATTATC
........((.((((((((((((((((((((((((((((...(((...((((((.(.((((((((((((.........))))))))))..........)).).)))))))..))))))))))))))))))).)))).))))))).))........
.......AGCCGACAGAAGAGAGTGAGCATA............................................................................................................................. 1
.........CCGACAGAAGAGAGTGAGCAT.............................................................................................................................. 1
..........CGACAGAAGAGAGTGAGCAT.............................................................................................................................. osa-miR156l variant 1038
..........CGACAGAAGAGAGTGAGCATA............................................................................................................................. osa-miR156l 13
..........CGACAGAAGAGAGTGAGCA............................................................................................................................... 4
..........CGACAGAAGAGAGTGAGC................................................................................................................................ 1
...........GACAGAAGAGAGTGAGCAT.............................................................................................................................. 3
...........GACAGAAGAGAGTGAGCA............................................................................................................................... 2
.................................................................TGTTGACTGAAGAGATACATATAT................................................................... 1
...................................................................TTGACTGAAGAGATACATATATAT................................................................. 1
..............................................................................................................................GCTCACTTCTCTTTCTGTCAGC........ 2
..............................................................................................................................GCTCACTTCTCTTTCTGTCA.......... osa-miR156l variant* 1
```

>osa-MIR167f

```
CACAAGTGGATGAAGCTGCCAGCATGATCTGATCACAGTAGTTCTCTAGCTGATGATGATTTACAAAACCTAGAGACATGCATCAGATCATCTGGCAGTTTCATCTTCTCATG
((..((.((((((((((((((.((((((((((....(((..(((((((.(..((..........))..).)))))))..))))))))))))))))))))))))))).))..))
.........ATGAAGCTGCCAGCATGATCTG.................................................................................. 3
..........TGAAGCTGCCAGCATGATCTGA................................................................................. osa-miR167f variant 50021
..........TGAAGCTGCCAGCATGATCTG.................................................................................. osa-miR167f 595
..........TGAAGCTGCCAGCATGATCT................................................................................... 526
..........TGAAGCTGCCAGCATGATC.................................................................................... 181
..........TGAAGCTGCCAGCATGATCTGAT................................................................................ 74
..........TGAAGCTGCCAGCATGAT..................................................................................... 33
...........GAAGCTGCCAGCATGATCTGA................................................................................. 38
...........GAAGCTGCCAGCATGATC.................................................................................... 1
............AAGCTGCCAGCATGATCTGA................................................................................. 2
............AAGCTGCCAGCATGATCTGATC............................................................................... 1
..................................................................................TCAGATCATCTGGCAGTTT............ 1
..................................................................................TCAGATCATCTGGCAGTTTCA.......... 1
...................................................................................CAGATCATCTGGCAGTTTCA.......... 1
...................................................................................CAGATCATCTGGCAGTTTCATC........ 1
....................................................................................AGATCATCTGGCAGTTTCATC........ osa-miR167f variant* 44
....................................................................................AGATCATCTGGCAGTTTCAT......... 2
```

>osa-MIR1862a

```
GTACTCCCTCCGTCCCGAAATAACCAAACCTCGTAGGACATGTCACATCCTAGTACGAGGTTGGTTTATTTTGGGACGGAGGGAGTAT
(((((((((((((((((((((((((((.((((((((((.........))))...)))))))))).)))))))))))))))))))))))
...................................................AGTACGAGGTTGGTTTATTTTGGG............. 10
...................................................AGTACGAGGTTGGTTTATTTT................ 3
....................................................GTACGAGGTTGGTTTATTTTGGGA............ 59
....................................................GTACGAGGTTGGTTTATTTT................ 5
....................................................GTACGAGGTTGGTTTATTT................. 1
.....................................................TACGAGGTTGGTTTATTTTGGGAC........... 171
.....................................................TACGAGGTTGGTTTATTTT................ 17
```

....TACGAGGTTGGTTTATTTTG............... 6
....TACGAGGTTGGTTTATTT................. 6
....TACGAGGTTGGTTTATTTTGGGA............ 3
....TACGAGGTTGGTTTATTTTGGG............. 3
....TACGAGGTTGGTTTATTTTGG.............. 1
....ACGAGGTTGGTTTATTTT................ osa-miR1862a vatiant 2259
....ACGAGGTTGGTTTATTTTG............... 277
....ACGAGGTTGGTTTATTTTGG.............. 95
....ACGAGGTTGGTTTATTTTGGGA............ 62
....ACGAGGTTGGTTTATTTTGGG............. 40
....ACGAGGTTGGTTTATTTTGGGAC........... 22
....ACGAGGTTGGTTTATTTTGGGACG.......... osa-miR1862a 16
....CGAGGTTGGTTTATTTTGGGACGG......... 16
....CGAGGTTGGTTTATTTTGGGAC........... 7
....CGAGGTTGGTTTATTTTG............... 2
....GAGGTTGGTTTATTTTGGGACGGA........ 6
....GAGGTTGGTTTATTTTGG.............. 3
....AGGTTGGTTTATTTTGGGACGGAG....... 6
....GTTGGTTTATTTTGGGACGG......... 1
....GTTGGTTTATTTTGGGACGGAGG...... 1
....TTGGTTTATTTTGGGACGGAG....... 175
....TTGGTTTATTTTGGGACGGAGGG..... 1
....TTGGTTTATTTTGGGACGG......... 1
....TGGTTTATTTTGGGACGGAG....... 4
....GGTTTATTTTGGGACGGAGGGAGT.. 2
....TTTATTTTGGGACGGAGGGAGTAT 3
....TTTATTTTGGGACGGAGGGAGTA. 1
....TTTATTTTGGGACGGAGG...... 1
....TTATTTTGGGACGGAGGGAGT.. 3
....TTATTTTGGGACGGAGGGAGTA. 2
....TATTTTGGGACGGAGGGAGTA. 4
....TATTTTGGGACGGAGGGAGT.. 3
....TATTTTGGGACGGAGGGA.... 1
....TTTGGGACGGAGGGAGTA. 1

>osa-MIR397b

AGGGAAGGCATTATTGAGTGCAGCGTTGATGAACCTGCCGGCCGGCTAAATTAATTAGCAAGAAAGTCTGAAACTGGCTCAAAGGTTCACCAGCACTGCACCCAATCACGCCTTTGCT
((.((((((...((((.((((((.((((.(((((((...((((.(((((.....))))).((.....))......))))...))))))).)))).)))))).))))...)))))).))
..........TTATTGAGTGCAGCGTTGATG..osa-miR397b 0
............ATTGAGTGCAGCGTTGATGAA... 24
............ATTGAGTGCAGCGTTGATGAACCT.. 2
.............TTGAGTGCAGCGTTGATGAACC... osa-miR397b variant 813
.............TTGAGTGCAGCGTTGATGAAC.. 453
.............TTGAGTGCAGCGTTGATGA.. 114
.............TTGAGTGCAGCGTTGATGAA... 98
.............TTGAGTGCAGCGTTGATG... 46
..............TGAGTGCAGCGTTGATGAACC... 2
..............TGAGTGCAGCGTTGATGAA... 1
...............GAGTGCAGCGTTGATGAAC.. 2
...............GAGTGCAGCGTTGATGAACC... 2
....TTCACCAGCACTGCACCCAATC........... 11
....TCACCAGCACTGCACCCAATC........... osa-miR397b variant* 31
....TCACCAGCACTGCACCCAATCA.......... 1

>osa-MIR820a

TGCGTCGGCCTCGTGGATGGACCAGGAGCTCAACATTCCTTAAGGTTGTTCTTTCAAACCCATACAAGGTTCCACCGCCTGCATTGTTCCAAGAGTGTCTTGGATGAAAGTAGGAAGTGGAACCTTGTTAGGGTTGGAACGAACTGCCTTAAGGAAGTCGATGCTCCAGGTCCGTCCAGGAGGACGACTCA
.(.((((.((((.((((((((((.(((((((.((.(((((((((((.((((.(((.(((((..((((((((((((..(((((.((..((((((.....))))))..)).)))))..))))))))))))..))))).))).)))).))))))))))))).)).))))).)))))))))).)))).)))).).
TGCGTCGGCCTCGTGGATGGA.. 2
TGCGTCGGCCTCGTGGATGGAC... 1
TGCGTCGGCCTCGTGGATGGACCA... 1
..CGTCGGCCTCGTGGATGGACCAGG... 1
....TCGGCCTCGTGGATGGACCAGGAG... osa-miR820a variant 28840
....TCGGCCTCGTGGATGGACCAGGA.. 10756
....TCGGCCTCGTGGATGGACCAGG... 1521
....TCGGCCTCGTGGATGGACCAG.. osa-miR820a 467
....TCGGCCTCGTGGATGGACCA... 33
....TCGGCCTCGTGGATGGAC... 25
....TCGGCCTCGTGGATGGACC.. 24
.....CGGCCTCGTGGATGGACCAGGAG... 80
.....CGGCCTCGTGGATGGACCAGGA.. 46
.....CGGCCTCGTGGATGGACCAGG... 11
.....CGGCCTCGTGGATGGACCAG.. 2
.....CGGCCTCGTGGATGGACCAGGAGC.. 2
......GGCCTCGTGGATGGACCAGGAG... 21
......GGCCTCGTGGATGGACCAGGA.. 16
......GGCCTCGTGGATGGACCAGGAGCT... 2
......GGCCTCGTGGATGGACCAG.. 1
.......GCCTCGTGGATGGACCAGGAG... 50
.......GCCTCGTGGATGGACCAG.. 36
.......GCCTCGTGGATGGACCAGG... 15
.......GCCTCGTGGATGGACCAGGA.. 7
.......GCCTCGTGGATGGACCAGGAGCTC.. 6
.......GCCTCGTGGATGGACCAGGAGC.. 5
........CCTCGTGGATGGACCAGGAG... 1

```
.........CTCGTGGATGGACCAGGAG................................................................................................................................................................... 1
..........TCGTGGATGGACCAGGAG................................................................................................................................................................... 1
............................................................CATACAAGGTTCCACCGCCTGCAT........................................................................................................... 1
........................................................................................................................................AACGAACTGCCTTAGGAAGTCGA................................ 11
........................................................................................................................................AACGAACTGCCTTAGGAAGTCGAT............................... 1
.........................................................................................................................................ACGAACTGCCTTAGGAAGTCGA................................ 13
.........................................................................................................................................ACGAACTGCCTTAGGAAGTCGAT............................... 8
.........................................................................................................................................ACGAACTGCCTTAGGAAGTCGATG.............................. 1
..........................................................................................................................................CGAACTGCCTTAGGAAGTCGAT............................... 2
..........................................................................................................................................CGAACTGCCTTAGGAAGTCGATGC............................. 1
..........................................................................................................................................CGAACTGCCTTAGGAAGTCGATG.............................. 1
...........................................................................................................................................GAACTGCCTTAGGAAGTCGATGCT............................ 24
...........................................................................................................................................GAACTGCCTTAGGAAGTCGATG.............................. 3
...........................................................................................................................................GAACTGCCTTAGGAAGTCGATGC............................. 1
............................................................................................................................................AACTGCCTTAGGAAGTCGATGCT............................ 113
............................................................................................................................................AACTGCCTTAGGAAGTCGATGCTC........................... 33
............................................................................................................................................AACTGCCTTAGGAAGTCGATGC............................. 4
............................................................................................................................................AACTGCCTTAGGAAGTCGATG.............................. 3
.............................................................................................................................................ACTGCCTTAGGAAGTCGATGCTC........................... 8
.............................................................................................................................................ACTGCCTTAGGAAGTCGATGCT............................ 5
.............................................................................................................................................ACTGCCTTAGGAAGTCGATGCTCC.......................... 3
.............................................................................................................................................ACTGCCTTAGGAAGTCGATG.............................. 1
..............................................................................................................................................CTGCCTTAGGAAGTCGATGC............................. 5
..............................................................................................................................................CTGCCTTAGGAAGTCGATGCT............................ 3
...............................................................................................................................................TGCCTTAGGAAGTCGATGCTC........................... 15
...............................................................................................................................................TGCCTTAGGAAGTCGATGCT............................ 11
...............................................................................................................................................TGCCTTAGGAAGTCGATG.............................. 2
...............................................................................................................................................TGCCTTAGGAAGTCGATGC............................. 1
...............................................................................................................................................TGCCTTAGGAAGTCGATGCTCCAG........................ 1
.................................................................................................................................................CCTTAGGAAGTCGATGCT............................ 1
...................................................................................................................................................TTAGGAAGTCGATGCTCCAGGTCC.................... 1
.......................................................................................................................................................GAAGTCGATGCTCCAGGTCCGTCCA............... 3
........................................................................................................................................................AAGTCGATGCTCCAGGTCCGTCCA............... 1
```

C. Examples where the most abundant small RNA (highlighted in yellow) is not the annotated miRNA, miRNA* or one of their variants (the annotated miRNA and its miRNA* are shown in red and blue, and the variant and its miRNA* are shown in pink and green)

>osa-MIR1441

```
GTTTTTTCATTCGTGTCCGAAAACTCCTTTTGATATCTGGTCAAACATTCGATGTGACATCTAAAAATTTTCTTTTCGCGAACTAAGGGCCTGTTTAGTTCGCGAAAAGAAAATTTTTGGGTGTCACATCGGACGTTTAACCGGATGTCGGAAAAGGTTTTCAGACACGAATAAAAAAAC
(((((((.(((((((((.(((((((..((((((((((((((.((((.((((((((((((((((((((((((((((((((((((((((......)))))))))))))))))))))))))))))))))))))))).)))).))))))))))))))..))))))).))))))))).)))))))
...TTTTCATTCGTGTCCGAAA.............................................................................................................................................................. 2
.....TTCATTCGTGTCCGAAAAC............................................................................................................................................................ 1
......................ACTCCTTTTGATATCTGGTCA..........................................................................................................................................osa-miR1441* 0
.......................CTCCTTTTGATATCTGGTCAAAC.......................................................................................................................................osa-miR1441 variant* 0
..............................................................AAAAATTTTCTTTTCGCGAACTAA.............................................................................................. 6
.................................................................AATTTTCTTTTCGCGAACT................................................................................................ 1
..................................................................................................TTCGCGAAAAGAAAATTTTTGGGT.......................................................... 2
.......................................................................................................GAAAAGAAAATTTTTGGGTGTCACA.................................................... 1
.........................................................................................................AAAGAAAATTTTTGGGTGTCACAT................................................... 2
..........................................................................................................AAGAAAATTTTTGGGTGTCACATC.................................................. 2
............................................................................................................GAAAATTTTTGGGTGTCACATCGG................................................ 2
............................................................................................................GAAAATTTTTGGGTGTCACATCGGA............................................... 1
.............................................................................................................AAAATTTTTGGGTGTCACATCGGA............................................... 2
.............................................................................................................AAAATTTTTGGGTGTCACATC.................................................. 1
..............................................................................................................AAATTTTTGGGTGTCACATCGGAC.............................................. 9
..............................................................................................................AAATTTTTGGGTGTCACATCGG................................................ 2
..............................................................................................................AAATTTTTGGGTGTCACATC.................................................. 2
..............................................................................................................AAATTTTTGGGTGTCACAT................................................... 1
..............................................................................................................AAATTTTTGGGTGTCACATCG................................................. 1
...............................................................................................................AATTTTTGGGTGTCACATC.................................................. 7
...............................................................................................................AATTTTTGGGTGTCACATCGGACG............................................. 5
...............................................................................................................AATTTTTGGGTGTCACAT................................................... 1
...............................................................................................................AATTTTTGGGTGTCACATCGGA............................................... 1
...............................................................................................................AATTTTTGGGTGTCACATCGGAC.............................................. 1
................................................................................................................ATTTTTGGGTGTCACATCGGACGT............................................ 7
................................................................................................................ATTTTTGGGTGTCACATCGGACG............................................. 5
................................................................................................................ATTTTTGGGTGTCACATCGGACGTT........................................... 1
.................................................................................................................TTTTTGGGTGTCACATCGGACGTT........................................... 25
.................................................................................................................TTTTTGGGTGTCACATCGGACGTTT.......................................... 3
.................................................................................................................TTTTTGGGTGTCACATCGGACGT............................................ 2
.................................................................................................................TTTTTGGGTGTCACATCGGACG............................................. 1
..................................................................................................................TTTTGGGTGTCACATCGGACGTTT.......................................... 133
..................................................................................................................TTTTGGGTGTCACATCGGACGTT........................................... 20
..................................................................................................................TTTTGGGTGTCACATCGGAC.............................................. 1
..................................................................................................................TTTTGGGTGTCACATCGGACG............................................. 1
...................................................................................................................TTTGGGTGTCACATCGGACGTTT.......................................... 35
...................................................................................................................TTTGGGTGTCACATCGGACGTT........................................... 4
....................................................................................................................TTGGGTGTCACATCGGACGTTTA......................................... 8
....................................................................................................................TTGGGTGTCACATCGGACGTTT.......................................... 2
.....................................................................................................................TGGGTGTCACATCGGACGTTT.......................................... 1
......................................................................................................................GGGTGTCACATCGGACGTTT.......................................... 10
........................................................................................................................GTGTCACATCGGACGTTT.......................................... 1
..........................................................................................................................GTCACATCGGACGTTTAACCGGAT.................................. 2
.............................................................................................................................ACATCGGACGTTTAACCGGAT.................................. 1
.............................................................................................................................ACATCGGACGTTTAACCGGA................................... 1
```

```
...............................................................................................................................ATCGGACGTTTAACCGGATGTCGG............................. 19
................................................................................................................................TCGGACGTTTAACCGGATGTCGGA............................ 7
................................................................................................................................TCGGACGTTTAACCGGATGTCGG............................. 5
................................................................................................................................TCGGACGTTTAACCGGATG................................. 2
................................................................................................................................TCGGACGTTTAACCGGATGT................................ 1
................................................................................................................................TCGGACGTTTAACCGGATGTCG.............................. 1
.................................................................................................................................CGGACGTTTAACCGGATGTCGGAA........................... 30
.................................................................................................................................CGGACGTTTAACCGGATGTCGGA............................ 13
.................................................................................................................................CGGACGTTTAACCGGATGT................................ 1
..................................................................................................................................GGACGTTTAACCGGATGTCGGAA........................... 6
........................................................................................................................................TTAACCGGATGTCGGAAAAGGTT..................... osa-miR1441 variant 3
.........................................................................................................................................TAACCGGATGTCGGAAAAGGTTT.................... 1
..........................................................................................................................................AACCGGATGTCGGAAAAGGTTTTC.................. 1
... ...................................................................................................................................... ACCGGATGTCGGAAAAGGTTT....................osa-miR1441 0
............................................................................................................................................CCGGATGTCGGAAAAGGTTTTCAG................ 2
............................................................................................................................................CCGGATGTCGGAAAAGGTTT.................... 1
..............................................................................................................................................GGATGTCGGAAAAGGTTTT................... 1
```

>osa-MIR809g

```
ACTTCCTCCGTTTCACAATGTAAGTCATTTTAGCATTTCCCATATTCATATTGATGCTAATGAATATATATAAATATATATGTCTAGATTCATTAGCATCAATATGAATGTGAGAAATGTTAGAATGACTTACATTGTGAAACGGAGGGAGT
(((((((((((((((((((((((((((((((((((((((.((((((((((((((((((((((((((((((((....)))))))....))))))))))))))))))))))))).)))))))))))))))))))))))))))))))))))))))
..TTCCTCCGTTTCACAATGTAAGTC.............................................................................................................................. 1
....CCTCCGTTTCACAATGTAAGTCAT............................................................................................................................ 2
.....CTCCGTTTCACAATGTAAG................................................................................................................................ 1
.....CTCCGTTTCACAATGTAAGTCATT........................................................................................................................... 1
.......CCGTTTCACAATGTAAGTCATT........................................................................................................................... 1
.......CCGTTTCACAATGTAAGTCAT............................................................................................................................ 1
.......CCGTTTCACAATGTAAGTCA............................................................................................................................. 1
..........TTTCACAATGTAAGTCATTTT......................................................................................................................... 1
.............CACAATGTAAGTCATTTTAGCA..................................................................................................................... 1
.............CACAATGTAAGTCATTTTAGCATTT.................................................................................................................. 1
................AATGTAAGTCATTTTAGCATT................................................................................................................... 1
....................TAAGTCATTTTAGCATTTCCC............................................................................................................... 2
.....................AAGTCATTTTAGCATTTCCCA.............................................................................................................. 1
.....................AAGTCATTTTAGCATTTCCCATAT........................................................................................................... 1
.........................CATTTTAGCATTTCCCATATTCA........................................................................................................ 1
............................TTTAGCATTTCCCATATTCATA......................................................................................................osa-miR809g* 0
............................TTTAGCATTTCCCATATTCATATT.................................................................................................... osa-miR809g variant* 1
..............................TAGCATTTCCCATATTCATATTGA.................................................................................................. 1
...............................AGCATTTCCCATATTCATATTGAT................................................................................................. 2
...............................AGCATTTCCCATATTCATAT..................................................................................................... 1
..........................................TATTCATATTGATGCTAATG.......................................................................................... 1
.................................................ATTGATGCTAATGAATATATATAA............................................................................... 1
...........................................................................ATATATGTCTAGATTCATTA......................................................... 1
...............................................................................ATGTCTAGATTCATTAGCATCAAT................................................. 1
....................................................................................TAGATTCATTAGCATCAATATGAA............................................ 1
.....................................................................................AGATTCATTAGCATCAATATGAAT........................................... 4
.......................................................................................ATTCATTAGCATCAATATGA............................................. 1
........................................................................................TTCATTAGCATCAATATGAAT........................................... 1
...........................................................................................ATTAGCATCAATATGAATG.......................................... 1
............................................................................................TTAGCATCAATATGAATG.......................................... 3
..................................................................... ..................................TGAATGTGAGAAATGTTAGAAT.......................... osa-miR809g 0
...........................................................................................................ATGTGAGAAATGTTAGAATGACTT..................... 1
...........................................................................................................ATGTGAGAAATGTTAGAATGACT...................... 1
............................................................................................................TGTGAGAAATGTTAGAATGACTTA.................... osa-miR809g variant 2
.............................................................................................................GTGAGAAATGTTAGAATGACTTA.................... 1
..............................................................................................................TGAGAAATGTTAGAATGACTTACA.................. 1
..............................................................................................................TGAGAAATGTTAGAATGACTTA.................... 1
.................................................................................................................GAAATGTTAGAATGACTTACATT................ 1
..................................................................................................................AAATGTTAGAATGACTTACATT................ 3
..................................................................................................................AAATGTTAGAATGACTTACAT................. 2
..................................................................................................................AAATGTTAGAATGACTTACATTGT.............. 2
..................................................................................................................AAATGTTAGAATGACTTAC................... 1
...................................................................................................................AATGTTAGAATGACTTACATTGTG............. 2
...................................................................................................................AATGTTAGAATGACTTACATT................ 1
...................................................................................................................AATGTTAGAATGACTTACAT................. 1
....................................................................................................................ATGTTAGAATGACTTACATTGTGA............ 3
....................................................................................................................ATGTTAGAATGACTTACATTGTG............. 3
.....................................................................................................................TGTTAGAATGACTTACATTGTGAA........... 2
......................................................................................................................GTTAGAATGACTTACATTGTGAAA.......... 2
......................................................................................................................GTTAGAATGACTTACATTGTG............. 1
........................................................................................................................TAGAATGACTTACATTGTGAA........... 28
........................................................................................................................TAGAATGACTTACATTGTGAAACG........ 3
........................................................................................................................TAGAATGACTTACATTGTGAAAC......... 2
........................................................................................................................TAGAATGACTTACATTGTGA............ 1
........................................................................................................................TAGAATGACTTACATTGTG............. 1
.........................................................................................................................AGAATGACTTACATTGTGAAACGG....... 158
.........................................................................................................................AGAATGACTTACATTGTGAAACG........ 24
.........................................................................................................................AGAATGACTTACATTGTGAA........... 1
.........................................................................................................................AGAATGACTTACATTGTGAAA.......... 1
.........................................................................................................................AGAATGACTTACATTGTG............. 1
.........................................................................................................................AGAATGACTTACATTGTGAAAC......... 1
.........................................................................................................................AGAATGACTTACATTGTGAAACGGA...... 1
..........................................................................................................................GAATGACTTACATTGTGAAACGGA...... 80
```

```
..........................................................................................................................GAATGACTTACATTGTGAAACGG....... 8
...........................................................................................................................AATGACTTACATTGTGAAACGGAG..... 42
...........................................................................................................................AATGACTTACATTGTGAAACGGA...... 20
...........................................................................................................................AATGACTTACATTGTGAAACGG....... 4
...........................................................................................................................AATGACTTACATTGTGAAACG........ 1
............................................................................................................................ATGACTTACATTGTGAAACGGAGG.... 22
............................................................................................................................ATGACTTACATTGTGAAACGG....... 17
............................................................................................................................ATGACTTACATTGTGAAACGGA...... 10
............................................................................................................................ATGACTTACATTGTGAAACGGAG..... 7
.............................................................................................................................TGACTTACATTGTGAAACGGA...... 70
.............................................................................................................................TGACTTACATTGTGAAACGGAGG.... 53
.............................................................................................................................TGACTTACATTGTGAAACGGAGGG... 39
.............................................................................................................................TGACTTACATTGTGAAACGGAG..... 12
.............................................................................................................................TGACTTACATTGTGAAACG........ 7
.............................................................................................................................TGACTTACATTGTGAAACGG....... 3
.............................................................................................................................TGACTTACATTGTGAAAC......... 1
..............................................................................................................................GACTTACATTGTGAAACGGAGGGA.. 77
..............................................................................................................................GACTTACATTGTGAAACGGAGG.... 16
..............................................................................................................................GACTTACATTGTGAAACGGAGGG... 15
..............................................................................................................................GACTTACATTGTGAAACGGAG..... 8
..............................................................................................................................GACTTACATTGTGAAACGGA...... 4
..............................................................................................................................GACTTACATTGTGAAACGGAGGGAG. 2
...............................................................................................................................ACTTACATTGTGAAACGGAGG.... 12
...............................................................................................................................ACTTACATTGTGAAACGGAGGGAG. 9
...............................................................................................................................ACTTACATTGTGAAACGGAGGGA.. 4
...............................................................................................................................ACTTACATTGTGAAACGGAGGG... 3
...............................................................................................................................ACTTACATTGTGAAACGGAG..... 2
................................................................................................................................CTTACATTGTGAAACGGAGGGAGT 13
................................................................................................................................CTTACATTGTGAAACGGAGG.... 6
................................................................................................................................CTTACATTGTGAAACGGAGGG... 4
................................................................................................................................CTTACATTGTGAAACGGAG..... 4
................................................................................................................................CTTACATTGTGAAACGGAGGGAG. 2
................................................................................................................................CTTACATTGTGAAACGGA...... 2
.................................................................................................................................TTACATTGTGAAACGGAGG.... 10
.................................................................................................................................TTACATTGTGAAACGGAGGGA.. 3
.................................................................................................................................TTACATTGTGAAACGGAGGG... 2
.................................................................................................................................TTACATTGTGAAACGGAGGGAG. 2
.................................................................................................................................TTACATTGTGAAACGGAGGGAGT 2
.................................................................................................................................TTACATTGTGAAACGGAG..... 1
..................................................................................................................................TACATTGTGAAACGGAGGGAG. 5
..................................................................................................................................TACATTGTGAAACGGAGG.... 3
...................................................................................................................................ACATTGTGAAACGGAGGGAGT 10
...................................................................................................................................ACATTGTGAAACGGAGGG... 7
...................................................................................................................................ACATTGTGAAACGGAGGGA.. 2
....................................................................................................................................CATTGTGAAACGGAGGGAGT 2
```

>osa-MIR819a

```
CCTCCGTTTCAGGTTATAAGACTTTCTAGCATTGCCCACATTCATATAGATGTTAATGAATCTAGGCATATAACATCTATATGAATGTGGGCAATGCTAGAAAGTCTTATAATATGAAACGGAGG
((((((((((.(((((((((((((((((((((((((((((((((((((((((((((((........))).))))))))))))))))))))))))))))))))))))))))))).)))))))))))
CCTCCGTTTCAGGTTATA........................................................................................................... 2
CCTCCGTTTCAGGTTATAAGACTT..................................................................................................... 2
CCTCCGTTTCAGGTTATAAGACT...................................................................................................... 1
.CTCCGTTTCAGGTTATAAGACTTT.................................................................................................... 2
..TCCGTTTCAGGTTATAAGACT...................................................................................................... 1
..TCCGTTTCAGGTTATAAGACTTTC................................................................................................... 1
..TCCGTTTCAGGTTATAAGACTTT.................................................................................................... 1
...CCGTTTCAGGTTATAAGACTTTCT.................................................................................................. 2
.....GTTTCAGGTTATAAGACTTTCTAGCAT............................................................................................. 1
......TTTCAGGTTATAAGACTTTCT.................................................................................................. 1
```
........TCAGGTTATAAGACTTTCTAGC...osa-miR819a 0

........TCAGGTTATAAGACTTTCTAGCAT... osa-miR819a variant 75
```
........TCAGGTTATAAGACTTTCTAGCA.............................................................................................. 5
........TCAGGTTATAAGACTTTCTAGCATT............................................................................................ 2
.........CAGGTTATAAGACTTTCTAGCATT............................................................................................ 43
.........CAGGTTATAAGACTTTCTAGCAT............................................................................................. 1
..........AGGTTATAAGACTTTCTAGCAT............................................................................................. 16
..........AGGTTATAAGACTTTCTAGCATT............................................................................................ 4
..........AGGTTATAAGACTTTCTAGCATTG........................................................................................... 2
..........AGGTTATAAGACTTTCTAGCA.............................................................................................. 2
............GTTATAAGACTTTCTAGCATTGCC......................................................................................... 1
.............TTATAAGACTTTCTAGCATTG........................................................................................... 10
.............TTATAAGACTTTCTAGCATTGCCC........................................................................................ 3
.............TTATAAGACTTTCTAGCATT............................................................................................ 3
.............TTATAAGACTTTCTAGCATTGCC......................................................................................... 1
..............TATAAGACTTTCTAGCATTGCCCA....................................................................................... 5
..............TATAAGACTTTCTAGCATTGC.......................................................................................... 1
...............ATAAGACTTTCTAGCATTGCCCAC...................................................................................... 73
...............ATAAGACTTTCTAGCATTGCCCA....................................................................................... 9
...............ATAAGACTTTCTAGCATTGCCC........................................................................................ 4
...............ATAAGACTTTCTAGCATTGCC......................................................................................... 2
...............ATAAGACTTTCTAGCATTGC.......................................................................................... 1
................TAAGACTTTCTAGCATTGCCCACA..................................................................................... 29
................TAAGACTTTCTAGCATTGCCC........................................................................................ 15
................TAAGACTTTCTAGCATTGC.......................................................................................... 5
```

```
................TAAGACTTTCTAGCATTGCC.......................................................................................... 2
................TAAGACTTTCTAGCATTGCCCA........................................................................................ 2
................TAAGACTTTCTAGCATTGCCCACAT..................................................................................... 1
................TAAGACTTTCTAGCATTG............................................................................................ 1
.................AAGACTTTCTAGCATTGCCCACAT..................................................................................... 23
.................AAGACTTTCTAGCATTGCCCACA...................................................................................... 3
.................AAGACTTTCTAGCATTGCC.......................................................................................... 2
.................AAGACTTTCTAGCATTGCCCA........................................................................................ 1
.................AAGACTTTCTAGCATTGCCC......................................................................................... 1
..................AGACTTTCTAGCATTGCCCAC....................................................................................... 3
..................AGACTTTCTAGCATTGCCCACA...................................................................................... 1
..................AGACTTTCTAGCATTGCCCACATT.................................................................................... 1
...................GACTTTCTAGCATTGCCCACATTCAT................................................................................. 1
....................ACTTTCTAGCATTGCCCACAT..................................................................................... 2
....................ACTTTCTAGCATTGCCCACATTCA.................................................................................. 1
.....................CTTTCTAGCATTGCCCACATTCAT................................................................................. 7
.....................CTTTCTAGCATTGCCCACATT.................................................................................... 1
.......................TTCTAGCATTGCCCACATTCA.................................................................................. 2
.......................TTCTAGCATTGCCCACATTCATAT............................................................................... 1
.......................TTCTAGCATTGCCCACATTCAT................................................................................. 1
........................TCTAGCATTGCCCACATTCATATA.............................................................................. 1
.........................CTAGCATTGCCCACATTCATATAG............................................................................. 1
..........................TAGCATTGCCCACATTCATAT............................................................................... 2
...........................AGCATTGCCCACATTCATATAGAT........................................................................... 4
............................GCATTGCCCACATTCATATAGATG.......................................................................... 3
............................GCATTGCCCACATTCATATAG............................................................................. 1
..............................ATTGCCCACATTCATATAGATGTTA....................................................................... 1
...............................TTGCCCACATTCATATAGAT........................................................................... 1
...............................TTGCCCACATTCATATAGATG.......................................................................... 1
.................................GCCCACATTCATATAGATGT......................................................................... 1
...................................CCACATTCATATAGATGTTAATGAA.................................................................. 1
....................................CACATTCATATAGATGTTAAT..................................................................... 1
.....................................ACATTCATATAGATGTTAATGAAT................................................................. 4
.........................................TCATATAGATGTTAATGA................................................................... 1
..........................................CATATAGATGTTAATGAATCTAGG............................................................ 4
..........................................CATATAGATGTTAATGAATC................................................................ 1
..........................................CATATAGATGTTAATGAATCT............................................................... 1
...........................................ATATAGATGTTAATGAATCTAGGC........................................................... 9
...........................................ATATAGATGTTAATGAATCTAG............................................................. 1
............................................TATAGATGTTAATGAATCTAGGCA.......................................................... 1
.............................................ATAGATGTTAATGAATCTAGGCAT......................................................... 1
.............................................ATAGATGTTAATGAATCTAGGCA.......................................................... 1
..............................................TAGATGTTAATGAATCTAGGCA.......................................................... 1
...............................................AGATGTTAATGAATCTAGGCA.......................................................... 3
...............................................AGATGTTAATGAATCTAGGCATA........................................................ 2
...............................................AGATGTTAATGAATCTAGGCATAT....................................................... 1
.................................................ATGTTAATGAATCTAGGC........................................................... 1
.....................................................................ATAACATCTATATGAATGTGGGCA................................. 1
.......................................................................AACATCTATATGAATGTGGGCAAT............................... 2
.......................................................................AACATCTATATGAATGTGGGC.................................. 1
........................................................................ACATCTATATGAATGTGGGCA................................. 8
........................................................................ACATCTATATGAATGTGGGCAATG.............................. 1
........................................................................ACATCTATATGAATGTGGGCAA................................ 1
.........................................................................CATCTATATGAATGTGGGCAATGC............................. 4
.........................................................................CATCTATATGAATGTGGGCAA................................ 2
.........................................................................CATCTATATGAATGTGGGCA................................. 1
..........................................................................ATCTATATGAATGTGGGCAAT............................... 74
..........................................................................ATCTATATGAATGTGGGCAATGCT............................ 71
..........................................................................ATCTATATGAATGTGGGCAATG.............................. 33
..........................................................................ATCTATATGAATGTGGGCAATGC............................. 5
..........................................................................ATCTATATGAATGTGGGCA................................. 2
..........................................................................ATCTATATGAATGTGGGCAA................................ 2
...........................................................................TCTATATGAATGTGGGCAATG.............................. 253
...........................................................................TCTATATGAATGTGGGCAATGCTA........................... 154
...........................................................................TCTATATGAATGTGGGCAATGC............................. 19
...........................................................................TCTATATGAATGTGGGCAATGCT............................ 7
...........................................................................TCTATATGAATGTGGGCAA................................ 2
...........................................................................TCTATATGAATGTGGGCAAT............................... 1
...........................................................................TCTATATGAATGTGGGCAATGCTAGA......................... 1
...........................................................................TCTATATGAATGTGGGCAATGCTAG.......................... 1
...........................................................................TCTATATGAATGTGGGCA................................. 1
............................................................................CTATATGAATGTGGGCAATGCTAG.......................... 16
............................................................................CTATATGAATGTGGGCAATG.............................. 10
............................................................................CTATATGAATGTGGGCAATGCTA........................... 3
............................................................................CTATATGAATGTGGGCAATGC............................. 3
.............................................................................TATATGAATGTGGGCAATGCTAGA......................... 124
.............................................................................TATATGAATGTGGGCAATGCT............................ 34
.............................................................................TATATGAATGTGGGCAATGCTAG.......................... 13
.............................................................................TATATGAATGTGGGCAATGCTA........................... 7
.............................................................................TATATGAATGTGGGCAATG.............................. 2
.............................................................................TATATGAATGTGGGCAATGC............................. 1
..............................................................................ATATGAATGTGGGCAATGCTAGAA........................ 163
..............................................................................ATATGAATGTGGGCAATGCTAGA......................... 34
..............................................................................ATATGAATGTGGGCAATGCTAG.......................... 19
```

```
.............................................................................ATATGAATGTGGGCAATGCTA.......................... 15
.............................................................................ATATGAATGTGGGCAATGC............................ 1
.............................................................................ATATGAATGTGGGCAATGCT........................... 1
..............................................................................TATGAATGTGGGCAATGCTAGAAA...................... 1976
..............................................................................TATGAATGTGGGCAATGCTAG......................... 100
..............................................................................TATGAATGTGGGCAATGCTAGAA....................... 76
..............................................................................TATGAATGTGGGCAATGCTAGA........................ 15
..............................................................................TATGAATGTGGGCAATGCTAGAAAG..................... 13
..............................................................................TATGAATGTGGGCAATGCTA.......................... 8
..............................................................................TATGAATGTGGGCAATGC............................ 2
..............................................................................TATGAATGTGGGCAATGCT........................... 1
...............................................................................ATGAATGTGGGCAATGCTAGAAAG..................... 320
...............................................................................ATGAATGTGGGCAATGCTAGAAA...................... 40
...............................................................................ATGAATGTGGGCAATGCTAGA........................ 5
...............................................................................ATGAATGTGGGCAATGCTAGAA....................... 3
...............................................................................ATGAATGTGGGCAATGCTAG......................... 3
...............................................................................ATGAATGTGGGCAATGCTAGAAAGT.................... 2
...............................................................................ATGAATGTGGGCAATGCTA.......................... 1
................................................................................TGAATGTGGGCAATGCTAGAAAGT.................... 80
................................................................................TGAATGTGGGCAATGCTAGAAA...................... 55
................................................................................TGAATGTGGGCAATGCTAGAA....................... 52
................................................................................TGAATGTGGGCAATGCTAGAAAG..................... 22
................................................................................TGAATGTGGGCAATGCTA.......................... 1
.................................................................................GAATGTGGGCAATGCTAGAAAGTC................... 11
.................................................................................GAATGTGGGCAATGCTAGAAAGT.................... 1
.................................................................................GAATGTGGGCAATGCTAGAA....................... 1
.................................................................................GAATGTGGGCAATGCTAGAAAG..................... 1
..................................................................................AATGTGGGCAATGCTAGAAAGTC................... 1
...................................................................................ATGTGGGCAATGCTAGAAAGTCT.................. 1
...................................................................................ATGTGGGCAATGCTAGAAAGTCTT................. 1
...................................................................................ATGTGGGCAATGCTAGAAAGT.................... 1
...................................................................................ATGTGGGCAATGCTAGAAAGTC................... 1
....................................................................................TGTGGGCAATGCTAGAAAGTCTTA................ 8
....................................................................................TGTGGGCAATGCTAGAAAGTC................... 7
....................................................................................TGTGGGCAATGCTAGAAAGTCTT................. 3
....................................................................................TGTGGGCAATGCTAGAAAG..................... 2
.....................................................................................GTGGGCAATGCTAGAAAGTCTTA................ 4
.....................................................................................GTGGGCAATGCTAGAAAGTCTTAT............... 3
.....................................................................................GTGGGCAATGCTAGAAAGTCT.................. 2
.....................................................................................GTGGGCAATGCTAGAAAGTCTTATA.............. 1
.....................................................................................GTGGGCAATGCTAGAAAGTCTT................. 1
......................................................................................TGGGCAATGCTAGAAAGTCTT................. 75
......................................................................................TGGGCAATGCTAGAAAGTCTTATA.............. 66
......................................................................................TGGGCAATGCTAGAAAGTCTTA................ 36
......................................................................................TGGGCAATGCTAGAAAGTCTTAT............... 6
......................................................................................TGGGCAATGCTAGAAAGTCT.................. 6
......................................................................................TGGGCAATGCTAGAAAGT.................... 1
......................................................................................TGGGCAATGCTAGAAAGTC................... 1
.......................................................................................GGGCAATGCTAGAAAGTCTTATAA............. 1
.......................................................................................GGGCAATGCTAGAAAGTCTTATA.............. 1
........................................................................................GGCAATGCTAGAAAGTCTTAT............... 1
..........................................................................................CAATGCTAGAAAGTCTTATAATAT.......... 7
..........................................................................................CAATGCTAGAAAGTCTTATAA............. 4
...........................................................................................AATGCTAGAAAGTCTTATAATATG......... 5
...........................................................................................AATGCTAGAAAGTCTTATA.............. 1
...........................................................................................AATGCTAGAAAGTCTTATAA............. 1
...........................................................................................AATGCTAGAAAGTCTTATAAT............ 1
............................................................................................ATGCTAGAAAGTCTTATAATATGA........ 3
............................................................................................ATGCTAGAAAGTCTTATAATA........... 2
................................................................................................TAGAAAGTCTTATAATATGAAA.......osa-miR819a* 0
.................................................................................................AGAAAGTCTTATAATATGAAACGG... osa-miR819a variant* 28
.................................................................................................AGAAAGTCTTATAATATGAAACG.... 1
..................................................................................................GAAAGTCTTATAATATGAAACGGA.. 2
..................................................................................................GAAAGTCTTATAATATGAAACGG... 2
...................................................................................................AAAGTCTTATAATATGAAACGGAG. 8
...................................................................................................AAAGTCTTATAATATGAAACGG... 1
....................................................................................................AAGTCTTATAATATGAAACGGAGG 4
....................................................................................................AAGTCTTATAATATGAAACGGAG. 2
.....................................................................................................AGTCTTATAATATGAAACGGA.. 2
.......................................................................................................TCTTATAATATGAAACGGA.. 1
.......................................................................................................TCTTATAATATGAAACGG... 1
........................................................................................................CTTATAATATGAAACGGA.. 1
```

>osa-MIR2906b

```
GGCTGGTTTCACAGAGCAGCGAATACTGCCCGCTTCCAACGGTGGAAGGATAACGGGCCGCCGCACTGCTGGCCCGCT
(((.((((...(((.((.(((......(((((((((((....)))))).....)))))))).)).)))..)))).)))
........TCACAGAGCAGCGAATACTGCCCG...............................................osa-miR2906b* 0
..........ACAGAGCAGCGAATACTGCCC................................................osa-miR2906b variant* 0
..............................CGCTTCCAACGGTGGAAGGATAAC........................ 1
...............................GCTTCCAACGGTGGAAGGATAACG....................... 6
...............................GCTTCCAACGGTGGAAGGATAACGG...................... 1
................................CTTCCAACGGTGGAAGGATAACGGGC.................... 1
................................CTTCCAACGGTGGAAGGATAACGG...................... 1
.................................TTCCAACGGTGGAAGGATAACG....................... 13
```

```
.................................TTCCAACGGTGGAAGGATAAC........................ 4
.................................TTCCAACGGTGGAAGGATAACGGGC.................... 1
.................................TTCCAACGGTGGAAGGATAACGG...................... 1
..................................TCCAACGGTGGAAGGATAACG....................... 23
..................................TCCAACGGTGGAAGGATAACGG...................... 15
..................................TCCAACGGTGGAAGGATAACGGG..................... 8
..................................TCCAACGGTGGAAGGATAA......................... 4
..................................TCCAACGGTGGAAGGATAACGGGC.................... 3
..................................TCCAACGGTGGAAGGATAACGGGCC................... 1
..................................TCCAACGGTGGAAGGATAACGGGCCG.................. 1
..................................TCCAACGGTGGAAGGATAAC........................ 1
...................................CCAACGGTGGAAGGATAACG....................... 60
...................................CCAACGGTGGAAGGATAACGG...................... 34
...................................CCAACGGTGGAAGGATAACGGG..................... 9
...................................CCAACGGTGGAAGGATAACGGGC.................... 4
...................................CCAACGGTGGAAGGATAAC........................ 4
...................................CCAACGGTGGAAGGATAACGGGCCG.................. 1
...................................CCAACGGTGGAAGGATAA......................... 1
...................................CCAACGGTGGAAGGATAACGGGCC................... 1
....................................CAACGGTGGAAGGATAACG....................... 69
....................................CAACGGTGGAAGGATAACGG...................... 24
....................................CAACGGTGGAAGGATAACGGG..................... 17
....................................CAACGGTGGAAGGATAAC........................ 9
....................................CAACGGTGGAAGGATAACGGGC.................... 4
....................................CAACGGTGGAAGGATAACGGGCC................... 4
....................................CAACGGTGGAAGGATAACGGGCCG.................. 1
.....................................AACGGTGGAAGGATAACGG...................... 35
.....................................AACGGTGGAAGGATAACG....................... 29
.....................................AACGGTGGAAGGATAACGGG..................... 26
.....................................AACGGTGGAAGGATAACGGGCC................... 20
.....................................AACGGTGGAAGGATAACGGGC.................... 18
.....................................AACGGTGGAAGGATAACGGGCCG.................. 3
.....................................AACGGTGGAAGGATAACGGGCCGC................. 1
......................................ACGGTGGAAGGATAACGGGCC................... 28
......................................ACGGTGGAAGGATAACGGGC.................... 28
......................................ACGGTGGAAGGATAACGGG..................... 27
......................................ACGGTGGAAGGATAACGG...................... 16
......................................ACGGTGGAAGGATAACGGGCCGC................. 3
......................................ACGGTGGAAGGATAACGGGCCG.................. 3
.......................................CGGTGGAAGGATAACGGGCC................... 7
.......................................CGGTGGAAGGATAACGGGCCG.................. 4
.......................................CGGTGGAAGGATAACGGG..................... 4
.......................................CGGTGGAAGGATAACGGGC.................... 4
.......................................CGGTGGAAGGATAACGGGCCGCC................ 1
........................................GGTGGAAGGATAACGGGCC................... 6
........................................GGTGGAAGGATAACGGGCCGC................. 2
........................................GGTGGAAGGATAACGGGC.................... 1
.........................................GTGGAAGGATAACGGGCC................... 3
.........................................GTGGAAGGATAACGGGCCGC................. 3
.........................................GTGGAAGGATAACGGGCCG.................. 2
..........................................TGGAAGGATAACGGGCCGCCGCAC............ 1
...........................................GGAAGGATAACGGGCCGCCGCACT........... 3
...........................................GGAAGGATAACGGGCCGCC................ 2
...........................................GGAAGGATAACGGGCCGCCGCACTG.......... 1
...........................................GGAAGGATAACGGGCCGCCGCA............. 1
...........................................GGAAGGATAACGGGCCGC................. 1
.............................................AAGGATAACGGGCCGCCGCACT........... 3
.............................................AAGGATAACGGGCCGCCGCACTGCT........ 2
.............................................AAGGATAACGGGCCGCCGCA............. 1
.............................................AAGGATAACGGGCCGCCGCACTGC......... 1
.............................................AAGGATAACGGGCCGCCGCAC............ 1
..............................................AGGATAACGGGCCGCCGCACTGCT........ 7
..............................................AGGATAACGGGCCGCCGCACTG.......... 1
..............................................AGGATAACGGGCCGCCGCAC............ 1
..............................................AGGATAACGGGCCGCCGCACTGC......... 1
...............................................GGATAACGGGCCGCCGCACTGCT........ 7
...............................................GGATAACGGGCCGCCGCACTGC......... 1
................................................GATAACGGGCCGCCGCACTGCT........ 2
................................................GATAACGGGCCGCCGCAC............ 1
.................................................ATAACGGGCCGCCGCACTGCT........ 2
..................................................TAACGGGCCGCCGCACTGCTG....... 1
...................................................AACGGGCCGCCGCACTGCTGG...... osa-miR2906b 2
...................................................AACGGGCCGCCGCACTGCT........ 2
...................................................AACGGGCCGCCGCACTGC......... 1
....................................................ACGGGCCGCCGCACTGCT........ osa-miR2906b variant 13
....................................................ACGGGCCGCCGCACTGCTGGC..... 1
.....................................................CGGGCCGCCGCACTGCTGG...... 2
```

D. Examples where a single miRNA precursor produces distinct miRNAs (each miRNA is shown in a different color)

>osa-MIR1317

```
TATTATCGTTACCAATATCCTAGATTGCTTTCAAGGTCATTTCTTTAGGGAACCCCATTCCCATAAATTCAGTATCCAAAGCTGGTTGCTGTATCTATGGGAATAAGGGTTCCCTTACGAAATGATCTTGGACGTAATCTAGGAGTTTGCTGACGATAATG
(((((((((((.(((..(((((((((((.((((((((((((((...(((((((((.(((((((((.((.(((((.(((....))).))))).)).)))))))))..)))))))))...)))))))))))))).))))))))))).)..))).)))))))))))
.............AATATCCTAGATTGCTTTCAAG.............................................................................................................................. 1
...................CTAGATTGCTTTCAAGGTCAT......................................................................................................................... 2
....................TAGATTGCTTTCAAGGTCATT........................................................................................................................ 7
```

```
....................TAGATTGCTTTCAAGGTCAT......................................................................................................................... 1
.....................AGATTGCTTTCAAGGTCATTTCTT.................................................................................................................... osa-miR1317-5p.2  127
.....................AGATTGCTTTCAAGGTCATTTCT..................................................................................................................... 9
.....................AGATTGCTTTCAAGGTCATTTC...................................................................................................................... 4
.....................AGATTGCTTTCAAGGTCATT........................................................................................................................ 3
.....................AGATTGCTTTCAAGGTCATTT....................................................................................................................... 1
......................GATTGCTTTCAAGGTCATTTCTT.................................................................................................................... 3
.............................................TAGGGAACCCCATTCCCATAAA.............................................................................................. osa-miR1317-5p 1
.............................................................................AAAGCTGGTTGCTGTATCTATGGG............................................................ 1
...............................................................................AGCTGGTTGCTGTATCTATGGGAA.......................................................... 1
......................................................................................TGCTGTATCTATGGGAATAAGGGT................................................... 1
..............................................................................................CTATGGGAATAAGGGTTCCCTTAC........................................... 1
..............................................................................................CTATGGGAATAAGGGTTCCCTTA............................................ 1
...............................................................................................TATGGGAATAAGGGTTCCCTTAC........................................... 1
.............................................................................................................TTCCCTTACGAAATGATCTTGGAC............................ 19
.............................................................................................................TTCCCTTACGAAATGATCTTGG.............................. 7
.............................................................................................................TTCCCTTACGAAATGATCTTGGA............................. 1
.............................................................................................................TTCCCTTACGAAATGATCTT................................ 1
..............................................................................................................TCCCTTACGAAATGATCTTGGACG........................... 5
..............................................................................................................TCCCTTACGAAATGATCTTGGAC............................ 2
..............................................................................................................TCCCTTACGAAATGATCTTGG.............................. 2
..............................................................................................................TCCCTTACGAAATGATCTT................................ 1
..............................................................................................................TCCCTTACGAAATGATCTTGGA............................. 1
...............................................................................................................CCCTTACGAAATGATCTTGGACG........................... 2
.................................................................................................................CTTACGAAATGATCTTGGACGTAAT....................... 1
.................................................................................................................CTTACGAAATGATCTTGGACGTA......................... 1
..................................................................................................................TTACGAAATGATCTTGGACGTAAT....................... 907
..................................................................................................................TTACGAAATGATCTTGGACGTAA........................ 11
..................................................................................................................TTACGAAATGATCTTGGACGT.......................... 6
..................................................................................................................TTACGAAATGATCTTGGACGTA......................... 2
..................................................................................................................TTACGAAATGATCTTGGACG........................... 2
..................................................................................................................TTACGAAATGATCTTGGAC............................ 2
..................................................................................................................TTACGAAATGATCTTGGA............................. 1
...................................................................................................................TACGAAATGATCTTGGACGTAAT....................... 58
...................................................................................................................TACGAAATGATCTTGGACGTAATC...................... 3
...................................................................................................................TACGAAATGATCTTGGACGTA......................... 1
....................................................................................................................ACGAAATGATCTTGGACGTAAT....................... 7
.....................................................................................................................CGAAATGATCTTGGACGTAAT....................... 7
.....................................................................................................................CGAAATGATCTTGGACGTAATC...................... 3
.....................................................................................................................CGAAATGATCTTGGACGTAATCTAG................... 2
.....................................................................................................................CGAAATGATCTTGGACGTAATCTA.................... 1
......................................................................................................................GAAATGATCTTGGACGTAATCTAG................... osa-miR1317-3p 67
......................................................................................................................GAAATGATCTTGGACGTAAT....................... 14
......................................................................................................................GAAATGATCTTGGACGTAATC...................... 12
......................................................................................................................GAAATGATCTTGGACGTAATCT..................... 4
......................................................................................................................GAAATGATCTTGGACGTAA........................ 3
......................................................................................................................GAAATGATCTTGGACGTAATCTA.................... 3
.......................................................................................................................AAATGATCTTGGACGTAATCTAG................... 73
.......................................................................................................................AAATGATCTTGGACGTAATC...................... 9
.......................................................................................................................AAATGATCTTGGACGTAAT....................... 6
.......................................................................................................................AAATGATCTTGGACGTAA........................ 4
.......................................................................................................................AAATGATCTTGGACGTAATCTAGG.................. 1
........................................................................................................................AATGATCTTGGACGTAATCTAG................... 4
........................................................................................................................AATGATCTTGGACGTAAT....................... 1
.........................................................................................................................ATGATCTTGGACGTAATCTAG................... 2
..........................................................................................................................TGATCTTGGACGTAATCTAG................... 3
..........................................................................................................................TGATCTTGGACGTAATCTA.................... 1
...........................................................................................................................GATCTTGGACGTAATCTAGGA................. 4
...........................................................................................................................GATCTTGGACGTAATCTAG................... 1
```

>osa-MIR159a

```
GTTGTGGACGTTGAGCTCCTTTCGGTCCAAAAAGGGGTGTTGCTGTGGGTCGATTGAGCTGCTGGGTCATGGATCCCGTTAGCCTACTCCATGTTCATCATTCAGCTCGAGATCTGAAAGAAACTACTCCAATTTATACTAATAGTATGTGTGTAGATAGGAAAATGATGGAGTACTCGTTGTTGGGATAGGCTTATGGCTTGCATGCCCCAGGAGCTGCATCAACCCTACATGGACCCTCTTTGGATTGAAGGGAGCTCTGCATCTTTTGT
.....(((.((.(((((((((((((((((..(((((.(((..((((((.(((..((((.(((((.((((.(..((((.((((((..(((..((((((((((((........))))..........(((.((((((((...........)))))))).)))..))))))))..((.....)))))..))))))).))))..).)))).))))).))))..))).)))).)))..))))))))))))))))))))))))))).)).))).....
..........TTGAGCTCCTTTCGGTCCAAA................................................................................................................................................................................................................................................. 2
...........TGAGCTCCTTTCGGTCCAAAA................................................................................................................................................................................................................................................ 4
...........TGAGCTCCTTTCGGTCCAAAAA............................................................................................................................................................................................................................................... 2
............GAGCTCCTTTCGGTCCAAAAA............................................................................................................................................................................................................................................... 5
.............AGCTCCTTTCGGTCCAAAAA............................................................................................................................................................................................................................................... 1
......................................................TGAGCTGCTGGGTCATGGATCC.................................................................................................................................................................................................... 1
........................................................AGCTGCTGGGTCATGGATC..................................................................................................................................................................................................... 10
........................................................AGCTGCTGGGTCATGGAT...................................................................................................................................................................................................... 2
........................................................AGCTGCTGGGTCATGGATCC.................................................................................................................................................................................................... 1
.......................................................................................................................................................................................................CTTGCATGCCCCAGGAGCTGC.................................................... 2
........................................................................................................................................................................................................TTGCATGCCCCAGGAGCTGC.................................................... 9
........................................................................................................................................................................................................TTGCATGCCCCAGGAGCTGCA................................................... osa-miR159a.2 1
..............................................................................................................................................................................................................GCCCCAGGAGCTGCATCAACCCTA.......................................... 2
.................................................................................................................................................................................................................CCAGGAGCTGCATCAACCCTAC......................................... 1
............................................................................................................................................................................................................................ATCAACCCTACATGGACCCTC............................... 3
............................................................................................................................................................................................................................ATCAACCCTACATGGACCCT................................ 1
............................................................................................................................................................................................................................ATCAACCCTACATGGACCCTCT.............................. 1
............................................................................................................................................................................................................................ATCAACCCTACATGGACC.................................. 1
.............................................................................................................................................................................................................................................CCTCTTTGGATTGAAGGGAGC.............. 1
..............................................................................................................................................................................................................................................CTCTTTGGATTGAAGGGAGC.............. 1
...............................................................................................................................................................................................................................................TCTTTGGATTGAAGGGAG............... 4
...............................................................................................................................................................................................................................................TCTTTGGATTGAAGGGAGC.............. 4
...............................................................................................................................................................................................................................................TCTTTGGATTGAAGGGAGCT............. 1
................................................................................................................................................................................................................................................CTTTGGATTGAAGGGAGC.............. 22
................................................................................................................................................................................................................................................CTTTGGATTGAAGGGAGCT............. 3
................................................................................................................................................................................................................................................CTTTGGATTGAAGGGAGCTCT........... 2
.................................................................................................................................................................................................................................................TTTGGATTGAAGGGAGCT............. 2379
.................................................................................................................................................................................................................................................TTTGGATTGAAGGGAGCTCTG.......... osa-miR159a.1 132
.................................................................................................................................................................................................................................................TTTGGATTGAAGGGAGCTC............ 22
```

```
...............................................................................................................................................................................................................................................TTTGGATTGAAGGGAGCTCT.......... 12
...............................................................................................................................................................................................................................................TTTGGATTGAAGGGAGCTCTGC........ 7
................................................................................................................................................................................................................................................TTGGATTGAAGGGAGCTCTGC........ 15
................................................................................................................................................................................................................................................TTGGATTGAAGGGAGCTCT.......... 13
................................................................................................................................................................................................................................................TTGGATTGAAGGGAGCTCTG......... 2
................................................................................................................................................................................................................................................TTGGATTGAAGGGAGCTCTGCA....... 1
.................................................................................................................................................................................................................................................TGGATTGAAGGGAGCTCTG......... 1
```

>osa-MIR1850

```
ATGTGATGGAGATGCGATGGAAAGTTGGGAGATTGGGGGAAGTTGTGTGTGAACTAAACGTGGATTGGGGCCCTGTTTAGTTCACATCAATCTTCCTCCAAATTCCCAACTTTTCATCACATCACAATCACAT
(((((((.(.((((.((((((((((((((((.(((((((((((((.((((((((((((((.((.......)).))))))))))))))))).)))))))))).)))))))))))))))).)))).).)))))))
.........AGATGCGATGGAAAGTTGGGA....................................................................................................... 2
.........AGATGCGATGGAAAGTTGGGAGAT.................................................................................................... 1
............TGCGATGGAAAGTTGGGAGAT.................................................................................................... 1
..............CGATGGAAAGTTGGGAGATTG.................................................................................................. 2
..............CGATGGAAAGTTGGGAGATTGGGG............................................................................................... 1
.................TGGAAAGTTGGGAGATTGGGG............................................................................................... osa-miR1850.1  1247
.................TGGAAAGTTGGGAGATTG.................................................................................................. 178
.................TGGAAAGTTGGGAGATTGGG................................................................................................ 178
.................TGGAAAGTTGGGAGATTGG................................................................................................. 131
.................TGGAAAGTTGGGAGATTGGGGGAA............................................................................................ 8
..................GGAAAGTTGGGAGATTGGGGGAA............................................................................................ 2
..................GGAAAGTTGGGAGATTGGGG............................................................................................... 1
..................GGAAAGTTGGGAGATTGGGGGAAG........................................................................................... 1
....................AAAGTTGGGAGATTGGGGGAA............................................................................................ 5
....................AAAGTTGGGAGATTGGGGGAAGTT......................................................................................... 2
.....................AAGTTGGGAGATTGGGGGAAGTT......................................................................................... 1
...........................GGAGATTGGGGGAAGTTGTGTGT................................................................................... 1
...........................GGAGATTGGGGGAAGTTGTGTGTG.................................................................................. 1
............................GAGATTGGGGGAAGTTGTGTGT................................................................................... 1
................................TTGGGGGAAGTTGTGTGTGAAC............................................................................... 2
................................TTGGGGGAAGTTGTGTGTGAACTA............................................................................. 1
.................................TGGGGGAAGTTGTGTGTGAACTAAA........................................................................... 7
.................................TGGGGGAAGTTGTGTGTGAACTAA............................................................................ 1
...................................GGGGAAGTTGTGTGTGAACTAAAC.......................................................................... 2
......................................GAAGTTGTGTGTGAACTAAACGTGG...................................................................... osa-miR1850.2  20
......................................GAAGTTGTGTGTGAACTAAACGTG....................................................................... 19
......................................GAAGTTGTGTGTGAACTAAACG......................................................................... 1
.......................................AAGTTGTGTGTGAACTAAACGTGG...................................................................... 3
.......................................AAGTTGTGTGTGAACTAAACGTG....................................................................... 1
...........................................................................TTTAGTTCACATCAATCTT....................................... 1
.............................................................................TAGTTCACATCAATCTTCCTCCA................................. 1
.................................................................................................CCAAATTCCCAACTTTTCATC............... 18
.................................................................................................CCAAATTCCCAACTTTTCA................. 1
```

>osa-MIR444d

```
AGTTATTGCACATGGTGGCACCAAGCATGAGGCAACAACTGCATTACTTGCAAGAAAGGCACAAAATCATTAGATGATTACTTGTGGCTTTCTTGCAAGTTGTGCAGTTGCTGCCTCAAGCTTGCTGCCTCCCTCTGCCAAAT
.......(((...((.((((.(((((.(((((((.(((((((((.(((((((((((((.(((((((((((...))))))..))))).))))))))))))).))))))))).))))))).))))).)))).))...))).....
....................CCAAGCATGAGGCAACAACTG...................................................................................................... 1
.......................AGCATGAGGCAACAACTGCAT................................................................................................... 20
........................GCATGAGGCAACAACTGCATT.................................................................................................. 5
........................GCATGAGGCAACAACTGCATTACT............................................................................................... 1
.............................AGGCAACAACTGCATTACTTGCAA.......................................................................................... 2
.............................AGGCAACAACTGCATTACTTG............................................................................................. 2
..............................GGCAACAACTGCATTACTTGC............................................................................................ 11
................................CAACAACTGCATTACTTGCAA.......................................................................................... 15
.................................AACAACTGCATTACTTGCAAG......................................................................................... 7
.....................................ACTGCATTACTTGCAAGAAAG..................................................................................... 1
.......................................TGCATTACTTGCAAGAAAGGC................................................................................... 1
........................................GCATTACTTGCAAGAAAGGCACAA............................................................................... 1
..........................................ATTACTTGCAAGAAAGGCACA................................................................................ 1
...........................................TTACTTGCAAGAAAGGCACAA............................................................................... 1
............................................TACTTGCAAGAAAGGCACAAA.............................................................................. 1
.............................................ACTTGCAAGAAAGGCACAAAAT............................................................................ 1
.................................................................................TTGTGGCTTTCTTGCAAGTTG......................................... osa-miR444d.3  22
.................................................................................TTGTGGCTTTCTTGCAAGT........................................... 1
..................................................................................TGTGGCTTTCTTGCAAGTTGT........................................ 1
........................................................................................TTTCTTGCAAGTTGTGCAGTT.................................. 16
.........................................................................................TTCTTGCAAGTTGTGCAGTTG................................. 6
.........................................................................................TTCTTGCAAGTTGTGCAG.................................... 1
..........................................................................................TCTTGCAAGTTGTGCAGTTG................................. 1
............................................................................................TTGCAAGTTGTGCAGTTGCTG.............................. 3
.............................................................................................TGCAAGTTGTGCAGTTGCTGC............................. 15
.............................................................................................TGCAAGTTGTGCAGTTGCT............................... 2
......................................................................................................TGCAGTTGCTGCCTCAAGCTT.................... osa-miR444d.2  797
......................................................................................................TGCAGTTGCTGCCTCAAGCT..................... 5
......................................................................................................TGCAGTTGCTGCCTCAAGC...................... 3
......................................................................................................TGCAGTTGCTGCCTCAAGCTTG................... 1
.......................................................................................................GCAGTTGCTGCCTCAAGCTTG................... 26
.......................................................................................................GCAGTTGCTGCCTCAAGCTT.................... 4
.........................................................................................................AGTTGCTGCCTCAAGCTT.................... 1
...........................................................................................................TTGCTGCCTCAAGCTTGCTGC............... osa-miR444d.1  9
............................................................................................................TGCTGCCTCAAGCTTGCTGCC.............. 3
```

E. Examples where different miRNA precursors generate the same miRNAs (in red)

>osa-MIR166a

```
TGAAGCTATTTGCTTCTGAGTGGAATGTTGTCTGGTTCAAGGTCTCATGCACCTTGCGGTTTTGAGGATGATTTGTGCAAGGTTTTTCATTCCTCTCATCCGTGGGATCTCGGACCAGGCTTCATTCCCCTCAGAGATAGCTTCA
.((((((((....((((((.((((((..(((((((((.(((((((((.(((....)))...((((((((...............))).))))).....))))))))).)))))))))..)))))).)))))))))))))))).
..............TCTGAGTGGAATGTTGTCT................................................................................................................ 1
.....................GGAATGTTGTCTGGTTCAAGG....................................................................................................... 2
......................................................TGCGGTTTTGAGGATGATTTGTG.................................................................... 1
..........................................................................................................ATCTCGGACCAGGCTTCATTC.................. 3
..........................................................................................................ATCTCGGACCAGGCTTCATTCCC................ 1
...........................................................................................................TCTCGGACCAGGCTTCATTCC................. 1134
...........................................................................................................TCTCGGACCAGGCTTCATTC.................. 7
...........................................................................................................TCTCGGACCAGGCTTCAT.................... 3
...........................................................................................................TCTCGGACCAGGCTTCATT................... 3
...........................................................................................................TCTCGGACCAGGCTTCATTCCCC............... 2
...........................................................................................................TCTCGGACCAGGCTTCATTCCCCT.............. 1
............................................................................................................CTCGGACCAGGCTTCATTCCCC............... 16
............................................................................................................CTCGGACCAGGCTTCATTCCC................ 14
............................................................................................................CTCGGACCAGGCTTCATTC.................. 1
............................................................................................................CTCGGACCAGGCTTCATTCC................. 1
.............................................................................................................TCGGACCAGGCTTCATTCCCC............... osa-miR166a 45018
.............................................................................................................TCGGACCAGGCTTCATTCC................. 638
.............................................................................................................TCGGACCAGGCTTCATTC.................. 505
.............................................................................................................TCGGACCAGGCTTCATTCCC................ 404
.............................................................................................................TCGGACCAGGCTTCATTCCCCT.............. 88
.............................................................................................................TCGGACCAGGCTTCATTCCCCTC............. 2
..............................................................................................................CGGACCAGGCTTCATTCCCC............... 84
..............................................................................................................CGGACCAGGCTTCATTCCC................ 4
..............................................................................................................CGGACCAGGCTTCATTCC................. 4
..............................................................................................................CGGACCAGGCTTCATTCCCCT.............. 3
...............................................................................................................GGACCAGGCTTCATTCCCC............... 48
...............................................................................................................GGACCAGGCTTCATTCCC................ 2
...............................................................................................................GGACCAGGCTTCATTCCCCT.............. 1
```

>osa-MIR166b

```
GGGAGCCTTTTCATTTTGAGGGGAATGTTGTCTGGCTCGGGGCTACTTTTAATTTCTCTCTCTTTTGATATCTTCTTTTCTCGATCTCCTAGCTTGATCTTTTGATCTCTCAAATCGATCTTAAGAAAAAGATCAGTCAAAGAGATGAGAGTAGATGTCTGTAGATCTCGGACCAGGCTTCATTCCCCCCAAACAGAAGGGCTCCC
((((((((((...((((.((((((((..((((((..(((((((((.....((((((((((((((((((((((..(((((.((((.........((((....))))..........))))...))))))))))..))).)))))).))))).))))....)))).)))))..))))))..))))))))).))))..)))))))))))
................TGAGGGGAATGTTGTCTGGCTC........................................................................................................................................................................ 7
................TGAGGGGAATGTTGTCTGGCT......................................................................................................................................................................... 6
..................AGGGGAATGTTGTCTGGCT......................................................................................................................................................................... 2
..................AGGGGAATGTTGTCTGGCTC........................................................................................................................................................................ 1
..................AGGGGAATGTTGTCTGGCTCGGGG.................................................................................................................................................................... 1
...................GGGGAATGTTGTCTGGCTCGG...................................................................................................................................................................... 3
...................GGGGAATGTTGTCTGGCTC........................................................................................................................................................................ 1
.....................GGAATGTTGTCTGGCTCGGGG.................................................................................................................................................................... 28
.....................GGAATGTTGTCTGGCTCGGG..................................................................................................................................................................... 5
.....................GGAATGTTGTCTGGCTCG....................................................................................................................................................................... 1
.................................................................................................................................................................TAGATCTCGGACCAGGCTTCAT....................... 1
....................................................................................................................................................................ATCTCGGACCAGGCTTCATTC..................... 3
....................................................................................................................................................................ATCTCGGACCAGGCTTCATTCCC................... 1
.....................................................................................................................................................................TCTCGGACCAGGCTTCATTCC.................... 1134
.....................................................................................................................................................................TCTCGGACCAGGCTTCATTC..................... 7
.....................................................................................................................................................................TCTCGGACCAGGCTTCAT....................... 3
.....................................................................................................................................................................TCTCGGACCAGGCTTCATT...................... 3
.....................................................................................................................................................................TCTCGGACCAGGCTTCATTCCCC.................. 2
......................................................................................................................................................................CTCGGACCAGGCTTCATTCCCC.................. 16
......................................................................................................................................................................CTCGGACCAGGCTTCATTCCC................... 14
......................................................................................................................................................................CTCGGACCAGGCTTCATTC..................... 1
......................................................................................................................................................................CTCGGACCAGGCTTCATTCC.................... 1
.......................................................................................................................................................................TCGGACCAGGCTTCATTCCCC.................. osa-miR166b 45018
.......................................................................................................................................................................TCGGACCAGGCTTCATTCC.................... 638
.......................................................................................................................................................................TCGGACCAGGCTTCATTC..................... 505
.......................................................................................................................................................................TCGGACCAGGCTTCATTCCC................... 404
.......................................................................................................................................................................TCGGACCAGGCTTCATTCCCCC................. 2
........................................................................................................................................................................CGGACCAGGCTTCATTCCCC.................. 84
........................................................................................................................................................................CGGACCAGGCTTCATTCCC................... 4
........................................................................................................................................................................CGGACCAGGCTTCATTCC.................... 4
........................................................................................................................................................................CGGACCAGGCTTCATTCCCCC................ 2
.........................................................................................................................................................................GGACCAGGCTTCATTCCCC.................. 48
.........................................................................................................................................................................GGACCAGGCTTCATTCCC................... 2
...........................................................................................................................................................................ACCAGGCTTCATTCCCCCCAAA............. 1
```

>osa-MIR166c

```
GGCAGTTGAGGGGAATGTTGTCTGGTCCGAGACCTAACACCGGGCGGAATGGCGGATTCAGCTGCAGCTAAGCAAGCTAGGTGGGGGGTTTCGGACCAGGCTTCATTCCCCCAACTCAACTGTT
.((((((((((((((..((((((((((((((((..((((.(((.((((.....)))).(((.......)))..))).))))..))))))))))))))))..))))))))).....)))))))).
......TGAGGGGAATGTTGTCTGGTC................................................................................................. 1
...........GGAATGTTGTCTGGTCCGAGA............................................................................................ 11
............GAATGTTGTCTGGTCCGAGAC........................................................................................... 1
............GAATGTTGTCTGGTCCGAG............................................................................................. 1
................................................................................GTGGGGGGTTTCGGACCAGGCT...................... 1
...................................................................................GGGGGTTTCGGACCAGGCTTCATT................. 1
......................................................................................GGTTTCGGACCAGGCTTCATTCCC.............. 2
.......................................................................................GTTTCGGACCAGGCTTCATTC................ 12
.......................................................................................GTTTCGGACCAGGCTTCAT.................. 1
........................................................................................TTTCGGACCAGGCTTCATTCC............... 20
........................................................................................TTTCGGACCAGGCTTCATTCCC.............. 3
........................................................................................TTTCGGACCAGGCTTCATTCCCC............. 1
.........................................................................................TTCGGACCAGGCTTCATTCCC.............. 2552
```

```
.........................................................................................TTCGGACCAGGCTTCATTCC................ 25
.........................................................................................TTCGGACCAGGCTTCATTC................. 17
.........................................................................................TTCGGACCAGGCTTCATTCCCC.............. 2
.........................................................................................TTCGGACCAGGCTTCATT.................. 1
..........................................................................................TCGGACCAGGCTTCATTCCCC.............. osa-miR166c 45018
..........................................................................................TCGGACCAGGCTTCATTCC................ 638
..........................................................................................TCGGACCAGGCTTCATTC................. 505
..........................................................................................TCGGACCAGGCTTCATTCCC............... 404
..........................................................................................TCGGACCAGGCTTCATTCCCCC............. 2
...........................................................................................CGGACCAGGCTTCATTCCCC.............. 84
...........................................................................................CGGACCAGGCTTCATTCCC............... 4
...........................................................................................CGGACCAGGCTTCATTCC................ 4
...........................................................................................CGGACCAGGCTTCATTCCCCC............. 2
............................................................................................GGACCAGGCTTCATTCCCC.............. 48
............................................................................................GGACCAGGCTTCATTCCC............... 2
```

>osa-MIR166d

```
GAAGCTTTTTCACTTTGAGGGGAATGTTGTCTGGCTCGAGGTGCATGGAGAAACCTCTGATCGATCAGGTTTGATCTGTAGAGACTGATCTCGGACCAGGCTTCATTCCCCTCAAGTAAAGCTCC
..((((((...((((.((((((((((..((((((..((((((.((.((((....))))((((((......)))))).........))))))))..))))))..))))))))))))))))))))..
...............TGAGGGGAATGTTGTCTGGCTC........................................................................................ 7
...............TGAGGGGAATGTTGTCTGGCT......................................................................................... 6
.................AGGGGAATGTTGTCTGGCT......................................................................................... 2
.................AGGGGAATGTTGTCTGGCTC........................................................................................ 1
..................GGGGAATGTTGTCTGGCTC........................................................................................ 1
...................GGGAATGTTGTCTGGCTCGAG..................................................................................... 6
....................GGAATGTTGTCTGGCTCGAGG.................................................................................... 70
....................GGAATGTTGTCTGGCTCGAGGTGCATG.............................................................................. 6
....................GGAATGTTGTCTGGCTCGAG..................................................................................... 5
....................GGAATGTTGTCTGGCTCGAGGTGCAT............................................................................... 3
....................GGAATGTTGTCTGGCTCGAGGTGCATGG............................................................................. 1
....................GGAATGTTGTCTGGCTCGAGGTGCA................................................................................ 1
....................GGAATGTTGTCTGGCTCG....................................................................................... 1
.....................GAATGTTGTCTGGCTCGAGGT................................................................................... 1
......................AATGTTGTCTGGCTCGAGGTG.................................................................................. 3
.......................ATGTTGTCTGGCTCGAGGTGC................................................................................. 2
....................................................................................CTGATCTCGGACCAGGCT....................... 1
.......................................................................................ATCTCGGACCAGGCTTCATTC................. 3
.......................................................................................ATCTCGGACCAGGCTTCATTCCC............... 1
........................................................................................TCTCGGACCAGGCTTCATTCC................ 1134
........................................................................................TCTCGGACCAGGCTTCATTC................. 7
........................................................................................TCTCGGACCAGGCTTCAT................... 3
........................................................................................TCTCGGACCAGGCTTCATT.................. 3
........................................................................................TCTCGGACCAGGCTTCATTCCCC.............. 2
........................................................................................TCTCGGACCAGGCTTCATTCCCCT............. 1
.........................................................................................CTCGGACCAGGCTTCATTCCCC.............. 16
.........................................................................................CTCGGACCAGGCTTCATTCCC............... 14
.........................................................................................CTCGGACCAGGCTTCATTC................. 1
.........................................................................................CTCGGACCAGGCTTCATTCC................ 1
..........................................................................................TCGGACCAGGCTTCATTCCCC.............. osa-miR166d 45018
..........................................................................................TCGGACCAGGCTTCATTCC................ 638
..........................................................................................TCGGACCAGGCTTCATTC................. 505
..........................................................................................TCGGACCAGGCTTCATTCCC............... 404
..........................................................................................TCGGACCAGGCTTCATTCCCCT............. 88
..........................................................................................TCGGACCAGGCTTCATTCCCCTC............ 2
...........................................................................................CGGACCAGGCTTCATTCCCC.............. 84
...........................................................................................CGGACCAGGCTTCATTCCC............... 4
...........................................................................................CGGACCAGGCTTCATTCC................ 4
...........................................................................................CGGACCAGGCTTCATTCCCCT............. 3
............................................................................................GGACCAGGCTTCATTCCCC.............. 48
............................................................................................GGACCAGGCTTCATTCCC............... 2
............................................................................................GGACCAGGCTTCATTCCCCT............. 1
```

>osa-MIR166f

```
CGATCATCTTGTTGAGAGGAATGTCGTCTGGCCTGAGATCGTACCACAGTGGTGGGTACACGTGGACGGTCTCGGACCAGGCTTCATTCCCCTCAACAACTCGTCGG
((((....((((((((.((((((..((((((.((((((((((.((((.(((.......))))))))))))))))).))))))..)))))).))))))))...)))).
..........GTTGAGAGGAATGTCGTCTGGC........................................................................... 1
..........GTTGAGAGGAATGTCGTCTGG............................................................................ 1
............TGAGAGGAATGTCGTCTGGCC.......................................................................... 1
..............................GCCTGAGATCGTACCACAGT......................................................... 1
.....................................................................TCTCGGACCAGGCTTCATTCC................. 1134
.....................................................................TCTCGGACCAGGCTTCATTC.................. 7
.....................................................................TCTCGGACCAGGCTTCAT.................... 3
.....................................................................TCTCGGACCAGGCTTCATT................... 3
.....................................................................TCTCGGACCAGGCTTCATTCCCC............... 2
.....................................................................TCTCGGACCAGGCTTCATTCCCCT.............. 1
......................................................................CTCGGACCAGGCTTCATTCCCC............... 16
......................................................................CTCGGACCAGGCTTCATTCCC................ 14
......................................................................CTCGGACCAGGCTTCATTC.................. 1
......................................................................CTCGGACCAGGCTTCATTCC................. 1
.......................................................................TCGGACCAGGCTTCATTCCCC............... osa-miR166f 45018
.......................................................................TCGGACCAGGCTTCATTCC................. 638
.......................................................................TCGGACCAGGCTTCATTC.................. 505
.......................................................................TCGGACCAGGCTTCATTCCC................ 404
.......................................................................TCGGACCAGGCTTCATTCCCCT.............. 88
```

```
.......................................................................TCGGACCAGGCTTCATTCCCCTC............. 2
........................................................................CGGACCAGGCTTCATTCCCC............... 84
........................................................................CGGACCAGGCTTCATTCCC................ 4
........................................................................CGGACCAGGCTTCATTCC................. 4
........................................................................CGGACCAGGCTTCATTCCCCT.............. 3
.........................................................................GGACCAGGCTTCATTCCCC............... 48
.........................................................................GGACCAGGCTTCATTCCC................ 2
.........................................................................GGACCAGGCTTCATTCCCCT.............. 1
```

>osa-MIR166n

```
TGATCCATGGCTGTTGAGAGGAATGACGTCCGGTCTGAAGATCGCCGTCCCCAGGCGGTGGCTTCGGACCAGGCTTCATTCCCCATGACTCATGGAACCC
...(((((((..((((.(.(((((((.(((.(((((((((.((((((((....))))))))))))))))).))).))))))).).)))))))))))....
....................GAATGACGTCCGGTCTGAAGA........................................................... 72
....................GAATGACGTCCGGTCTGAAG............................................................ 1
.....................AATGACGTCCGGTCTGAAGATCGC....................................................... 1
.....................................................GGCGGTGGCTTCGGACCAGGCT......................... 1
...........................................................GGCTTCGGACCAGGCTTCATTCCC................. 3
.............................................................CTTCGGACCAGGCTTCATTCCC................. 1
..............................................................TTCGGACCAGGCTTCATTCCC................. 2552
..............................................................TTCGGACCAGGCTTCATTCC.................. 25
..............................................................TTCGGACCAGGCTTCATTC................... 17
..............................................................TTCGGACCAGGCTTCATTCCCC................ 2
..............................................................TTCGGACCAGGCTTCATT.................... 1
...............................................................TCGGACCAGGCTTCATTCCCC................ osa-miR166n 45018
...............................................................TCGGACCAGGCTTCATTCC.................. 638
...............................................................TCGGACCAGGCTTCATTC................... 505
...............................................................TCGGACCAGGCTTCATTCCC................. 404
...............................................................TCGGACCAGGCTTCATTCCCCA............... 47
................................................................CGGACCAGGCTTCATTCCCC................ 84
................................................................CGGACCAGGCTTCATTCCC................. 4
................................................................CGGACCAGGCTTCATTCC.................. 4
.................................................................GGACCAGGCTTCATTCCCC................ 48
.................................................................GGACCAGGCTTCATTCCC................. 2
.................................................................GGACCAGGCTTCATTCCCCA............... 1
```

>osa-MIR167d

```
CATTAGGAGCTGAAGCTGCCAGCATGATCTGATGAGTGCTTATTAGGTGAGGGCAGAATTGACTGCCAAAACAAAGATCAGATCATGCTGTGCAGTTTCATCTGCTTGTG
(((.((.((.(((((((((((((((((((((((....(((.....)))...(((((......))))).........)))))))))))))).))))))))).)).)).)))
.........CTGAAGCTGCCAGCATGATCTGA.............................................................................. 9
.........CTGAAGCTGCCAGCATGATCT................................................................................ 1
..........TGAAGCTGCCAGCATGATCTGA.............................................................................. 50021
..........TGAAGCTGCCAGCATGATCTG............................................................................... osa-miR167d 595
..........TGAAGCTGCCAGCATGATCT................................................................................ 526
..........TGAAGCTGCCAGCATGATC................................................................................. 181
..........TGAAGCTGCCAGCATGATCTGAT............................................................................. 74
..........TGAAGCTGCCAGCATGAT.................................................................................. 33
...........GAAGCTGCCAGCATGATCTGA.............................................................................. 38
...........GAAGCTGCCAGCATGATC................................................................................. 1
............AAGCTGCCAGCATGATCTGATG............................................................................ 2
............AAGCTGCCAGCATGATCTGA.............................................................................. 2
.............AGCTGCCAGCATGATCTGATGA........................................................................... 1
```

>osa-MIR167f

```
CACAAGTGGATGAAGCTGCCAGCATGATCTGATCACAGTAGTTCTCTAGCTGATGATGATTTACAAAACCTAGAGACATGCATCAGATCATCTGGCAGTTTCATCTTCTCATG
((..((.(((((((((((((((.((((((((((....(((..(((((((.(..((........))..).)))))))..))))))))))))))))))))))))))))).))..))
.........ATGAAGCTGCCAGCATGATCTG.................................................................................. 3
..........TGAAGCTGCCAGCATGATCTGA................................................................................. 50021
..........TGAAGCTGCCAGCATGATCTG.................................................................................. osa-miR167f 595
..........TGAAGCTGCCAGCATGATCT................................................................................... 526
..........TGAAGCTGCCAGCATGATC.................................................................................... 181
..........TGAAGCTGCCAGCATGATCTGAT................................................................................ 74
..........TGAAGCTGCCAGCATGAT..................................................................................... 33
...........GAAGCTGCCAGCATGATCTGA................................................................................. 38
...........GAAGCTGCCAGCATGATC.................................................................................... 1
............AAGCTGCCAGCATGATCTGA................................................................................. 2
............AAGCTGCCAGCATGATCTGATC............................................................................... 1
..................................................................................TCAGATCATCTGGCAGTTT............ 1
..................................................................................TCAGATCATCTGGCAGTTTCA.......... 1
...................................................................................CAGATCATCTGGCAGTTTCA.......... 1
...................................................................................CAGATCATCTGGCAGTTTCATC........ 1
....................................................................................AGATCATCTGGCAGTTTCATC........ 44
....................................................................................AGATCATCTGGCAGTTTCAT......... 2
```

>osa-MIR167g

```
CATAAGCAGGTGAAGCTGCCAGCATGATCTGAAAGCATCTCAAACCAGCGATCAGATCATCCGGCAGCTTCATCTTCTCATG
(((.((.(((((((((((((.(.((((((((((..((.........))..)))))))))).))))))))))))))).)).)))
.......AGGTGAAGCTGCCAGCATGATC..................................................... 3
..........TGAAGCTGCCAGCATGATCTGA.................................................. 50021
..........TGAAGCTGCCAGCATGATCTG................................................... osa-miR167g 595
..........TGAAGCTGCCAGCATGATCT.................................................... 526
..........TGAAGCTGCCAGCATGATCTGAA................................................. 261
..........TGAAGCTGCCAGCATGATC..................................................... 181
..........TGAAGCTGCCAGCATGATCTGAAA................................................ 66
```

```
..........TGAAGCTGCCAGCATGAT...................................................... 33
...........GAAGCTGCCAGCATGATCTGA.................................................. 38
...........GAAGCTGCCAGCATGATC..................................................... 1
............AAGCTGCCAGCATGATCTGA.................................................. 2
............AAGCTGCCAGCATGATCTGAAAGCA............................................. 1
..................................................ATCAGATCATCCGGCAGCTTCATC........ 1
.....................................................AGATCATCCGGCAGCTTCATC........ 61
.....................................................AGATCATCCGGCAGCTTCA.......... 1
......................................................GATCATCCGGCAGCTTCATC........ 9
.......................................................ATCATCCGGCAGCTTCATCTTC..... 1
........................................................TCATCCGGCAGCTTCATCTTC..... 70
........................................................TCATCCGGCAGCTTCATCT....... 2
........................................................TCATCCGGCAGCTTCATCTTCT.... 1
```

>osa-MIR167h

```
CACAAGTTGGTGAAGCTGCCAGCATGATCTGATGATGATGATGATCCACCTCTCTCATCTGTGTTCTTGATTAATTACGGATCAATCGATCAGGTCATGCTGTAGTTTCATCTGCTGGTT
.((.(((.(((((((((((.(((((((((((((..((((..((((((.......(((..........)))........)))))))))))))))))))))))))))))))))).))).)).
..........TGAAGCTGCCAGCATGATCTGA........................................................................................ 50021
..........TGAAGCTGCCAGCATGATCTG......................................................................................... osa-miR167h 595
..........TGAAGCTGCCAGCATGATCT.......................................................................................... 526
..........TGAAGCTGCCAGCATGATC........................................................................................... 181
..........TGAAGCTGCCAGCATGATCTGAT....................................................................................... 74
..........TGAAGCTGCCAGCATGAT............................................................................................ 33
...........GAAGCTGCCAGCATGATCTGA........................................................................................ 38
...........GAAGCTGCCAGCATGATC........................................................................................... 1
............AAGCTGCCAGCATGATCTGATG...................................................................................... 2
............AAGCTGCCAGCATGATCTGA........................................................................................ 2
.............AGCTGCCAGCATGATCTGATGA..................................................................................... 1
..........................................................................................CAGGTCATGCTGTAGTTTCAT......... 1
...........................................................................................AGGTCATGCTGTAGTTTCATC........ 205
...........................................................................................AGGTCATGCTGTAGTTTCATCT....... 9
...........................................................................................AGGTCATGCTGTAGTTTCAT......... 5
...........................................................................................AGGTCATGCTGTAGTTTCA.......... 2
```

>osa-MIR167j

```
TTGTGATGTGTGCACCTTAAGCAGCTGAAGCTGCCAGCATGATCTGATCTTTTGCGATCTCTTTTTTTATCTGAATAAGTTGATGGAAATATTGGGTTCCTAAGATTCAGATCGTGCTGCGCAGTTTCATCTGCTAATCGATGCACTACACTGTGAATTT
(..(..(((((((((..(.(((((.((((((((((((((((((((((((((..(.((.(((..((((((((..........))))))))....))).))).)))).)))))))))))).)))))))))).))))).)..).))))).)))..)..)....
........................CTGAAGCTGCCAGCATGATCTGA................................................................................................................. 9
........................CTGAAGCTGCCAGCATGATCT................................................................................................................... 1
.........................TGAAGCTGCCAGCATGATCTGA................................................................................................................. 50021
.........................TGAAGCTGCCAGCATGATCTG.................................................................................................................. osa-miR167j 595
.........................TGAAGCTGCCAGCATGATCT................................................................................................................... 526
.........................TGAAGCTGCCAGCATGATC.................................................................................................................... 181
.........................TGAAGCTGCCAGCATGATCTGAT................................................................................................................ 74
.........................TGAAGCTGCCAGCATGAT..................................................................................................................... 33
..........................GAAGCTGCCAGCATGATCTGA................................................................................................................. 38
..........................GAAGCTGCCAGCATGATC.................................................................................................................... 1
...........................AAGCTGCCAGCATGATCTGA................................................................................................................. 2
...........................AAGCTGCCAGCATGATCTGATC............................................................................................................... 1
............................................................................................................AGATCGTGCTGCGCAGTTTCATC............................. 3
............................................................................................................AGATCGTGCTGCGCAGTTTCA............................... 1
```

>osa-MIR1861b

```
TTGCATATTCTTAGGCCCGATCTTGAGGCAGGAACTGAGTAGTTCGTGAACTCGCTCTATGTTGTTCGTTCACCCACTATTCGGTTCCTGTCCCAAGATCGAGTCTGTGAATATACAA
.((.((((((.(((((.((((((((.(((((((((((((((((..((((((..((........))..))))))..)))))))))))))))))).)))))))).))))).)))))).)).
..............GCCCGATCTTGAGGCAGGAACTGA................................................................................ 1
.................CGATCTTGAGGCAGGAACTGAG............................................................................... osa-miR1861b 3700
.................CGATCTTGAGGCAGGAACTGA................................................................................ 1180
.................CGATCTTGAGGCAGGAACTG................................................................................. 12
.................CGATCTTGAGGCAGGAACTGAGTA............................................................................. 9
.................CGATCTTGAGGCAGGAACTGAGT.............................................................................. 4
.................CGATCTTGAGGCAGGAACT.................................................................................. 3
.................CGATCTTGAGGCAGGAAC................................................................................... 3
..................GATCTTGAGGCAGGAACTGAG............................................................................... 6727
..................GATCTTGAGGCAGGAACTGA................................................................................ 1994
..................GATCTTGAGGCAGGAACTG................................................................................. 14
..................GATCTTGAGGCAGGAACTGAGTA............................................................................. 11
..................GATCTTGAGGCAGGAACTGAGT.............................................................................. 9
..................GATCTTGAGGCAGGAACT.................................................................................. 4
...................ATCTTGAGGCAGGAACTGAGTAGT........................................................................... 1
....................TCTTGAGGCAGGAACTGAGTA............................................................................. 270
....................TCTTGAGGCAGGAACTGAG............................................................................... 25
....................TCTTGAGGCAGGAACTGAGT.............................................................................. 22
.....................CTTGAGGCAGGAACTGAG............................................................................... 7
.....................CTTGAGGCAGGAACTGAGTAG............................................................................ 6
......................TTGAGGCAGGAACTGAGTAGT........................................................................... 33
......................TTGAGGCAGGAACTGAGTAG............................................................................ 5
.......................TGAGGCAGGAACTGAGTAGTT.......................................................................... 1
.......................TGAGGCAGGAACTGAGTAGTTCGT....................................................................... 1
..............................................................................ATTCGGTTCCTGTCCCAAGATCGA................ 1
................................................................................TCGGTTCCTGTCCCAAGATCG................. 1
.................................................................................CGGTTCCTGTCCCAAGATCGAG............... 3
.................................................................................CGGTTCCTGTCCCAAGATCGA................ 1
.................................................................................CGGTTCCTGTCCCAAGATCGAGT.............. 1
```

```
..................................................................................CGGTTCCTGTCCCAAGATC.................. 1
..................................................................................GGTTCCTGTCCCAAGATCGAG............... 2
........................................................................................TGTCCCAAGATCGAGTCTGT.......... 2
```

>osa-MIR1861f

```
TGCGTATTCTTAGGCCCGATCTTGAGGCAGGAACTGAGTAGTTGTTGAACTTGCTCTATGTTGTTCGTTCACCCACTATTCGGTTCCTGTCCCAAGATCGAGTCTATGAATATACA
.(..(((((.(((((.((((((((.(((((((((((((((((.(.(((((..((........))..))))).).))))))))))))))))).)))))))).))))).)))))..).
.............GCCCGATCTTGAGGCAGGAACTGA............................................................................... 1
................CGATCTTGAGGCAGGAACTGAG.............................................................................. osa-miR1861f 3700
................CGATCTTGAGGCAGGAACTGA............................................................................... 1180
................CGATCTTGAGGCAGGAACTG................................................................................ 12
................CGATCTTGAGGCAGGAACTGAGTA............................................................................ 9
................CGATCTTGAGGCAGGAACTGAGT............................................................................. 4
................CGATCTTGAGGCAGGAACT................................................................................. 3
................CGATCTTGAGGCAGGAAC.................................................................................. 3
.................GATCTTGAGGCAGGAACTGAG.............................................................................. 6727
.................GATCTTGAGGCAGGAACTGA............................................................................... 1994
.................GATCTTGAGGCAGGAACTG................................................................................ 14
.................GATCTTGAGGCAGGAACTGAGTA............................................................................ 11
.................GATCTTGAGGCAGGAACTGAGT............................................................................. 9
.................GATCTTGAGGCAGGAACT................................................................................. 4
..................ATCTTGAGGCAGGAACTGAGTAGT.......................................................................... 1
...................TCTTGAGGCAGGAACTGAGTA............................................................................ 270
...................TCTTGAGGCAGGAACTGAG.............................................................................. 25
...................TCTTGAGGCAGGAACTGAGT............................................................................. 22
....................CTTGAGGCAGGAACTGAG.............................................................................. 7
....................CTTGAGGCAGGAACTGAGTAG........................................................................... 6
.....................TTGAGGCAGGAACTGAGTAGT.......................................................................... 33
.....................TTGAGGCAGGAACTGAGTAG........................................................................... 5
......................TGAGGCAGGAACTGAGTAGTT......................................................................... 1
.............................................................................ATTCGGTTCCTGTCCCAAGATCGA............... 1
...............................................................................TCGGTTCCTGTCCCAAGATCG................ 1
................................................................................CGGTTCCTGTCCCAAGATCGAG.............. 3
................................................................................CGGTTCCTGTCCCAAGATCGA............... 1
................................................................................CGGTTCCTGTCCCAAGATCGAGT............. 1
................................................................................CGGTTCCTGTCCCAAGATC................. 1
.................................................................................GGTTCCTGTCCCAAGATCGAG.............. 2
```

>osa-MIR1861i

```
GGCCCGATCTTGAGGCAGGAACTGAGTAGTTGGTGAACTCGCTCTATGTTGTTCGTTCACTTGCTACACGGTTCCTGTCCCAAGATCGAGTC
(((.((((((((.(((((((((((.(((((.(((((((..((........))..))))))).))))).))))))))))).)))))))).)))
.GCCCGATCTTGAGGCAGGAACTGA................................................................... 1
....CGATCTTGAGGCAGGAACTGAG.................................................................. osa-miR1861i 3700
....CGATCTTGAGGCAGGAACTGA................................................................... 1180
....CGATCTTGAGGCAGGAACTG.................................................................... 12
....CGATCTTGAGGCAGGAACTGAGTA................................................................ 9
....CGATCTTGAGGCAGGAACTGAGT................................................................. 4
....CGATCTTGAGGCAGGAACT..................................................................... 3
....CGATCTTGAGGCAGGAAC...................................................................... 3
.....GATCTTGAGGCAGGAACTGAG.................................................................. 6727
.....GATCTTGAGGCAGGAACTGA................................................................... 1994
.....GATCTTGAGGCAGGAACTG.................................................................... 14
.....GATCTTGAGGCAGGAACTGAGTA................................................................ 11
.....GATCTTGAGGCAGGAACTGAGT................................................................. 9
.....GATCTTGAGGCAGGAACT..................................................................... 4
......ATCTTGAGGCAGGAACTGAGTAGT.............................................................. 1
.......TCTTGAGGCAGGAACTGAGTA................................................................ 270
.......TCTTGAGGCAGGAACTGAG.................................................................. 25
.......TCTTGAGGCAGGAACTGAGT................................................................. 22
........CTTGAGGCAGGAACTGAG.................................................................. 7
........CTTGAGGCAGGAACTGAGTAG............................................................... 6
.........TTGAGGCAGGAACTGAGTAGT.............................................................. 33
.........TTGAGGCAGGAACTGAGTAG............................................................... 5
.........TTGAGGCAGGAACTGAGTAGTTGG........................................................... 2
..........TGAGGCAGGAACTGAGTAGTTGGT.......................................................... 1
..........TGAGGCAGGAACTGAGTAGTTGG........................................................... 1
..........TGAGGCAGGAACTGAGTAGTT............................................................. 1
...........GAGGCAGGAACTGAGTAGTTGGTG......................................................... 1
...............CAGGAACTGAGTAGTTGGTGA........................................................ 5
................AGGAACTGAGTAGTTGGTGAA....................................................... 5
................AGGAACTGAGTAGTTGGTGA........................................................ 3
................AGGAACTGAGTAGTTGGT.......................................................... 1
.......................GAGTAGTTGGTGAACTCGCTCTAT............................................. 1
........................................................TCACTTGCTACACGGTTCCTGTCC............ 1
.................................................................ACACGGTTCCTGTCCCAAGATCGA... 1
..................................................................CACGGTTCCTGTCCCAAGATCGAG.. 2
..................................................................CACGGTTCCTGTCCCAAGATCGA... 1
....................................................................CGGTTCCTGTCCCAAGATCGAG.. 3
....................................................................CGGTTCCTGTCCCAAGATCGA... 1
....................................................................CGGTTCCTGTCCCAAGATCGAGT. 1
....................................................................CGGTTCCTGTCCCAAGATC..... 1
.....................................................................GGTTCCTGTCCCAAGATCGAG.. 2
```

>osa-MIR1861l

```
TCGTGTTGCATATTCTTAGGCCCGATCTTGAGGCAGGAACTGAGTAGTTGGTGAACTCGCTCTATGTTGTTCATTCACTCGCGACACGGTTCCTGTCCCAAGATCGGGTCTGTGAATATACAAATGTGA
(((..((..((((((.((((((((((((((.(((((((((((.((.((.((((((...((........))...)))))).)).)).))))))))))).)))))))))))))).))))))...))..)))
...................GCCCGATCTTGAGGCAGGAACTGA...................................................................................... 1
......................CGATCTTGAGGCAGGAACTGAG..................................................................................... osa-miR1861l 3700
......................CGATCTTGAGGCAGGAACTGA...................................................................................... 1180
......................CGATCTTGAGGCAGGAACTG....................................................................................... 12
......................CGATCTTGAGGCAGGAACTGAGTA................................................................................... 9
......................CGATCTTGAGGCAGGAACTGAGT.................................................................................... 4
......................CGATCTTGAGGCAGGAACT........................................................................................ 3
......................CGATCTTGAGGCAGGAAC......................................................................................... 3
.......................GATCTTGAGGCAGGAACTGAG..................................................................................... 6727
.......................GATCTTGAGGCAGGAACTGA...................................................................................... 1994
.......................GATCTTGAGGCAGGAACTG....................................................................................... 14
.......................GATCTTGAGGCAGGAACTGAGTA................................................................................... 11
.......................GATCTTGAGGCAGGAACTGAGT.................................................................................... 9
.......................GATCTTGAGGCAGGAACT........................................................................................ 4
........................ATCTTGAGGCAGGAACTGAGTAGT................................................................................. 1
.........................TCTTGAGGCAGGAACTGAGTA................................................................................... 270
.........................TCTTGAGGCAGGAACTGAG..................................................................................... 25
.........................TCTTGAGGCAGGAACTGAGT.................................................................................... 22
..........................CTTGAGGCAGGAACTGAG..................................................................................... 7
..........................CTTGAGGCAGGAACTGAGTAG.................................................................................. 6
...........................TTGAGGCAGGAACTGAGTAGT................................................................................. 33
...........................TTGAGGCAGGAACTGAGTAG.................................................................................. 5
...........................TTGAGGCAGGAACTGAGTAGTTGG.............................................................................. 2
............................TGAGGCAGGAACTGAGTAGTTGGT............................................................................. 1
............................TGAGGCAGGAACTGAGTAGTTGG.............................................................................. 1
............................TGAGGCAGGAACTGAGTAGTT................................................................................ 1
.............................GAGGCAGGAACTGAGTAGTTGGTG............................................................................ 1
.................................CAGGAACTGAGTAGTTGGTGA........................................................................... 5
..................................AGGAACTGAGTAGTTGGTGAA.......................................................................... 5
..................................AGGAACTGAGTAGTTGGTGA........................................................................... 3
..................................AGGAACTGAGTAGTTGGT............................................................................. 1
.........................................GAGTAGTTGGTGAACTCGCTCTAT................................................................ 1
....................................................................................CACGGTTCCTGTCCCAAGATCGGG..................... 3
.....................................................................................ACGGTTCCTGTCCCAAGATCGGG..................... 1
......................................................................................CGGTTCCTGTCCCAAGATCGGG..................... 7
......................................................................................CGGTTCCTGTCCCAAGATCGG...................... 1
......................................................................................CGGTTCCTGTCCCAAGATC........................ 1
.......................................................................................GGTTCCTGTCCCAAGATCGGG..................... 9
.......................................................................................GGTTCCTGTCCCAAGATCGG...................... 1
...........................................................................................CCTGTCCCAAGATCGGGTCTG................. 1
...........................................................................................CCTGTCCCAAGATCGGGTCTGT................ 1
............................................................................................CTGTCCCAAGATCGGGTCTGT................ 7
............................................................................................CTGTCCCAAGATCGGGTCT.................. 1
```
