# Supplementary material for: Identification and Expression Analysis of microRNAs at the Grain Filling Stage in Rice(Oryza sativa L.)via Deep Sequencing
Source: PLoS One. 2013 Mar 1;8(3):e57863. doi: 10.1371/journal.pone.0057863 (PMC3585941; doi:10.1371/journal.pone.0057863)
Supplement: Figure S2 — The complete set of clusters of differentially expressed known miRNAs based on K-means support. The four points from left to right on the x-axis represent the 5 DAF, 7 DAF, 12 DAF and 17 DAF stages, respectively; the y-axis corresponds to the log2 value of the TPM. (PDF) [file pone.0057863.s002.pdf]

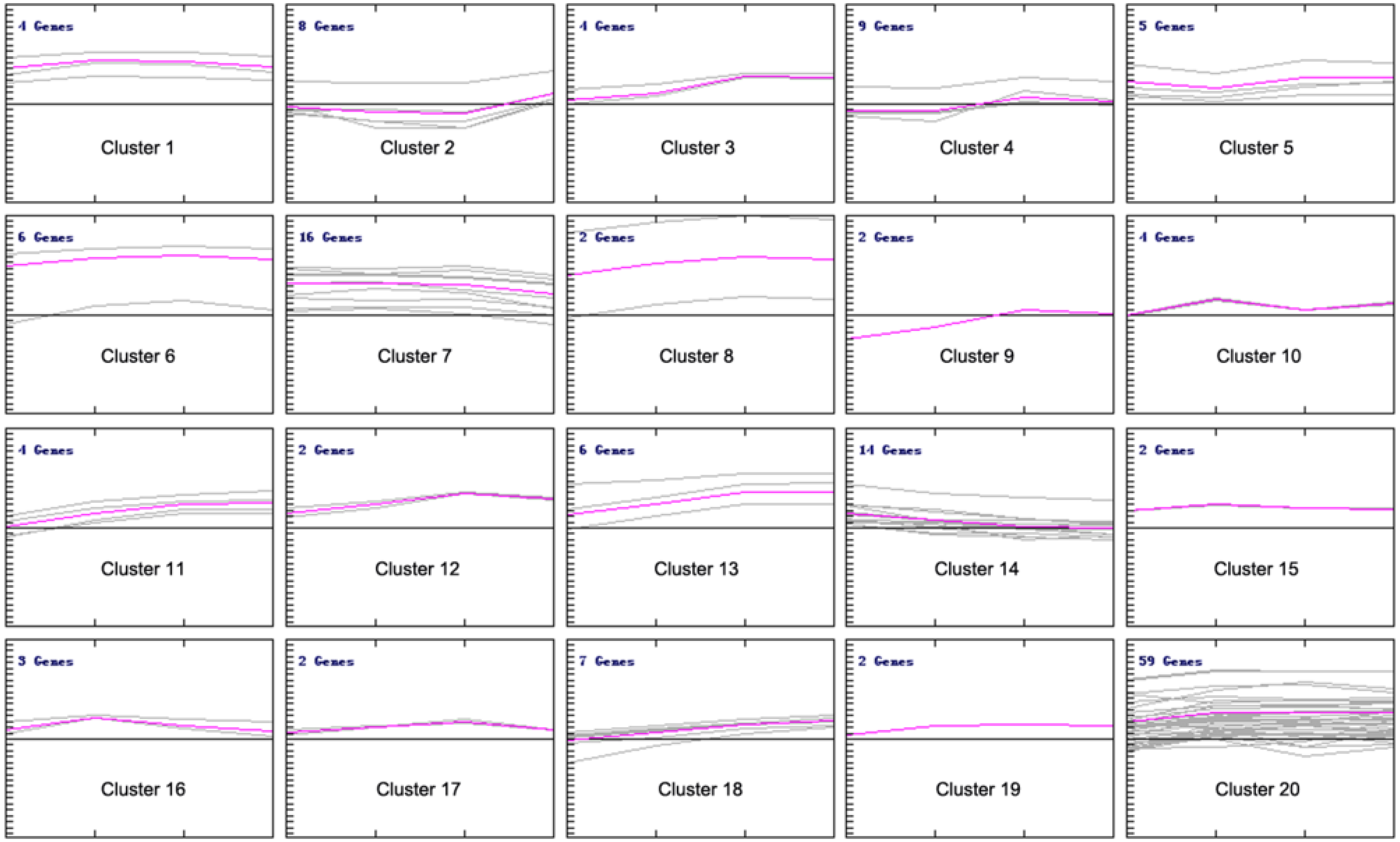

Figure S2. The complete set of clusters of differentially expressed known miRNAs based on K-means support. The four points from left to right on the x-axis represent the 5 DAF,7 DAF,12 DAF and 17 DAF stages, respectively; the y-axis corresponds to the log2 value of the TPM.
