# Supplementary material for: Identification and Expression Analysis of microRNAs at the Grain Filling Stage in Rice(Oryza sativa L.)via Deep Sequencing
Source: PLoS One. 2013 Mar 1;8(3):e57863. doi: 10.1371/journal.pone.0057863 (PMC3585941; doi:10.1371/journal.pone.0057863)
Supplement: Figure S3 — Graphical results of GO analysis of the targets of the differentially expressed known miRNAs. Targets with GO terms corresponding to (A) biological processes, (B) molecular functions and (C) cellular components. The GO terms and their serial numbers are represented as boxes. For each significant term, the box includes the GO term, adjusted p-value (in parentheses), item number mapping the GO term in the query list and background, and the total numbers of items in the query list and background. The color scale indicates the p-value cutoff levels for each GO term, where a higher statistical significance corresponds to a darker and redder the color. (PDF) [file pone.0057863.s003.pdf]

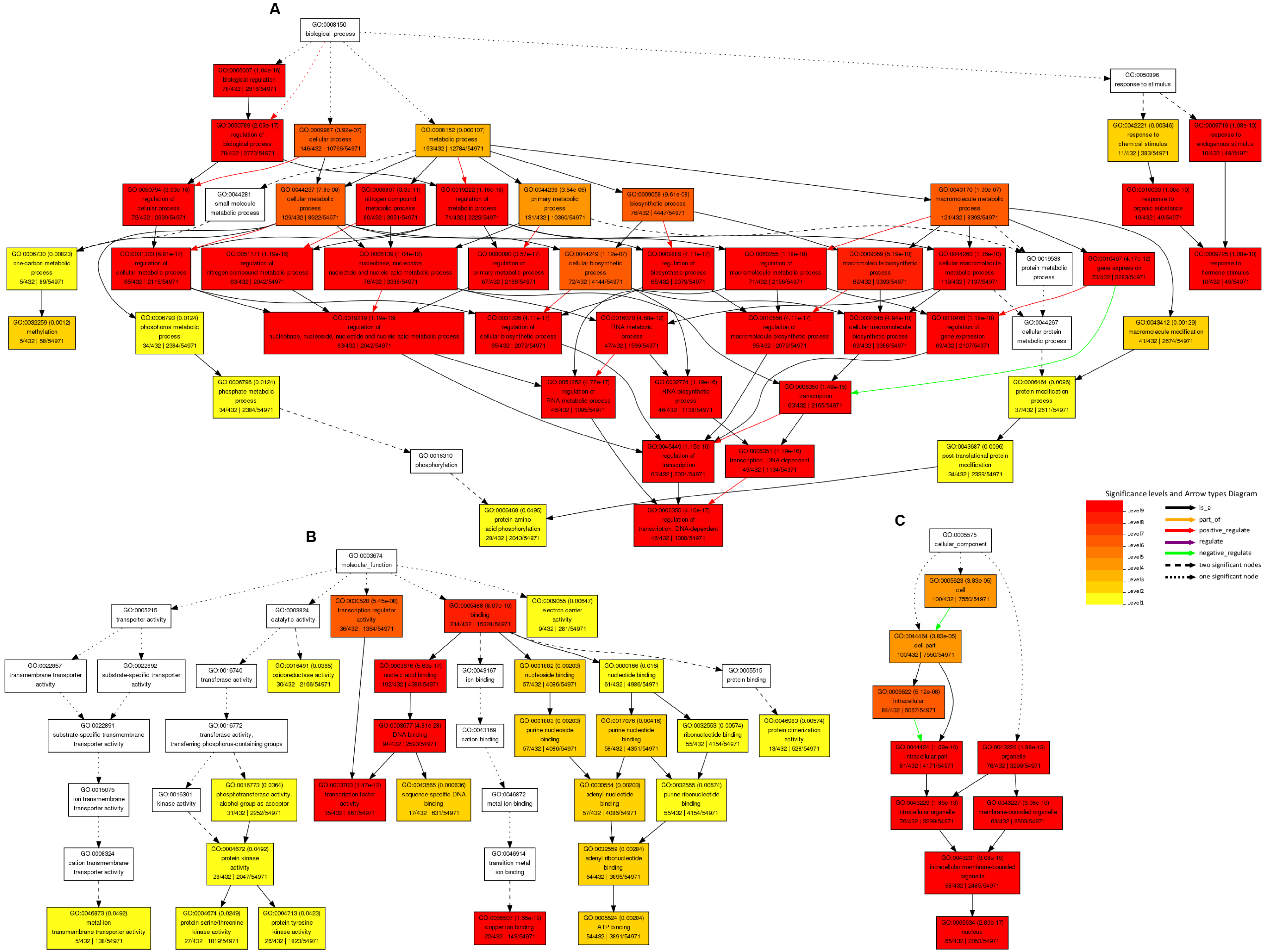

A
GO:0008150 biological_process
GO:0065007 (1.04e-16) biological regulation 78/432 | 2916/54971
GO:0050789 (2.03e-17) regulation of biological process 78/432 | 2773/54971
GO:0009987 (3.92e-07) cellular process 146/432 | 10766/54971
GO:0008152 (0.000107) metabolic process 153/432 | 12784/54971
GO:0050794 (3.93e-16) regulation of cellular process 72/432 | 2639/54971
GO:0044281 small molecule metabolic process
GO:0044237 (7.8e-08) cellular metabolic process 129/432 | 8922/54971
GO:0006807 (3.3e-11) nitrogen compound metabolic process 80/432 | 3951/54971
GO:0019222 (1.18e-18) regulation of metabolic process 71/432 | 2223/54971
GO:0044238 (3.54e-05) primary metabolic process 131/432 | 10360/54971
GO:0009058 (9.61e-08) biosynthetic process 76/432 | 4447/54971
GO:0043170 (1.99e-07) macromolecule metabolic process 121/432 | 8393/54971
GO:0050896 response to stimulus
GO:0042221 (0.00346) response to chemical stimulus 11/432 | 383/54971
GO:0009719 (1.08e-10) response to endogenous stimulus 10/432 | 49/54971
GO:0010033 (1.08e-10) response to organic substance 10/432 | 49/54971
GO:0006730 (0.00823) one-carbon metabolic process 5/432 | 89/54971
GO:0031323 (6.81e-17) regulation of cellular metabolic process 65/432 | 2115/54971
GO:0051171 (1.19e-16) regulation of nitrogen compound metabolic process 63/432 | 2042/54971
GO:0006139 (1.04e-12) nucleobase, nucleoside, nucleotide and nucleic acid metabolic process 76/432 | 3388/54971
GO:0080090 (3.57e-17) regulation of primary metabolic process 67/432 | 2166/54971
GO:0044249 (1.12e-07) cellular biosynthetic process 72/432 | 4144/54971
GO:0009889 (4.11e-17) regulation of biosynthetic process 65/432 | 2079/54971
GO:0060255 (1.18e-18) regulation of macromolecule metabolic process 71/432 | 2196/54971
GO:0009059 (5.19e-10) macromolecule biosynthetic process 69/432 | 3393/54971
GO:0044260 (1.36e-10) cellular macromolecule metabolic process 119/432 | 7137/54971
GO:0019538 protein metabolic process
GO:0010467 (4.17e-12) gene expression 73/432 | 3283/54971
GO:0009725 (1.08e-10) response to hormone stimulus 10/432 | 49/54971
GO:0032259 (0.0012) methylation 5/432 | 58/54971
GO:0006793 (0.0124) phosphorus metabolic process 34/432 | 2384/54971
GO:0019219 (1.19e-16) regulation of nucleobase, nucleoside, nucleotide and nucleic acid metabolic process 63/432 | 2042/54971
GO:0031326 (4.11e-17) regulation of cellular biosynthetic process 65/432 | 2079/54971
GO:0016070 (4.58e-12) RNA metabolic process 47/432 | 1599/54971
GO:0010556 (4.11e-17) regulation of macromolecule biosynthetic process 65/432 | 2079/54971
GO:0034645 (4.94e-10) cellular macromolecule biosynthetic process 69/432 | 3386/54971
GO:0010468 (1.18e-18) regulation of gene expression 69/432 | 2107/54971
GO:0044267 cellular protein metabolic process
GO:0043412 (0.00129) macromolecule modification 41/432 | 2674/54971
GO:0006796 (0.0124) phosphate metabolic process 34/432 | 2384/54971
GO:0051252 (4.77e-17) regulation of RNA metabolic process 46/432 | 1095/54971
GO:0032774 (1.19e-16) RNA biosynthetic process 46/432 | 1138/54971
GO:0006350 (1.49e-15) transcription 63/432 | 2165/54971
GO:0006464 (0.0096) protein modification process 37/432 | 2611/54971
GO:0016310 phosphorylation
GO:0045449 (1.15e-16) regulation of transcription 63/432 | 2031/54971
GO:0006351 (1.19e-16) transcription, DNA-dependent 46/432 | 1134/54971
GO:0043687 (0.0096) post-translational protein modification 34/432 | 2339/54971
GO:0006468 (0.0495) protein amino acid phosphorylation 28/432 | 2043/54971
GO:0006355 (4.16e-17) regulation of transcription, DNA-dependent 46/432 | 1088/54971
Significance levels and Arrow types Diagram
Level9
Level8
Level7
Level6
Level5
Level4
Level3
Level2
Level1
is_a
part_of
positive_regulate
regulate
negative_regulate
two significant nodes
one significant node
B
GO:0003674 molecular_function
GO:0005215 transporter activity
GO:0003824 catalytic activity
GO:0030528 (5.45e-08) transcription regulator activity 36/432 | 1354/54971
GO:0005488 (8.07e-10) binding 214/432 | 15324/54971
GO:0009055 (0.00647) electron carrier activity 9/432 | 281/54971
GO:0022857 transmembrane transporter activity
GO:0022892 substrate-specific transporter activity
GO:0016740 transferase activity
GO:0016491 (0.0365) oxidoreductase activity 30/432 | 2166/54971
GO:0003676 (5.63e-17) nucleic acid binding 102/432 | 4380/54971
GO:0043167 ion binding
GO:0001882 (0.00203) nucleoside binding 57/432 | 4086/54971
GO:0000166 (0.016) nucleotide binding 61/432 | 4986/54971
GO:0005515 protein binding
GO:0022891 substrate-specific transmembrane transporter activity
GO:0016772 transferase activity, transferring phosphorus-containing groups
GO:0003677 (4.81e-28) DNA binding 94/432 | 2590/54971
GO:0043169 cation binding
GO:0001883 (0.00203) purine nucleoside binding 57/432 | 4086/54971
GO:0017076 (0.00416) purine nucleotide binding 58/432 | 4351/54971
GO:0032553 (0.00574) ribonucleotide binding 55/432 | 4154/54971
GO:0046983 (0.00574) protein dimerization activity 13/432 | 528/54971
GO:0015075 ion transmembrane transporter activity
GO:0016301 kinase activity
GO:0016773 (0.0364) phosphotransferase activity, alcohol group as acceptor 31/432 | 2252/54971
GO:0003700 (1.47e-12) transcription factor activity 35/432 | 861/54971
GO:0043565 (0.000636) sequence-specific DNA binding 17/432 | 631/54971
GO:0046872 metal ion binding
GO:0030554 (0.00203) adenyl nucleotide binding 57/432 | 4086/54971
GO:0032555 (0.00574) purine ribonucleotide binding 55/432 | 4154/54971
GO:0008324 cation transmembrane transporter activity
GO:0004672 (0.0492) protein kinase activity 28/432 | 2047/54971
GO:0046914 transition metal ion binding
GO:0032559 (0.00284) adenyl ribonucleotide binding 54/432 | 3895/54971
GO:0046873 (0.0492) metal ion transmembrane transporter activity 5/432 | 138/54971
GO:0004674 (0.0249) protein serine/threonine kinase activity 27/432 | 1819/54971
GO:0004713 (0.0423) protein tyrosine kinase activity 26/432 | 1823/54971
GO:0005507 (1.65e-19) copper ion binding 22/432 | 143/54971
GO:0005524 (0.00284) ATP binding 54/432 | 3891/54971
C
GO:0005575 cellular_component
GO:0005623 (3.83e-05) cell 100/432 | 7550/54971
GO:0044464 (3.83e-05) cell part 100/432 | 7550/54971
GO:0005622 (5.12e-08) intracellular 84/432 | 5067/54971
GO:0044424 (1.09e-10) intracellular part 81/432 | 4171/54971
GO:0043226 (1.86e-13) organelle 76/432 | 3299/54971
GO:0043229 (1.86e-13) intracellular organelle 76/432 | 3299/54971
GO:0043227 (3.08e-15) membrane-bounded organelle 68/432 | 2503/54971
GO:0043231 (3.08e-15) intracellular membrane-bounded organelle 68/432 | 2485/54971
GO:0005634 (2.89e-17) nucleus 65/432 | 2053/54971

Figure S3. Graphical results of GO analysis of the targets of the differentially expressed known miRNAs. Targets with GO terms corresponding to (A) biological processes, (B) molecular functions and (C) cellular components. The GO terms and their serial numbers are represented as boxes. For each significant term, the box includes the GO term, adjusted *p*-value (in parentheses), item number mapping the GO term in the query list and background, and the total numbers of items in the query list and background. The color scale indicates the *p*-value cutoff levels for each GO term, where a higher statistical significance corresponding to a darker and redder the color.
