# Supplementary material for: Identification and Expression Analysis of microRNAs at the Grain Filling Stage in Rice(Oryza sativa L.)via Deep Sequencing
Source: PLoS One. 2013 Mar 1;8(3):e57863. doi: 10.1371/journal.pone.0057863 (PMC3585941; doi:10.1371/journal.pone.0057863)
Supplement: Table S1 — Primers used in this study. (PDF) [file pone.0057863.s004.pdf]

Table S1. Primers used in this study.

**Primers for novel miRNA cloning**		
miRNA_ID	Forward Primer(5'-3')	Stem-loop RT Primer(5'-3')
miR-2	GCCGCGACGGCTCTCAG	GTCGTATCCAGTGCAGGGTCCGAGGTATTCGCACTGGATACGACcacccg
miR-8	GCCGCCCTGGACGGG	GTCGTATCCAGTGCAGGGTCCGAGGTATTCGCACTGGATACGACcggcca
miR-12	GCCGCTACATTTGGAACCA	GTCGTATCCAGTGCAGGGTCCGAGGTATTCGCACTGGATACGACgtccct
miR-25	GCCGCCTTTGAGTAGGG	GTCGTATCCAGTGCAGGGTCCGAGGTATTCGCACTGGATACGACctctgt
miR-35	GCCGCATCTAGTAATTGGATAG	GTCGTATCCAGTGCAGGGTCCGAGGTATTCGCACTGGATACGACtgtcct
Universal Reverse Primer	GTGCAGGGTCCGAGGT	
**Primers for mature miRNA and pre-miRNA cloning**		
miRNA_ID	Forward Primer(5'-3')	Stem-loop RT Primer(5'-3')
miR156k	GCCGCTGACAGAAGAGAG	GTCGTATCCAGTGCAGGGTCCGAGGTATTCGCACTGGATACGACgtgctc
miR164c	GCCGCTGGAGAAGCAG	GTCGTATCCAGTGCAGGGTCCGAGGTATTCGCACTGGATACGACtgcacg
miR166j	GCCGCTCGGANCAGGC	GTCGTATCCAGTGCAGGGTCCGAGGTATTCGCACTGGATACGACgaggaa
miR166m	GCCGCTCGGACCAGG	GTCGTATCCAGTGCAGGGTCCGAGGTATTCGCACTGGATACGACagggat
miR444f	GCCGCTGCAGTTGTTG	GTCGTATCCAGTGCAGGGTCCGAGGTATTCGCACTGGATACGACaagctt
miR1861a	GCCGCGATCTTGAGGC	GTCGTATCCAGTGCAGGGTCCGAGGTATTCGCACTGGATACGACctcagt
Universal Reverse Primer	GTGCAGGGTCCGAGGT	
pre-miR164cF	AAGGCCATGGTGGAGAAG	
pre-miR164cR	AGGTTCTTGTTGGAGAAGC	
pre-miR166jF	GGTGTGAGGAATGAAGCCTG	
pre-miR166jR	AGATAGGTGTTTGGAATGCAG	
pre-miR166mF	ATGCCTGATGGTATTAACGC	
pre-miR166mR	CTCTGCTTTGGTGGTTGG	
pre-miR1861aF	GACAGGAACCGTGTCGC	
pre-miR1861aR	TTGTGTTGCATATTCTTAGGCC	
**Primers for real-time quantitative RT-PCR (miRNA)**		
miRNA_ID	Forward Primer(5'-3')	Stem-loop RT Primer(5'-3')
miR156a	GCCGCTGACAGAAGAGAG	GTCGTATCCAGTGCAGGGTCCGAGGTATTCGCACTGGATACGACgtgctc
miR164e	GCCGCTGGAGAAGCAG	GTCGTATCCAGTGCAGGGTCCGAGGTATTCGCACTGGATACGACctcacg
miR166a	GCCGCTCGGACCAGG	GTCGTATCCAGTGCAGGGTCCGAGGTATTCGCACTGGATACGACggggaa
miR167d	GCCGCTGAAGCTGCCA	GTCGTATCCAGTGCAGGGTCCGAGGTATTCGCACTGGATACGACcagatc
miR168a	GCCGCTCGCTTGGTG	GTCGTATCCAGTGCAGGGTCCGAGGTATTCGCACTGGATACGACgtcccg
miR1861i	GCCGCCGATCTTGAGG	GTCGTATCCAGTGCAGGGTCCGAGGTATTCGCACTGGATACGACctcagt
Universal Reverse Primer	GTGCAGGGTCCGAGGT	
U6F	CAACGGATATCTCGGCTCT	
U6R	CAACTTGCGTTCAAAGACTC	
**Primers for real-time quantitative RT-PCR (target)**		
Target_ID	Primer (5’-3’)	
LOC_Os08g39890F	TTTGGCATCACGCTACGG	
LOC_Os08g39890R	TTACGCTGCTTGGAACCCT	
LOC_Os12g41680F	CCTGAGGGACCGAAAATAC	
LOC_Os12g41680R	GCCCTCCCCTGGTAGAAC	
LOC_Os03g01890F	AGATGCCTGGGATGAAGC	
LOC_Os03g01890R	CGGAACCAAGATGGACGA	
LOC_Os03g43930F	GAAAGCGACGGGTACTGC	

LOC_Os03g43930R	GGCTCAAGGCTCACAAGG	
LOC_Os01g63290F	TTGGGGTTATTTTGTCATTCT	
LOC_Os01g63290R	TGTCTGCATTATCTTCCGAGT	
LOC_Os04g47870F	CATTCAAATAGCACCACCG	
LOC_Os04g47870R	CAGCACGATTCACGCCAC	
LOC_Os05g51790F	GCACCACTGTCCCCAAAA	
LOC_Os05g51790R	TTCGCAAATTCACATACTCG	
actinF	TGACGGAGCGTGGTTAC	
actinR	GAGGAGCTGGTCTTGGC	
**Primers for 5'RACE**		
Target_ID	Primer(5'-3')	Comments
LOC_Os08g39890	TTGCTGGTTTGGTCGAAGGT	outer reverse primer
	ACCGGAGAAGTGGGCATGATGGCTAG	inner reverse primer
LOC_Os03g01890	GTCATTTTCTGAGCGACTACCC	outer reverse primer
	AAGTTCAACCGTTCCTCCATTTCCTGC	inner reverse primer
LOC_Os03g43930	GGATCAAATAGCCGCTAGGAAG	outer reverse primer
	TGCCGCCAAAGTTGTCGGTGCATAAG	inner reverse primer
